# Microglia‐Derived Vitamin D Binding Protein Mediates Synaptic Damage and Induces Depression by Binding to the Neuronal Receptor Megalin

**DOI:** 10.1002/advs.202410273

**Published:** 2024-12-23

**Authors:** Yan Kong, Xian Zhang, Ling Li, Te Zhao, Zihan Huang, Aini Zhang, Yun Sun, Jiao Jiao, Gaojia Zhang, Mengyu Liu, Yijun Han, Linfeng Yang, Zhijun Zhang

**Affiliations:** ^1^ Department of Neurology in Affiliated Zhongda Hospital and Jiangsu Provincial Medical Key Discipline School of Medicine Institute of Neuropsychiatry Key Laboratory of Developmental Genes and Human Disease in Ministry of Education Southeast University Nanjing 210096 China; ^2^ Department of Biochemistry and Molecular Biology School of Medicine Southeast University Nanjing Jiangsu 210009 China; ^3^ Shenzhen Key Laboratory of Precision Diagnosis and Treatment of Depression Department of Mental Health and Public Health Faculty of Life and Health Sciences Shenzhen Institute of Advanced Technology Chinese Academy of Sciences Shenzhen Guangdong 518055 China

**Keywords:** depression, megalin, microglia, neuron damage, vitamin D binding protein

## Abstract

Vitamin D binding protein (VDBP) is a potential biomarker of major depressive disorder (MDD). This study demonstrates for the first time that VDBP is highly expressed in core emotion‐related brain regions of mice susceptible to chronic unpredictable mild stress (CUMS). Specifically, the overexpression of microglia (MG)‐derived VDBP in the prelimbic leads to depression‐like behavior and aggravates CUMS‐induced depressive phenotypes in mice, whereas conditional knockout of MG‐derived VDBP can reverse both neuronal damage and depression‐like behaviors. Mechanistically, the binding of MG‐derived VDBP with the neuronal receptor megalin mediates the downstream SRC signaling pathway, leading to neuronal and synaptic damage and depression‐like behaviors. These events may be caused by biased activation of inhibitory neurons and excitatory–inhibitory imbalance. Importantly, this study has effectively identified MG‐derived VDBP as a pivotal mediator in the interplay between microglia and neurons via its interaction with the neuronal receptor megalin and intricate downstream impacts on neuronal functions, thus offering a promising therapeutic target for MDD.

## Graphical abstract

The susceptibility to stress in mice is enhanced by microglia‐derived vitamin D binding protein (VDBP). This effect is mediated through the interaction between microglia‐derived VDBP and the neuronal receptor megalin, which regulates the downstream SRC signaling pathway, leading to apoptosis, impaired synaptic function, and an excitatory/inhibitory imbalance of neurons.

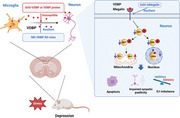

## 1 Introduction

Depression is the most common major mental disorder, ranking second in the total burden of human diseases.^[^ ^1^ ^]^ The diagnosis of depression relies on clinical symptoms and, at present, there are no reliable diagnostic or therapeutic biomarkers.^[^ ^2^ ^]^ Therefore, there is an urgent need to identify biomarkers of depression at multiple levels and elucidate their functions.

In a previous study using an extreme trait strategy combined with proteomics, bioinformatics, and machine learning, we demonstrated a selective increase of vitamin D binding protein (VDBP) in both the plasma and dorsolateral prefrontal cortex (DLPFC) of major depressive disorder (MDD) patients.^[^ ^3^ ^]^ Importantly, our subsequent findings of a selective reduction of VDBP levels in plasmatic microglia (MG)‐derived exosomes from depressed patients provide the first evidence for the value of this brain‐derived diagnostic marker for depression.^[^ ^4^ ^]^

VDBP is an evolutionarily conserved multifunctional serum α2‐globulin (Figure S1, Supporting Information), also known as group‐specific component globulin, that is an important member of the albumin family.^[^ ^5^, ^6^ ^]^ Approximately 95% of circulating VDBP is synthesized in hepatocytes.^[^ ^7^, ^8^, ^9^, ^10^ ^]^ Of which, VDBP binds to the receptor megalin as a ligand, which activates multiple downstream signal transduction pathways through cellular endocytosis, to exert its biological effects.^[^ ^11^, ^12^ ^]^ Prior studies of rats have reported that brain‐endogenous VDBP is produced by magnocellular neurosecretory cells in the hypothalamic supraoptic nucleus (SON), paraventricular nucleus (PVN), and periventricular nucleus (PEN).^[^ ^13^, ^14^ ^]^ Two preliminary studies demonstrated that VDBP mRNA can be extracted and quantified in fetal and adult brain tissue from humans^[^ ^15^ ^]^ and rats.^[^ ^16^ ^]^ However, the synthesis, distribution, physiological function, and pathogenic mechanism of brain‐endogenous VDBP in rodents, nonhuman primates, and humans remain far from being elucidated.

The present study aimed to shed light on the relationship between VDBP and depression. To do so, we first quantified the expression of VDBP in the whole brains of wild‐type mice, susceptible mice, and resilient mice induced by the chronic unpredictable mild stress (CUMS) procedure. We further analyzed the differential expression of VDBP in major nerve cell types in the prelimbic (PrL) region. Informed by the in vitro results, we then tested whether overexpression of MG‐derived VDBP in the PrL region led to depression‐like behaviors in mice and further aggravates susceptibility of mice to CUMS, but if the conditional knockout of VDBP gene in mouse MG resulted in resilience. We also explored whether MG‐derived VDBP bound to the neuronal receptor megalin and regulated key downstream signal transduction pathways, leading to synaptic damage followed by depression‐like behaviors. Finally, we investigated whether MG‐derived VDBP preferentially acted on specific neuron types in CUMS‐induced depression‐like mice. Overall, these findings suggest MG‐derived VDBP as a novel mediator for microglia–neuron communication and a promising therapeutic target for MDD.

## 2 Results

### 2.1 Whole‐Brain Expression Analysis of VDBP mRNA or Protein in Wild‐Type Mice, CUMS‐Susceptible Mice, and CUMS‐Resilient Mice

**Figure 1:**
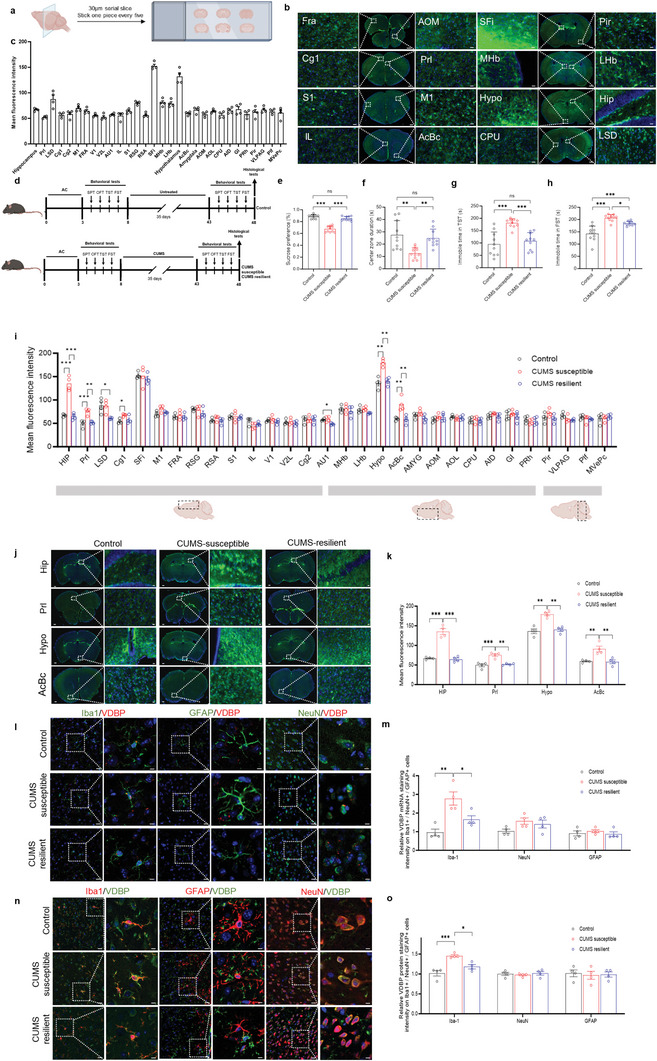
Whole‐brain expression analysis of VDBP mRNA or protein in wild‐type, CUMS‐susceptible, and CUMS‐resilient mice. a) C57BL/6J mouse brains (male mice 6–8 weeks old) were serially sectioned into 30 µm slices and immunostained with VDBP antibody. b) Representative immunofluorescence images of VDBP protein expression in various brain regions. Green: VDBP. Blue: DAPI. Scale bar = 20 or 100 µm. c) Fluorescence intensity of VDBP in each brain area of wild‐type mice. *n* = 4 mice per group. d) Experimental timeline for CUMS paradigm, behavioral tests, and preparation of brain sections. e–h) Behavioral test results for control and CUMS mice. CUMS susceptible mice showed lower sucrose preference (e), a decreased central zone duration time in the OFT (f), and increased immobility time in the TST (g) and FST (h). CUMS resilient mice behaved indistinguishably from control nonstressed mice (*n* = 10 mice per group). i) Fluorescence intensity of VDBP in each brain area of susceptible and resilient mice after the CUMS paradigm (*n* = 4 mice per group). j) Representative immunofluorescence images of VDBP protein expression in emotion‐related brain regions in control, CUMS‐susceptible, and CUMS‐resilient mice. Scale bar = 100 µm (left) and 20 µm (right). k) Fluorescence intensity of VDBP in emotion‐related brain regions in control, CUMS‐susceptible, and CUMS‐resilient mice (*n* = 4 mice per group). l,m) Representative confocal images and quantification of VDBP RNAscope signal colocalized with cell‐specific markers for neurons (NeuN), astrocytes (GFAP), and microglia (Iba1) in the PrL of control, CUMS‐susceptible, and CUMS‐resilient mice. *n* = 4 mice. Scale bar = µm 20 (left) and 10 µm (right). n,o) Representative confocal images (n) and quantification (o) of VDBP protein colocalized with cell‐specific markers for neurons (NeuN), astrocytes (GFAP), and microglia (Iba1) in the PrL of control, CUMS‐susceptible, and CUMS‐resilient mice. *n* = 4 mice. Scale bar = 20 µm (left) and 10 µm (right). Data represent the mean ± SEM. For comparisons among groups, one‐way analysis of variance (ANOVA) followed by Bonferroni post hoc tests was used. *p < 0.05, **p < 0.01, ***p < 0.001.

To our knowledge, there are no reports of whole‐brain expression of the VDBP messenger RNA (mRNA) or protein in mice. Thus, using wild‐type C57 Black 6 Jackson Laboratory (C57BL6/J) mice, we first demonstrated that VDBP protein was widely expressed in the mouse brain, including 30 regions of the forebrain, midbrain, and hindbrain (**Figure** **1**a–c). Next, mice susceptible or resilient to CUMS‐induced depressive‐like behaviors were identified (Figure 1d–h). Compared with control mice, CUMS‐susceptible mice showed significantly decreased sucrose preference rate (Figure 1e) (*p* < 0.001) and residence time in the central area of the open field test (OFT) (*p* < 0.01) (Figure 1f), and significantly increased immobility time in the tail suspension test (TST) (*p* < 0.001) (Figure 1g) and forced swimming test (FST) (*p* < 0.001) (Figure 1h). By contrast, there was no significant difference between control and resilient mice (*p* > 0.05). Brain regions showing significantly increased VDBP protein in CUMS‐susceptible mice included the hippocampus (*p* < 0.001), PrL (*p* < 0.001), hypothalamus (*p* < 0.01), and nucleus accumbens core (AcbC) (*p* < 0.01) (Figure 1i–k), suggesting that VDBPs in core regions of emotion circuits are associated with chronic‐stress‐induced depression‐like behaviors.

Our previous study reported altered VDBP levels in the DLPFC of depressive patients^[^ ^3^ ^]^ and in the PrL of mice.^[^ ^4^ ^]^ Further analysis using Gene Expression Omnibus (GEO) datasets showed that VDBP gene expression in the postmortem prefrontal cortex was significantly higher in the depressive patient group (*n* = 72) compared to the control group (*n* = 94; log_2_fold change (FC) case/control = 0.046, false discovery rate (FDR) *q* = 0.023) (Figure S2, Supporting Information). Therefore, we primarily focused on the PrL region in subsequent experiments. The results showed that the VDBP mRNA (Figure 1l,m) and protein (Figure 1n,o) were expressed in neurons, astrocytes, and MG of the above three groups of mice, suggesting that nerve cells in the brain can produce VDBP endogenously. CUMS‐susceptible mice showed significantly higher expression of VDBP mRNA (*p* < 0.01) (Figure 1m) and protein (*p* < 0.001) (Figure 1o) in MG compared to control mice. By contrast, VDBP expression was significantly reversed in CUMS‐resilient mice (*p* < 0.05). These results suggest that a significant increase of MG‐derived VDBP in the PrL may be involved in the development of depression‐like behaviors in mice.

### 2.2 Effects of MG‐Derived VDBP Overexpression in the PrL Region on Depressive‐Like Behavior and Susceptibility to CUMS in Mice

To reveal the effects of increased MG‐derived VDBP on neurons and synapses, BV2 cells or primary mouse MG were treated with dexamethasone (DX) to establish a stressed‐MG model. DX could promote neuroinflammation or inhibit inflammatory responses of microglia.^[^ ^17^, ^18^, ^19^ ^]^ DX‐treated primary mouse microglia or BV2 cells as in vitro stressed cell models were widely used in depression research.^[^ ^20^, ^21^, ^22^ ^]^ DX significantly increased the VDBP expression at mRNA level (Figure S3a, Supporting Information) (*p* < 0.01 or *p* < 0.05), protein level (*p* < 0.05 or *p* < 0.001) (Figure S3b, Supporting Information), and secretion level (both *p* < 0.05) (Figure S3c, Supporting Information) in both BV2 and primary MG. Primary neurons treated with the conditioned medium of DX‐treated BV2 or primary MG showed significantly decreased neuronal viability (both *p* < 0.01) (Figure S3d, Supporting Information), increased neuronal apoptosis (both *p* < 0.001) (Figure S3e,f, Supporting Information), decreased synapse‐associated proteins postsynaptic density protein95 (PSD95) and synapsin I (both *p* < 0.01) (Figure S3g, Supporting Information), and impaired dendrite structures (both *p* < 0.01) (Figure S3h–j, Supporting Information).

We then examined if the increase in MG‐derived VDBP induced neuronal and synaptic damage by overexpression of VDBP in BV2 or primary MG (Figure S4a,b, Supporting Information). Conditioned medium from VDBP overexpressing BV2 or primary MG significantly reduced survival rate (*p* < 0.05 or *p* < 0.01) (Figure S4c, Supporting Information), increased apoptosis (*p* < 0.05 or *p* < 0.01) (Figure S4d, Supporting Information), decreased expression levels of PSD95 and synapsin I (*p* < 0.05 or *p* < 0.01) (Figure S4e, Supporting Information), and impaired dendrite structures (*p* < 0.001 or *p* < 0.01) (Figure S4f–i, Supporting Information) of primary mouse neurons. We further employed purified mouse VDBP protein at the same concentration as in the cerebrospinal fluid of psychiatric patients^[^ ^23^ ^]^ to further verify the direct effect of VDBP on neurons (Figure S5, Supporting Information). These results suggest that the VDBP protein can induce neuronal and synaptic toxic effects.

Subsequently, we examined whether targeted knockdown of MG‐derived VDBP can correct neuronal toxic effects induced by stressed MG medium. The knockdown of VDBP (Figure S6a,b, Supporting Information) significantly reversed neuronal and synaptic damage induced by DX‐stressed MG medium, as shown by increased neuronal viability (*p* < 0.01) (Figure S6c,e, Supporting Information), decreased neuronal apoptosis (*p* < 0.01) (Figure S6d,f), increased levels of the synaptic proteins PSD95 (*p* < 0.01) and synapsin I (*p* < 0.01) (Figure S6g,h, Supporting Information), and rescued dendrite length (*p* < 0.01) and complexity (*p* < 0.01) (Figure S6i–k, Supporting Information). This counterevidence validation provides further support that targeted knockdown of MG‐derived VDBP can reverse synaptic damage caused by conditioned medium from DX‐stressed MG.

**Figure 2:**
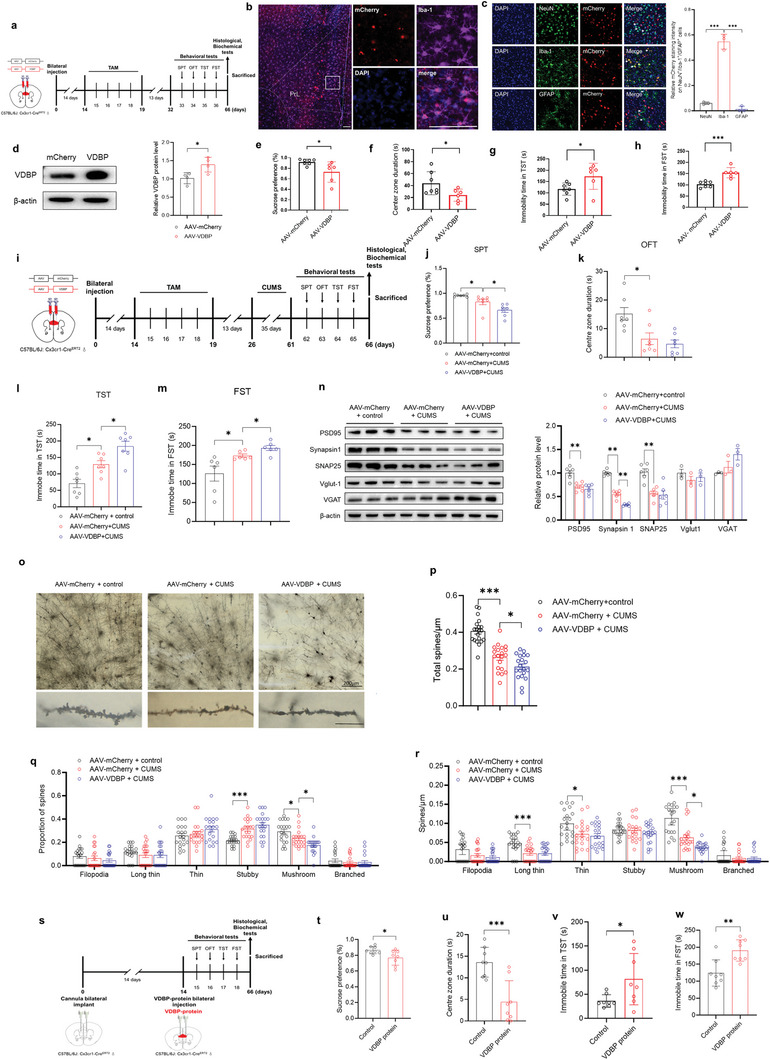
Effects of specific overexpression of MG‐derived VDBP in the PrL region on CUMS‐induced depressive‐like behavior and neuronal synapses in mice. a) Schematic of the experimental design. TAM, tamoxifen. b) Immunofluorescent assay showed the efficient infection of MG in mouse PrL. Red: mCherry. Blue: DAPI. Purple: Iba‐1. Scale bar: left 50 µm, right 20 µm. c) Immunostaining and quantification results showed MG‐specific overexpression of VDBP in PrL MG cells. Blue: DAPI. Green: cell type marker. Red: mCherry. *n* = 3 mice per group. Scale bar = 20 µm. d) Western blotting with quantification of VDBP protein levels in the PrL of mice administered AAV–VDBP or AAV–mCherry by stereotactic injection. *n* = 4 biological replicates. Data were analyzed by the two‐tailed unpaired *t‐*test. e–h) Behavioral test scores of mice treated with AAV–VDBP or AAV–mCherry. Depressive behaviors were observed in sucrose preference (e) or immobility time in the TST (g) and FST (h). AAV–VDBP mice also demonstrated a decreased central zone duration time in the OFT (f). *n* = 7 mice for AAV–mCherry, *n* = 6 for AAV–VDBP. i) Schematic of the experimental design of mice treated with AAV–VDBP or AAV–mCherry and stressed by CUMS. j–m) Behavior tests of CUMS‐stressed AAV–VDBP or AAV–mCherry mice. CUMS‐stressed AAV–VDBP mice showed a decreased sucrose preference (j) and central zone duration time (k), and increased immobility time in the TST (l) and FST (m) compared with CUMS‐stressed AAV–mCherry mice. *n* = 7 mice per group for SPT, OFT, and TST. *n* = 6 for FST. n) Western blotting with quantification of synaptic protein levels in the PrL of AAV–mCherry and AAV–VDBP mice after CUMS. *n* = 3–6 mice per group. o–r) Golgi‐Cox‐stained dendritic spines and spine density quantification in the PrL of AAV–mCherry and AAV–VDBP mice after CUMS. *n* = 38–40 neurons from 5 mice per group. Filopodia spines are >2 µm in length; the maximum width of stubby spines is less than their length; long thin spines are 1–2 µm in length; thin spines are <1 µm in length; mushroom spines have a head/neck diameter ratio > 1 (q, r). Scale bar = 2 µm. s) Schematic of the experimental design. t–w) Behavioral test results for C57bl/6j mice that underwent stereotactic injection of VDBP protein into the PrL. VDBP‐treated mice showed a decreased sucrose preference (t) and central zone duration (u). Immobility time in the TST (v) and FST (w) also increased. *n* = 8 mice per group. Data represent the mean ± SEM. Student's *t*‐test was used for statistical comparisons between two groups. For comparisons among groups, one‐way ANOVA followed by Bonferroni post hoc tests was used. *p < 0.05, **p < 0.01, ***p < 0.001.

To further corroborate the role of microglial VDBP in stress‐induced neurobehavioral dysfunction, we employed a Cre‐dependent adeno‐associated virus (AAV)‐mediated overexpression strategy to construct the VDBP overexpression mouse model (AAV–VDBP), as shown in **Figure** **2**a, in which VDBP‐overexpressing (recombinant AAV‐cytomegalovirus promoter‐double‐floxed inverted open‐reading frame ‐VDBP‐2A‐mCherry‐ woodchuck hepatitis virus post‐transcriptional regulatory element‐human growth hormone poly A, rAAV–CMV–DIO–VDBP–2A‐mCherry–WPRE–hGH polyA) or control virus (rAAV–CMV–DIO–mCherry–WPRE–hGH polyA) was stereotaxically injected into the PrL region of Cx3cr1‐Cre^ERT2^ mice. Fourteen days after the virus injection, tamoxifen (100 mg kg^−1^) was administered intraperitoneally for 5 consecutive days. Behavioral assessments were performed 13 days after the final injection. Next, immunofluorescence analysis showed that VDBP was specifically and highly expressed in MG in the PrL region of AAV–VDBP group mice (Figure 2b,c), and this result was further confirmed by western blot (Figure 2d). Compared with AAV–mCherry control mice, AAV–VDBP mice had shown abnormal behaviors, as indicated by decreased residence time in the central area of the OFT (Figure 2f) (*p* < 0.05), decreased sucrose preference test (SPT) scores (*p* < 0.05) (Figure 2e), and increased immobility times in the TST (Figure 2g) (*p* < 0.05) and FST (Figure 2h) (*p* < 0.001), suggesting that overexpression of MG‐derived VDBP in PrL region can induce depression‐like behaviors in mice.

After the CUMS procedure (Figure 2i), compared with AAV–mCherry control mice, AAV–mCherry + CUMS group mice dramatically exhibited depression‐like behaviors (Figure 2j–m). Importantly, AAV–VDBP + CUMS group mice showed exacerbation of depression‐like behaviors, including significantly decreased SPT scores (Figure 2j) (*p* < 0.05), increased immobility times in the TST (*p* < 0.05) (Figure 2l) and in FST (*p* < 0.05) (Figure 2m), suggesting that the targeted overexpression of VDBP aggravates the CUMS‐induced depressive phenotypes, further revealing the importance of VDBP in the pathogenesis of depression.

Analysis of synaptic structure and function indicated that, compared to the AAV–mCherry + control group, the AAV–mCherry + CUMS group had significantly decreased levels of synapse‐associated protein synapsin I (*p* < 0.01) and PSD95 (*p* < 0.01) (Figure 2n), decreased total density of dendritic spines (*p* < 0.001) (Figure 2o,p), increased proportion of stubby subtype (*p* < 0.001), but decreased proportion of mushroom subtype (*p* < 0.05) (Figure 2q), decreased density of spines subtypes including long thin (*p* < 0.001), thin (*p* < 0.05), and mushroom (*p* < 0.001) (Figure 2r). Interestingly, the synaptic damage in the PrL region of AAV–VDBP + CUMS group mice was further aggravated, particularly in terms of the reduced synapsin I protein level (*p* < 0.01) (Figure 2n), spines of the mushroom subtype (*p* < 0.05) (Figure 2q,r). Taken together, these results suggest that overexpression of MG‐derived VDBP in the PrL region aggravates CUMS‐induced depressive‐like behavior and impairment of synaptic structure and function.

We further examined if VDBP protein injection in the PrL region directly induced depressive‐like behaviors. Metal cannulas were implanted in the bilateral PrL area of wild‐type C57BL/6J mice by brain stereotaxy, through which VDBP protein was injected (5 µg mL^−1^, 500 nL). Behavioral tests were performed the day after injection (Figure 2u). Compared with control mice injected with saline, VDBP‐protein‐treated mice showed significant differences in the SPT (*p* < 0.05) (Figure 2v), OFT (*p* < 0.05) (Figure 2w), TST (*p* < 0.05) (Figure 2x), and FST (*p* < 0.001) (Figure 2y), suggesting that exogenous VDBP can induce depression‐like behaviors in mice.

### 2.3 Resilience to CUMS‐Induced Depression‐Like Behaviors in Mice with Microglia VDBP‐Conditioned Knockout Mice

**Figure 3:**
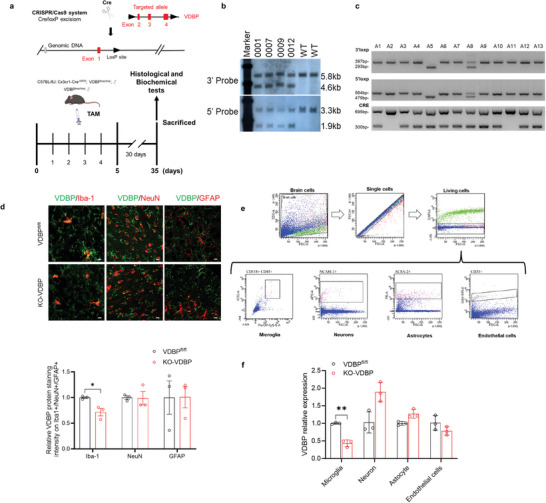
Construction and validation of MG‐derived VDBP conditional knockout mice. a) Schematic of construction of VDBP^fl/fl^ mice using the CRISPR‐Cas9 technique. The strategy for KO‐VDBP and subsequent behavioral studies is also shown. b) Southern blot of DNA from tails of F1 mice. DNA was digested using the restriction enzymes NcoI and AseI. The 3′ probe was used to detect recombination. The left homology arm (LR) probe was used to rule out random incorporation. WT: wildtype control. VDBP^fl/+^ F1 mice: 001, 007, 009, and 012. c) PCR validation of Cx3cr1‐Cre^ER/+^; VDBP^fl/fl^ mice by PCR primers for 5′loxp, 3′loxp, and Cre. A1, A3, A4, A6, A7, A9, A10, A12, and A13 are the mice with the genotype Cx3cr1‐Cre^ER/+^; VDBP^fl/fl^. d) Immunofluorescence of VDBP in different cell types of the PrL in TAM‐treated VDBP^fl/fl^ and KO‐VDBP mice. Blue: DAPI. Green: VDBP. Red: cell type marker (neuron: NeuN, microglia: Iba1, astrocyte: GFAP). Scale bar = 20 µm. *n* = 3 mice. e) Fluorescence‐activated cell sorting of microglia, neurons, astrocytes, and endothelial cells from mouse brains. f) Reverse transcriptase (RT)‐quantitative polymerase chain reaction (qPCR) of VDBP expression in different brain cell types after fluorescence‐activated cell sorting. Data were analyzed by the two‐tailed unpaired *t*‐test and expressed as the mean ± SEM, *n* = 3 mice. Student's *t*‐test was used for statistical comparisons between two groups. *p < 0.05, **p < 0.01, ***p < 0.001.

To further confirm the role of VDBP in CUMS‐induced depression‐like behavior, we used clustered regularly interspaced short palindromic repeats/CRISPR‐associated protein 9 (CRISPR/Cas9) to construct MG conditional VDBP gene knockout mice. First, VDBP^fl/fl^ tool mice were constructed (**Figure** **3**a). After validating the positive F1 hybrid flox mice (VDBP ^fl/+^) (Figure 3b), they were further crossed with MG‐specific inducible Cre enzyme expression mice (Cx3cr1‐Cre^ERT2^) to obtain the Cx3cr1‐Cre^ERT2/+^; VDBP^fl/fl^ genotype (Figure 3c). After intraperitoneal injection of tamoxifen (100 mg kg^−1^) for 5 days to mice with this genotype or control mice (VDBP^fl/fl^), we finally obtained VDBP knockout mice for the whole brain MG (KO‐VDBP) and control mice (VDBP^fl/fl^). MG‐specific deletion of VDBP in KO‐VDBP mice was confirmed by immunofluorescence analysis of PrL brain tissue slices (*p* < 0.05) (Figure 3d). The knockout efficiency in PrL microglia was much more obvious after CUMS as indicated by immunohistochemistry (Figure S7, Supporting Information). To further confirm the knockout efficiency and cell specificity, different cell types from mouse cortical tissue were sorted by flow cytometry (Figure 3e). RNA was then extracted from each cell type and the VDBP mRNA levels were detected. The results confirmed that MG‐derived VDBP was specifically knocked out in KO‐VDBP mice (*p* < 0.01) (Figure 3f). Altogether, these results confirm the successful construction of the mouse model of MG conditional VDBP gene knockout.

**Figure 4:**
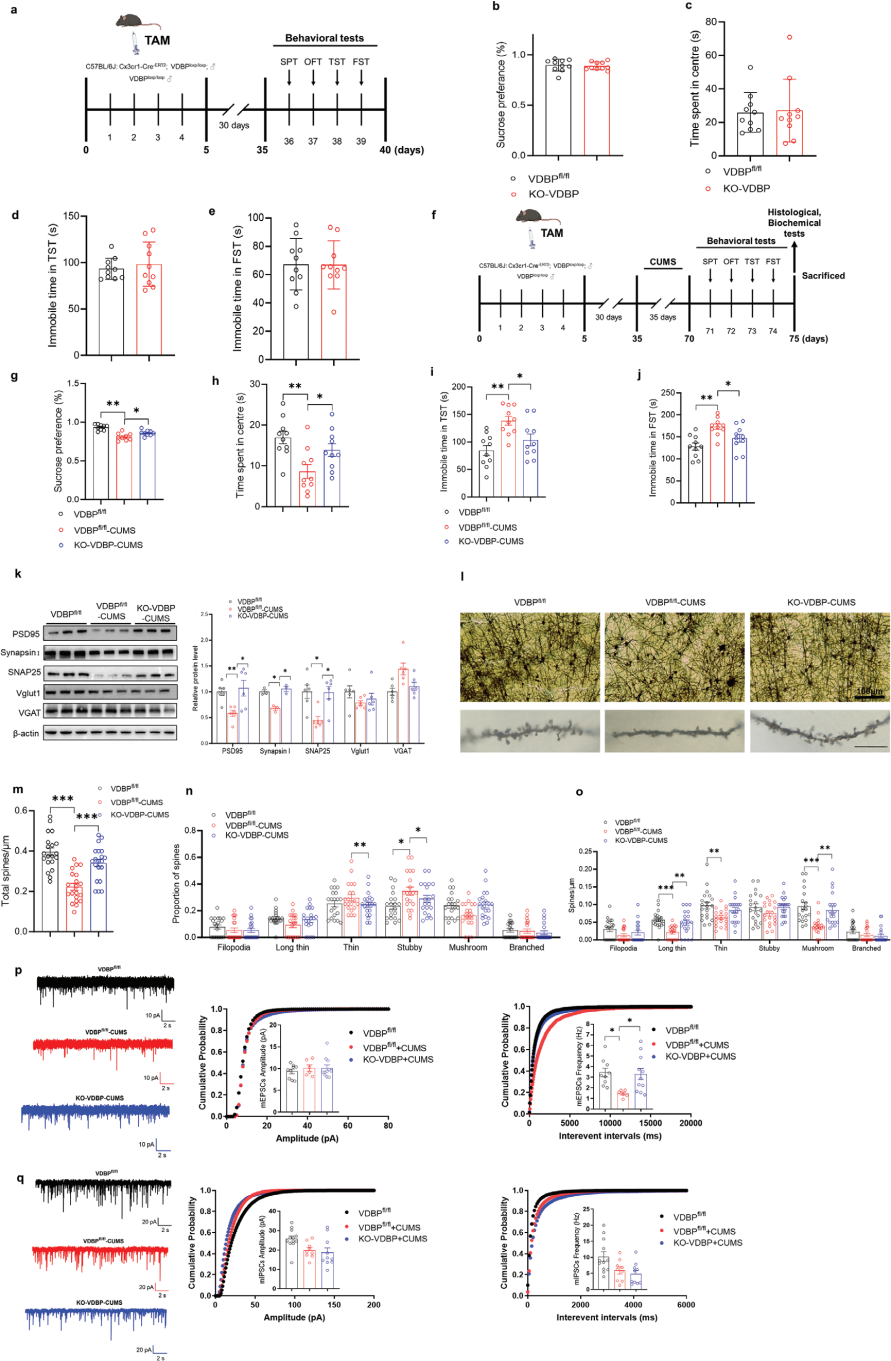
Resistance to CUMS‐induced depressive‐like behavior in mice with microglia VDBP‐conditioned knockout mice. a) Schematic of the experimental design. TAM, tamoxifen. b–e) Behavior test results for VDBP^fl/fl^ and Cx3cr1‐Cre^ER/+^; VDBP^fl/fl^ mice. No significant differences were observed in sucrose preference (b), central zone duration (c), or immobility time in the TST (d) and FST (e). *n* = 10 mice per group. f) Schematic of the experimental design of VDBP^fl/fl^ and KO‐VDBP mice stressed by CUMS. g–j) Behavioral test results for CUMS‐stressed VDBP^fl/fl^ and KO‐VDBP mice. CUMS‐stressed KO‐VDBP mice showed an increased sucrose preference (g) and central zone duration time (h) and decreased immobility time in the TST (i) and FST (j) compared with CUMS‐stressed VDBP^fl/f^ mice. *n* = 10 mice per group. k) Western blotting with quantification of synaptic protein levels in the PrL of VDBP^fl/fl^ and KO‐VDBP mice after CUMS. *n* = 6 biological replicates. l–o) Golgi‐Cox stained dendritic spines and spine density quantification in the PrL of VDBP^fl/fl^ and KO‐VDBP mice after CUMS. *n* = 38–40 neurons from five mice per group. Filopodia spines are >2 µm in length; the maximum width of stubby spines is less than the length; long thin spines are 1–2 µm in length; thin spines are <1 µm in length; mushroom spines have a head/neck diameter ratio > 1 (n, o). Scale bar = 2 µm. p) Representative mEPSC traces. Scale bar = 10 pA, 2 s (left). Cumulative distribution plot and bar graph showing the mEPSC amplitude of PrL pyramidal neurons from VDBP^fl/fl^ + control, VDBP^fl/fl^ + CUMS, and KO‐VDBP + CUMS mice (middle). Cumulative distribution plot of the mEPSC interevent interval and bar graph of mEPSC frequency of PrL pyramidal neurons from VDBP^fl/fl^ + control, VDBP^fl/fl^ + CUMS, and KO‐VDBP + CUMS mice (right). Data were expressed as the mean ± SEM, *n* = 3 mice/10 cells per group. q) Representative mIPSC traces. Scale bar = 20 pA, 2 s (left). Cumulative distribution plot and bar graph showing the mIPSC amplitude of PrL pyramidal neurons from VDBP^fl/fl^ + control, VDBP^fl/fl^ + CUMS, and KO‐VDBP + CUMS mice (middle). Cumulative distribution plot of the mEPSC interevent interval and bar graph of the mIPSC frequency of PrL pyramidal neurons from VDBP^fl/fl^ + control, VDBP^fl/fl^ + CUMS, and KO‐VDBP + CUMS mice (right). Data were expressed as the mean ± SEM. n = 3 mice/10 cells per group. Student's *t*‐test was used for statistical comparisons between two groups. For comparisons among groups, one‐way ANOVA followed by Bonferroni post hoc tests was used. *p < 0.05, **p < 0.01, ***p < 0.001.

Initial behavioral assessment (**Figure** **4**a) showed no significant differences in SPT, OFT, TST, or FST scores (*p* > 0.05) (Figure 4b–e) between VDBP^fl/fl^ control mice and KO‐VDBP mice. Interestingly, after induction of CUMS (Figure 4f), there was a significant reversal of the reduced SPT (*p* < 0.05) (Figure 4g) and a prolonged TST (*p* < 0.05) (Figure 4i) and FST (*p* < 0.05) (Figure 4j) immobility time in KO‐VDBP + CUMS mice. Analysis of synaptic structure and function of the PrL region showed that, in KO‐VDBP + CUMS mice, there was a marked reversal of the reduction in expression of synapse‐associated proteins (PSD95, synapsin1, synaptosome associated protein 25 (SNAP25)) (*p* < 0.05) (Figure 4k), total dendritic spine density (*p* < 0.001) (Figure 4l,m), the proportion of stubby spines (*p* < 0.05) (Figure 4n), and the density of spine subtypes including long thin and mushroom (*p* < 0.01) (Figure 4o). Importantly, KO‐VDBP + CUMS mice showed a reversal of the decrease in the frequency of miniature excitatory postsynaptic current (mEPSC) (*p* < 0.05) (Figure 4p), but not miniature inhibitory postsynaptic current (mIPSC) (Figure 4q). In conclusion, conditional deletion of VDBP in MG significantly improved depressive‐like behavior and synaptic impairment in the PrL region of CUMS mice.

### 2.4 MG‐Derived VDBP Binds to the Neuronal Receptor Megalin and Regulates SRC Downstream Signaling Pathways, Leading to Synaptic Damage and Depressive‐Like Behaviors

Previous studies have confirmed that circulating VDBP can act as a ligand and bind to the cell membrane receptor megalin in various organs and tissues, which mediates the activation of multiple downstream signaling pathways by increasing cell endocytosis and plays crucial biological roles.^[^ ^12^, ^24^ ^]^ However, the binding of VDBP to its receptor megalin in neurons has not yet been reported. To verify the binding of VDBP to the neuronal receptor megalin and activation of the critical pathway, we first performed the in vitro experiments described in detail in Figure S8 (Supporting Information).

To verify the binding of VDBP to the neuronal receptor megalin in vitro, we performed the following three experiments (Figure S8a–c, Supporting Information). First, pull‐down experiments using glutathione S‐transferase (GST)‐labeled VDBP protein showed direct binding of VDBP to the neuronal receptor megalin in primary mouse neuron lysate (Figure S8a, Supporting Information). The proximity ligation assay (PLA), as a sensitive technique, was used to elucidate protein–protein interactions.^[^ ^25^ ^]^ The PLA results showed direct endogenous binding of VDBP and megalin on microtubule associated protein 2 (MAP2)‐positive primary mouse neurons (*p* < 0.01) Figure S8b, Supporting Information). Finally, we added fluorescent (Alexa)‐labeled purified VDBP protein to the culture medium of primary mouse neurons. After incubation, immuno‐cytofluorescence assays confirmed that VDBP bound to neurons and was significantly inhibited by the addition of the megalin blocker receptor‐associated protein (RAP) (*p* < 0.01) (Figure S8c, Supporting Information). Taken together, these results provide the first evidence that VDBP binds to megalin in neurons.

Subsequently, we used primary mouse neurons to screen the downstream signaling pathways regulated by MG‐derived VDBP upon binding to megalin. Western blot analysis of primary neurons treated with the medium of DX‐treated BV2 cells showed that the binding of VDBP to neuronal megalin mediated a significant decrease in the phosphorylation levels of key molecules of the downstream SRC signaling pathway, namely SRC‐p (*p* < 0.01), AKT (AKT serine/threonine kinase, also known as protein kinase B) (*p* < 0.05), and extracellular signal‐regulated kinase 1/2 (ERK1/2) (*p* < 0.01) (Figure S8d,e, Supporting Information). In addition, the proapoptotic protein Bcl‐2 associated X‐protein (BAX) was increased (*p* < 0.01) while the antiapoptotic protein B‐cell lymphoma 2 (BCL‐2) was decreased (*p* < 0.01) (Figure S8d,e, Supporting Information). Further apoptosis experiments using the terminal deoxynucleotidyl transferase dUTP nick‐end labeling (TUNEL) assay and transmission electron microscopy (TEM) consistently showed a significant increase in neuronal apoptosis (*p* < 0.01) (Figure S8f,g, Supporting Information); specifically, TEM revealed apoptotic cells indicated by nuclear fragmentation, sparse and fine‐granular chromatin (Figure S8g, Supporting Information, red arrow), irregular distribution, unclear boundaries, destruction of organelle structure, and incomplete cell membranes (Figure S8g, Supporting Information, green arrow). Importantly, these changes were significantly reversed by the addition of the megalin blocker RAP (*p* < 0.01 for all) (Figure S8d–g, Supporting Information).

Regarding the key molecules of other pathways, levels of the synapse‐associated proteins PSD95 and SNAP25 were markedly reduced by DX‐treated BV2 medium (*p* < 0.05) (Figure S8h, Supporting Information); however, there were no changes in N‐methyl‐D‐aspartate receptor (NMDAR), vesicular glutamate transporter (VGLUT), or phosphorylated signal transducers and activators of transcription (STAT‐3‐p) levels (Figure S8h, Supporting Information). Dendritic structure analysis showed reduced dendrite complexity (*p* < 0.001) (Figure S8i,j, Supporting Information), a lower average total length of dendrites per neuron (*p* < 0.01) (Figure S8k, Supporting Information), and a reduced number of dendritic branches (*p* < 0.01) could be rescued by RAP (Figure S8l, Supporting Information). Based on the combined results shown above, SRC‐mediated apoptosis and synaptic damage were selected to further elucidate the causal mechanism of depression‐like phenotypes induced by MG‐derived VDBP in mice.

**Figure 5:**
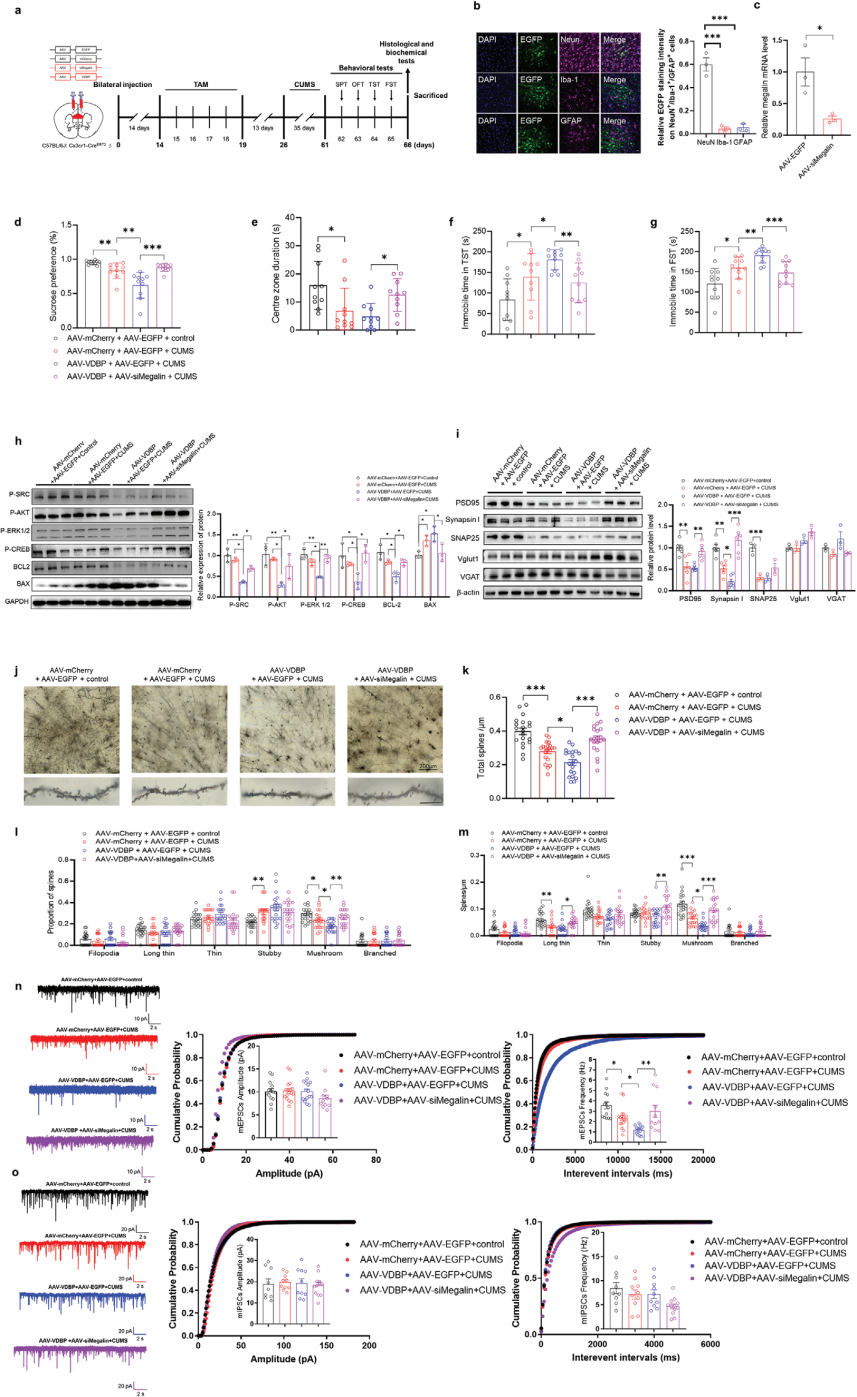
MG‐derived VDBP binds to the neuronal receptor megalin and regulates SRC downstream signaling pathways, leading to neuronal synaptic damage and depressive‐like behaviors. a) Schematic of the experimental design. TAM, tamoxifen. b) Immuofluorescent assay showed that AAV–siMegalin infected neurons specifically. Scale bar 20µm. c) RT‐qPCR showed VDBP mRNA was efficiently knocked‐down in AAV–siMegalin‐infected mice. *n* = 3 biological replicates. d–g) Behavioral test results for AAV–mCherry + AAV–EGFP + Control, AAV–mCherry + AAV–EGFP + CUMS, AAV–VDBP + AAV–EGFP + CUMS, and AAV–VDBP + AAV–siMegalin + CUMS mice. Megalin knockdown in neurons rescued behavioral alterations in SPT, OFT, TST, and FST induced by VDBP overexpression in microglia of the PrL. *n* = 10 mice per group. h) Western blotting of megalin downstream signaling molecules in the above four groups of mice. *n* = 3 biological replicates. i) Western blotting with quantification of synaptic protein levels in the PrL of the above four groups of mice. *n* = 6 biological replicates per group. j–m) Golgi‐Cox‐stained dendritic spines (j) and spine density quantification (k–m) in the PrL of the above four groups of mice. *n* = 38–40 neurons from five mice per group. Filopodia spines are >2 µm in length; the maximum width of stubby spines is less than the length; long thin spines are 1–2 µm in length; thin spines are <1 µm in length; mushroom spines have a head/neck diameter ratio > 1. Scale bar = 2 µm. n) Representative mEPSC traces. Scale bar = 10 pA, 2 s (left). Cumulative distribution plot and bar graph showing the mEPSC amplitude of PrL pyramidal neurons from AAV–mCherry + AAV–EGFP + Control, AAV–mCherry + AAV–EGFP + CUMS, AAV–VDBP + AAV–EGFP + CUMS, and AAV–VDBP + AAV–siMegalin + CUMS mice (middle). Cumulative distribution plot of the mEPSC interevent interval and bar graph of mEPSC frequency of PrL pyramidal neurons from the above four groups of mice (right). Data were expressed as the mean ± SEM. *n* = 3 animals/10 cells per group. o) Representative mIPSC traces. Scale bar = 20 pA, 2 s (left). Cumulative distribution plot and bar graph showing the mIPSC amplitude of PrL pyramidal neurons from AAV–mCherry + AAV–EGFP + Control, AAV–mCherry + AAV–EGFP + CUMS, AAV–VDBP + AAV–EGFP + CUMS, and AAV–VDBP + AAV–siMegalin + CUMS mice (middle). Cumulative distribution plot of the mIPSC interevent interval and bar graph of mIPSC frequency of PrL pyramidal neurons from AAV–mCherry + AAV–EGFP + Control, AAV–mCherry + AAV–EGFP + CUMS, AAV–VDBP + AAV–EGFP + CUMS, and AAV–VDBP + AAV–siMegalin + CUMS mice (right). Data were expressed as the mean ± SEM. *n* = 3 animals/10 cells per group. Student's *t*‐test was used for statistical comparisons between two groups. For comparisons among groups, one‐way ANOVA followed by Bonferroni post hoc tests was used. *p < 0.05, **p < 0.01, ***p < 0.001.

Based on these findings, we focused on validating the SRC signaling pathway and synaptic structural and functional changes in mice (**Figure** **5**). AAV–VDBP (rAAV–CMV–DIO–Gc–2A‐mCherry–WPRE–hGH) and AAV–siMegalin (rAAV–hsyn‐enhanced green fluorescent protein (EGFP)–5′miR‐30a–short hairpin RNA (shRNA) (Lrp2)–3′‐miR30a–WPRE–hGH polyA) or their control viruses were stereotaxically coinjected into the PrL of Cx3xr1‐Cre^ERT2^ mice. AAV–siMegalin infected neurons of PrL specifically (Figure 5b) and inhibited the expression of VDBP in PrL (Figure 5c). Two weeks later, the mice underwent intraperitoneal administration of tamoxifen (100 mg kg^−1^) for 5 consecutive days and the CUMS procedure. First, the results showed that compared with AAV–mCherry + AAV–EGFP + CUMS (CUMS group), AAV–VDBP + AAV–EGFP + CUMS (AAV–VDBP + CUMS group) mice exhibited severer depressive‐like behaviors in SPT (*p* < 0.01) (Figure 5d), TST (*p* < 0.05) (Figure 5f), and FST (*p* < 0.01) (Figure 5g). By contrast, neuron‐specific knockdown of megalin in AAV–VDBP mice followed by CUMS induction, which we term the AAV–VDBP + AAV–siMegalin + CUMS group, showed significant amelioration of depression‐like behaviors as compared with AAV–VDBP + CUMS mice (Figure 5d–g). Additionally, the effects of the SRC‐downstream pathway by the binding of MG–VDBP to neuronal megalin in the PrL region were significantly reversed (Figure 5h). The results also showed recovered synaptic impairments, as indicated by increases in the levels of synaptic proteins PSD95 (*p* <0.01), and synapsin I (*p* < 0.001) (Figure 5i); the total density of dendritic spines (*p* < 0.001) (Figure 5j,k); the density and proportion of mushroom dendritic spines (*p* < 0.001) (Figure 5l,m). Weakened ability of synaptic transmission, as manifested by a reduction in the frequency of mEPSC (*p* < 0.05) (Figure 5n) rather than those of mIPSCs (Figure 5o) were further aggravated for CUMS‐stressed VDBP overexpression mice (AAV–VDBP + AAV–EGFP + CUMS) as compared with the control mice after CUMS (AAV–mCherry + AAV–EGFP + CUMS). Interestingly, the impaired synaptic transmission capacity indicated by the frequency of mEPSCs was reversed by the knockdown of megalin (AAV–VDBP + AAV–siMegalin + CUMS) (*p* < 0.01) (Figure 5n).

Taken together, these findings support that MG‐derived VDBP binding to neuronal megalin affects the downstream SRC signaling pathway, leading to neuronal apoptosis and synaptic impairment; this effect plays a role in the occurrence of depressive‐like behaviors, and blocking megalin can reduce the effect of VDBP to exert an antidepressant effect.

### 2.5 Specificity of MG‐Derived VDBP Action on Neuronal Subtypes Related to Depression

**Figure 6:**
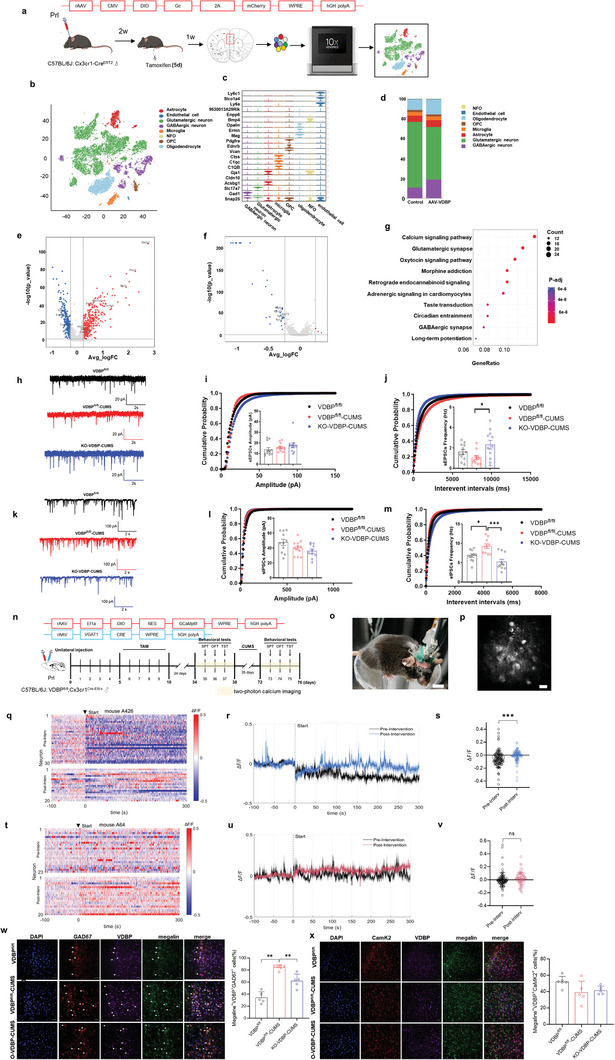
Specificity of MG‐derived VDBP action on neuronal subtypes related to depression. a) Overview of the experimental design for single‐nucleus sequencing of AAV–mCherry and AAV–VDBP mice (*n* = 3 mice per condition). b) TSNE plot of single‐nucleus sequencing of AAV–mCherry and AAV–VDBP mice. c) Average scaled expression levels of selected signature genes for different cell types. d) Stacked barplot comparing the cell‐type compositions between AAV–mCherry and AAV–VDBP mice. e) Volcano plot showing DEGs for GABAergic neurons in AAV–VDBP versus AAV–mCherry mice. Upregulated genes are highlighted in red, while downregulated genes are highlighted in blue. f) Volcano plot showing DEGs for glutamatergic neurons in AAV–VDBP versus AAV–mCherry mice. Upregulated genes are highlighted in red, while downregulated genes are highlighted in blue. g) KEGG enrichment analysis of DEGs in GABAergic neurons in AAV–VDBP versus AAV–mCherry mice. h) Sample traces of sEPSCs recorded from pyramidal neurons in VDBP^fl/fl^, VDBP^fl/fl^ + CUMS, and KO‐VDBP + CUMS mice. Scale bar = 20 pA, 2 s. i) Cumulative probability distribution for sEPSC amplitude and the mean sEPSC amplitude. j) Cumulative probability distribution of sEPSC interevent intervals and the mean sEPSC frequency. *n* = 3 mice/10 cells per group. k) Sample traces of sIPSCs recorded from pyramidal neurons in VDBP^fl/fl^, VDBP^fl/fl^ + CUMS, and KO‐VDBP + CUMS mice. Scale bar = 100 pA, 2 s. l) Cumulative probability distribution for sIPSC amplitude and the mean sIPSC amplitude. m) Cumulative probability distribution of sIPSC interevent intervals and the mean sIPSC frequency. *n* = 3 mice/10 cells per group. n) Schematic diagram showing the workflow of the head‐mounted miniature two‐photon microscope. o) Photographs of a miniature two‐photon microscope (FHIRM‐TPM V2.0) on a fingertip and mounted to the head of a mouse. Scale bar 10 mm. p) GCaMP6f imaging was taken by miniatured two‐photon microscopy, and neuronal somas were automatically identified using MATLAB script and checked manually. Scale bar: 15 mm. q) Representative heatmap of Δ*F*/*F* traces showing neuronal activity in 30 neurons from the PrL of VDBP^fl/fl^ + control mice (Pre‐Interv) and 20 neurons from the PrL of VDBP^fl/fl^ + CUMS mice (Post‐Interv) during TST. r) Example Δ*F*/*F* time‐series traces from imaging fields from VDBP^fl/fl^ + control (Pre‐Interv) and VDBP^fl/fl^ + CUMS mice (Post‐Interv) during TST. s) Average Δ*F*/*F* in neurons from VDBP^fl/fl^ + control (Pre‐Interv) and VDBP^fl/fl^ + CUMS mice (Post‐Interv) was calculated (*n* = 91 cells from 4 mice). t) Representative heatmap of Δ*F*/*F* traces showing neuronal activity in 23 neurons from the PrL of KO‐VDBP + control mice (Pre‐Interv) and 20 neurons from the PrL of KO‐VDBP + CUMS mice (Post‐Interv) during TST. u) Example Δ*F*/*F* time‐series traces from imaging fields from KO‐VDBP + control (Pre‐Interv) and KO‐VDBP + CUMS (Post‐Interv) mice during TST. v) Average Δ*F*/*F* in neurons from KO‐VDBP + control (Pre‐Interv) and KO‐VDBP + CUMS mice (Post‐Interv) was calculated (*n* = 76 cells from 3 mice). w) Immunofluorescence assay and quantification of VDBP and megalin protein expression in GABAergic neurons in the PrL of VDBP^fl/fl^ + Control, VDBP^fl/fl^ + CUMS, and KO‐VDBP + CUMS mice. *n* = 5 in the VDBP^fl/fl^ + Control group, *n* = 6 in the VDBP^fl/fl^ + CUMS group, *n* = 5 in the KO‐VDBP + CUMS group. Scale bar = 50 mm. x) Immunofluorescence assay and quantification of VDBP and megalin protein expression in glutamatergic neurons in the PrL of VDBP^fl/fl^ + Control, VDBP^fl/fl^ + CUMS, and KO‐VDBP + CUMS mice. *n* = 6 in the VDBP^fl/fl^ + Control group, *n* = 5 in the VDBP^fl/fl^ + CUMS group, *n* = 5 in the KO‐VDBP + CUMS group. Scale bar = 50 mm. Data are presented as the mean ± SEM. For comparisons among groups, one‐way ANOVA followed by Bonferroni post hoc tests was used. *p < 0.05, **p < 0.01, ***p < 0.001.

Next, we tested whether MG‐derived VDBP is biased toward specific neuronal subtypes during CUMS to induce depression. First, we performed single‐nucleus sequencing of PrL tissue from VDBP‐overexpressing mice (AAV–VDBP) and control mice (AAV–mCherry) (**Figure** **6**a). Cells in the PrL region were divided into eight subsets based on the different marker genes (Figure 6b,c). Among these subsets, the proportion of inhibitory neurons was significantly increased (Figure 6d) with the greater number of differentially expressed genes (DEGs) (UP 361, DOWN 304; Figure 6e), while the excitatory neurons with very few DEGs (UP 3, DOWN 33; Figure 6f). Kyoto Encyclopedia of Genes and Genomes (KEGG) signaling pathway analysis showed that pathways related to calcium ion signaling and synaptic activity in inhibitory neurons were significantly affected (Figure 6g), providing initial evidence that inhibitory neurons are the major target cell subtype of MG‐derived VDBP. The electrophysiological measurements shown above further suggested an imbalance between excitatory and inhibitory neurons. The full list of DEGs, gene ontology (GO), and KEGG analysis are shown in Tables S4 and S5 (Supporting Information).

Next, in vitro electrophysiological analysis of brain slices from the PrL region was performed. The results showed that compared with the VDBP^fl/fl^ + control group, the frequency of spontaneous inhibitory postsynaptic current (sIPSC) was increased in the VDBP^fl/fl^ + CUMS group (*p* < 0.05). However, the frequency of sIPSC was significantly decreased (*p* < 0.001), while the frequency of spontaneous excitatory postsynaptic current (sEPSC) was significantly reversed (*p* < 0.05) in the KO‐VDBP + CUMS group (Figure 6h–m). The sEPSCs and sIPSCs were recorded in the absence of tetrodotoxin (TTX) to block action‐potential‐dependent synaptic currents. The increase in sIPSC frequency in CUMS group may indicate an increase in terminal gamma‐aminobutyric acid (GABA) release probability or an increase in interneuron somatic/axonal excitability, which could be reversed by VDBP conditional knockout in microglia. These results suggest that conditional gene knockout of VDBP in MG protects against CUMS‐induced excitatory/inhibitory (E/I) imbalance and is biased to activating inhibitory neurons.

Importantly, a miniaturized two‐photon fluorescence microscope was used to dynamically measure the activation status of inhibitory neurons before and after the CUMS intervention in vivo (Figure 6n–p). Compared with control mice (VDBP^fl/fl^ + control), GABAergic neuronal activity in the PrL region was significantly enhanced in VDBP^fl/fl^ + CUMS mice during TST (*p* < 0.001) (Figure 6q–s). However, KO‐VDBP + CUMS mice showed no significant change during TST (Figure 6t–v), suggesting that MG‐derived VDBP can significantly activate GABAergic neurons and that targeted knockout of VDBP in MG can reverse activation to exert antidepressant effects.

Finally, immunoconfocal experiments showed that the colocalization of MG‐derived VDBP and megalin in inhibitory neurons of VDBP^fl/fl^ + CUMS mice was significantly increased compared with VDBP^fl/fl^ + control mice (*p* < 0.01). After conditional knockout of VDBP in MG, the above colocalization significantly decreased in KO‐VDBP + CUMS mice (*p* < 0.01) (Figure 6w). By contrast, the colocalization of MG‐derived VDBP and megalin in excitatory neurons did not differ significantly between the three groups (Figure 6x). In summary, these results suggest that MG‐derived VDBP mainly targets the megalin receptor of inhibitory neurons, thereby initiating the downstream SRC pathway and leading to neuronal synaptic damage, ultimately participating in the development of depression.

## 3 Discussion

This innovative study provides several novel findings. First, we demonstrated VDBP expression in the whole mouse brain. Notably, VDBP was highly expressed in the core emotion‐associated brain region of CUMS‐susceptible mice. Importantly, the expression of VDBP mRNA and protein in MG in the PrL region of these mice was significantly increased. Second, we demonstrated that high expression of MG‐derived VDBP started to cause depression‐like behaviors and aggravated the CUMS‐induced phenotypes in mice. The mechanism was that VDBP bound to neuronal receptor megalin and regulated the SRC signaling transduction pathway, resulting in neuronal apoptosis as well as synaptic damage. Third, we found preliminary evidence that MG‐derived VDBP preferentially activates GABAergic neurons in the PrL region of CUMS mice, mediating the development of depression. These findings are of significance for further elucidating the role of neuronal subtypes and specific projections targeted by MG‐derived VDBP in the development of depression.

This methodology offers several advantages. First, we detected VDBP expression in the whole brains of mice and determined the brain regions and neural cell subtypes in CUMS mice showing high VDBP expression. Second, we constructed a novel mouse strain with conditional knockout of the VDBP gene in MG, providing a useful tool to investigate mechanisms related to depression. Third, we demonstrated that MG‐derived VDBP overexpression, especially in CUMS‐induced mice, is critical for depression‐like behaviors. Fourth, we investigated the causal mechanisms of depression by focusing on the VDBP‐binding receptor megalin and the downstream signaling pathways that it activated. Fifth, we used a series of novel techniques, including single‐nucleus sequencing and in vivo microendoscopic Ca^2+^ imaging, to identify significant neuronal subtypes of VDBP, providing insights into the molecular signature of neuronal subtypes and the underlying circuit mechanisms. This application of new technology and rigorous scientific design enhances the reliability of our results.

Vitamin D or its active form calcitriol inhibited microglia activation and behavioral abnormalities in lipopolysaccharide (LPS)‐induced depression‐like mice, 6‐Hydroxydopamine (6‐OHDA)‐induced Parkinson's‐disease (PD)‐like mice, experimental autoimmune encephalomyelitis mouse model, and spontaneously hypertensive rats.^[^ ^26^, ^27^, ^28^, ^29^ ^]^ It was reported that vitamin D3 deficiency increased the risk of MDD and short‐term or long‐term supplementation of vitamin D3 could ameliorate symptoms of MDD in clinical trials.^[^ ^30^, ^31^, ^32^ ^]^ Vitamin D3 deficiency induces senescence and changes in microglia, which may contribute to the linkage between depression and aging.^[^ ^33^ ^]^ However, the findings are not consistent and vitamin D3 supplementation has not been used as a standard treatment in clinical practice. VDBP has three structural similar domains, the first of which is responsible for vitamin D3. The actin binding site is located in amino acids 373–403 spanning domain 2 and domain 3 as well as a part of domain 1. Additionally, VDBP could interact with chemokine C5a and fatty acids. Membrane binding sites have been identified in amino acids 150–172 and 379–402 of VDBP.^[^ ^6^ ^]^ Interestingly, mice lacking VDBP do not show evidence of vitamin D deficiency unless placed on a vitamin‐D‐deficient diet.^[^ ^34^ ^]^ The present study focused on the vitamin‐D‐independent role of VDBP which relied on megalin and downstream signaling pathways.

One essential contribution of this study is the finding that VDBP expression is significantly increased in four core emotion‐associated regions in CUMS mice, specifically for MG of the PrL region in CUMS mice. VDBP is an evolutionarily conserved multifunctional protein, and 95% of circulating VDBP is produced by hepatocytes.^[^ ^5^, ^7^, ^15^, ^16^ ^]^ However, there is still much to learn about the synthesis, distribution, and physiological function of brain‐derived VDBP in the brains of different species. It was only confirmed in 2009 that the VDBP gene and protein are highly expressed in magnocellular neurosecretory cells of the hypothalamus SON, PVN, and PEN of rats.^[^ ^14^ ^]^ There are also reports that VDBP is colocalized with amyloid plaques in the superior temporal cortex of Alzheimer's disease (AD) patients and the cortex of 6 months old 5 × FAD mice.^[^ ^35^, ^36^ ^]^ Other studies have reported that VDBP levels are significantly increased in the cerebrospinal fluid of patients with neuropsychiatric disorders, including AD,^[^ ^37^, ^38^, ^39^ ^]^ Parkinson's disease,^[^ ^37^ ^]^ multiple sclerosis,^[^ ^40^, ^41^, ^42^, ^43^, ^44^, ^45^, ^46^ ^]^ idiopathic temporal lobe epilepsy,^[^ ^47^ ^]^ human immunodeficiency virus 1 (HIV‐1) infection,^[^ ^48^ ^]^ and meningitis.^[^ ^49^, ^50^ ^]^ However, these prior mainly focused on screening diagnostic and therapeutic biomarkers in cerebrospinal fluid and correlated VDBP with vitamin D transport capacity. The present study is the first to provide evidence that MG‐derived VDBP is highly expressed in the PrL region of CUMS‐susceptible mice, which supports the cross‐species findings of our previous research^[^ ^4^ ^]^ and provides a basis for further work focusing on the causal mechanism of the interaction between MG‐derived VDBP and neurons in the development of depression.

The neuron–glia cell communication in the pathogenesis of neuropsychiatric disorders represents a cutting‐edge area of neuroscience research. For instance, astrocytes play a role in stress‐induced depression‐like behaviors by modulating the release of adenosine 5′‐triphosphate or l‐lactate to influence neurons.^[^ ^51^, ^52^ ^]^ However, investigations into microglia–neuron crosstalk in depression are still at an early stage.^[^ ^53^ ^]^ The novelty of our study lies in identifying microglia‐derived VDBP as a direct mediator of microglia–neuron communication involved in depression development, relying on neuronal receptor megalin and downstream SRC signaling pathway. Building upon our current findings, microglia‐derived VDBP holds promising potential as a therapeutic target for clinical translation. Furthermore, it is worth mentioning that we are currently screening for VDBP‐neutralizing antibodies and peptides that can antagonize the interaction between VDBP and megalin; these will be tested for their therapeutic effects in future research endeavors.

The main finding of this study is that overexpression of VDBP in the PrL region leads to depression‐like behavior and aggravates the CUMS‐induced phenotype via synaptic damage in mice, while conditional knockout of VDBP in MG plays a role against depression. We demonstrated that MG‐derived VDBP interacts with the neuronal receptor megalin to activate the downstream SRC signaling pathway and induce apoptosis, leading to depression. At present, the etiology and pathophysiologic mechanism of depression are not fully clarified and no diagnostic molecular biomarkers are used in clinical practice.^[^ ^2^, ^54^ ^]^ Therefore, it is particularly important to focus on functional verification of candidate molecules based on clinical findings and cross‐species validation. Megalin, which is also known as low‐density‐lipoprotein (LDL)‐receptor‐related protein 2 (LRP2), is a member of the LDL receptor family and was originally identified in the kidney.^[^ ^55^, ^56^ ^]^ Recently, megalin was shown to be expressed in neurons, glia, and the choroid plexus of the central nervous system.^[^ ^57^, ^58^, ^59^, ^60^ ^]^ Megalin has been identified as an endocytic receptor that binds and internalizes more than 60 ligands, including VDBP, lipoproteins, lipocalins, and hormones, leading to the activation of intracellular signaling pathways.^[^ ^11^, ^56^, ^61^, ^62^, ^63^, ^64^ ^]^ Based on studies of megalin knockout mice and patients with related mutations, the critical roles of megalin in human diseases have been identified.^[^ ^64^, ^65^, ^66^, ^67^, ^68^ ^]^ Megalin expressed in neurons is reported to mediate synaptic plasticity by acting on the downstream SRC signaling pathway.^[^ ^69^ ^]^ However, the direct interaction of VDBP and megalin and subsequent biological functions remain unknown. Here, we report for the first time that MG‐derived VDBP acts on neuronal megalin and the SRC signaling pathway to mediate neuron and synaptic damage, leading to depression‐like behaviors in mice. Blocking megalin by small interfering RNA (siRNA) or antagonists reversed neuronal damage and exerted antidepressant effects.

This study also preliminarily suggested that an imbalance between E/I neurons due to biased activation of inhibitory neurons in the PrL region is a critical contributor to MG‐derived VDBP's role in depression. E/I imbalance is an important molecular pathological feature of MDD.^[^ ^70^ ^]^ Upon examining the single‐nucleus sequencing raw data, we observed that megalin levels were approximately twofold higher in inhibitory neurons compared to excitatory neurons. Although the mechanism of E/I imbalance is intricate, it provides insights for identifying specific inhibitory neuronal subtypes regulated by MG‐derived VDBP in the PrL region, as well as its downstream projections. Whether the microglia‐derived VDBP targets the specific inhibitory neuron subcluster by interaction with the megalin–SRC pathway or direct binding with intracellular molecules needs to be clarified. Thus, our findings provide evidence for the use of brain‐derived VDBP as a biomarker for the diagnosis of depression and a target for intervening in the depression‐associated molecular circuit.

Our previous studies primarily focused on investigating the diagnostic potential of VDBP. We observed elevated levels of VDBP protein in both plasma and postmortem dorsolateral prefrontal cortex tissues of patients with MDD, as well as in PrL microglia from mice with LPS‐induced depression‐like symptoms.^[^ ^3^, ^4^ ^]^ Considering that liver is reported to be the main source of VDBP production in plasma,^[^ ^5^ ^]^ we further explored the presence of VDBP in brain‐derived extracellular vesicles. Interestingly, we found lower levels of VDBP in extracellular vesicles derived from plasma microglia (MDEVs) obtained from MDD patients, rhesus macaques with chronic‐glucocorticoid‐induced depression, and mice with LPS‐induced depression.^[^ ^4^ ^]^ Additionally, decreased levels of VDBP were also detected in cerebrospinal fluid (CSF) samples collected from depressed rhesus macaques. The contrasting relationship between increased levels of VDBP in brain tissue and peripheral blood versus decreased levels in CSF and plasma MDEVs suggests a potential role for VDBP as a diagnostic biomarker for MDD. However, the underlying mechanism responsible for this discrepancy remains unclear. In our thorough literature search, we discovered similar phenomena involving neurotoxic proteins such as α‐synuclein. For instance, α‐synuclein is known to be increased in the brains of PD patients but decreased either within neuron‐derived extracellular vesicles present in plasma^[^ ^71^, ^72^ ^]^ or within CSF samples.^[^ ^73^, ^74^, ^75^, ^76^ ^]^ Furthermore, it has been proposed that reduced α‐synuclein levels within CSF may result from sequestration within brain tissue and are inversely correlated with PD duration and severity.^[^ ^74^, ^76^ ^]^ The observed findings may also be attributed to the accumulation of VDBP protein within brain cells, presenting an intriguing phenomenon that warrants further comprehensive investigation in future studies.

This study is subject to some limitations. First, the roles of brain‐derived VDBP in other emotion‐associated brain regions, such as the hippocampus, hypothalamus, and nucleus accumbens, have not been examined yet. Second, this study focused on the causal mechanism by which MG‐derived VDBP binds to the neuronal receptor megalin and mediates inhibition of the downstream SCR‐p signaling pathway, leading to depression. However, the exact binding sites of the two molecules and the mechanism leading to the dynamic conformation of SRC phosphorylation have not been elucidated. Third, as shown in Figure 6, our series of experiments provides preliminary evidence that VDBP mainly acts on inhibitory neurons involved in MG‐derived VDBP‐related depression, particularly in a CUMS‐induced depression‐like rodent model. However, the neural circuits of specific molecular markers of GABAergic neuron subtypes require further investigation. Fourth, the potential of brain‐derived VDBP as a biomarker of depressive disorder needs to be verified in large independent samples to accelerate its transformation and application.

In conclusion, this study has shown that stress induces higher expression of MG‐derived VDBP in the PrL area, and combines with the neuronal receptor megalin to activate the SRC signaling pathway, leading to neuronal apoptosis, impaired synaptic function, biased activation of inhibitory neurons and, eventually, depression. This study provides evidence for further elucidation of VDBP targeting specific molecular markers of inhibitory neuron subtypes involved in the occurrence of depression and targeted interventions, further supporting the value of brain‐derived VDBP as a biomarker for the diagnosis of depressive disorder.

## 4 Experimental Section

### Construction of VDBP Conditional Knockout Mice

VDBP^fl/fl^ mice were constructed using a CRISPR/Cas9‐based approach by Biocytogen (Beijing China). Briefly, two single guide RNAs (sgRNAs) (5′ Guide: 5′‐GTGGTTCTGACTCACTGACG TGG‐3′; 3′ Guide: 5′‐TACTCTGATTACGTTCAAGTTGG‐3′) were designed using the CRISPR design tool (http://www.sanger.ac.uk/) to target either a region upstream of exon2 or downstream of exon4, and then screened for on‐target activity using a Universal CRISPR Activity Assay (Biocytogen Pharmaceuticals, Beijing). A circular donor vector was employed to minimize random integrations. The gene targeting vector containing a 5′ homologous arm, target fragment (exons 2–4), and 3′ homologous arm was used as a template to repair the double‐strand breaks generated by Cas9/sgRNA. The two loxp sites were precisely inserted in both sides of the target fragment of the VDBP gene. The T7 promoter sequence was added to the Cas9 or sgRNA template by polymerase chain reaction (PCR) amplification in vitro. Cas9 mRNA, the targeting vector, and sgRNAs were coinjected into the cytoplasm of one‐cell stage fertilized C57BL/6N eggs. The injected zygotes were then transferred into the oviducts of Kunming pseudopregnant females to generate F0 mice. After confirmed by tail genomic DNA PCR and sequencing, F0 mice with the expected genotype were mated with C57BL/6N mice to establish germline‐transmitted F1 heterozygous mice. Genotyping was performed by tail genomic PCR, Southern blot, and DNA sequencing.

VDBP^fl/fl^ mice were crossed with CX3CR1^Cre‐ER^ mice to generate CX3CR1^Cre‐ER/+^; VDBP^fl/fl^ mice. These mice were intraperitoneally injected with tamoxifen dissolved in corn oil at a dose of 100 mg kg^−1^ daily for 5 consecutive days. Cre recombination controlled by the CX3CR1 promoter was induced to obtain microglia‐specific VDBP knockout mice. Age‐matched littermates injected with vehicle or tamoxifen‐injected VDBP^fl/fl^ mice were used as controls. All animal experiments were conducted according to protocols approved both by the Institutional Animal Care and Use Committee of Southeast University (2020100106).

### Viral Vectors

The AAV vectors rAAV–CMV–DIO–Gc–2A‐mCherry–WPRE–hGH polyA, rAAV–CMV–DIO–mCherry–WPRE–hGH polyA, rAAV–hsyn‐EGFP–5′miR‐30a–shRNA (Lrp2)–3′‐miR30a–WPRE–hGH polyA, rAAV–hsyn‐EGFP–5′miR‐30a–shRNA (scramble)–3′‐miR30a–WPRE–hGH polyA, rAAV–eukaryotic elongation factor 1 alpha (Ef1a)–DIO– nuclear export sequence (NES)–Green calcium indicator 6f (GCaMP6f)–WPRE–hGH pA, and rAAV–vesicular GABA transporter 1 (VGAT1)–Cre recombinase (CRE)–WPRE–hGH pA with titers of 5.5 × 10^12^ v.g. mL^−1^ were purchased from BrainVTA (Wuhan, China).

### Stereotaxic Surgery and Virus Injection

Mice were anesthetized using 1.5% sevoflurane (Cat: LEG3923, FUJIFILM Wako Pure Chemical Corporation) and positioned in a digital‐readout stereotactic frame (Cat: 68516, RWD Life Science). After exposing the skull surface and opening the cranium using a speed dental drill, 200 nL AAV virus was bilaterally delivered into each site of the PrL using a microsyringe fitted with a 33 gauge needle and injector pump (Anterior‐Posterior (AP) =  1.9 mm, Medial‐Lateral (ML) =  ±0.3 mm, Dorsal‐Ventral (DV) =  −2.5 mm) at a steady speed of 15 nL min^−1^. VDBP‐overexpression (OE) virus (rAAV–CMV–DIO–Gc–2A‐mCherry–WPRE–hGH polyA) and control virus (rAAV–CMV–DIO–mCherry–WPRE–hGH polyA) were injected into Cx3cr1‐Cre mice. After 2 weeks of recovery, mice were intraperitoneally injected with tamoxifen dissolved in corn oil at a dose of 100 mg kg^−1^ daily for 5 consecutive days to obtain VDBP‐OE and control mice. During the procedure, each mouse's body temperature was maintained using a heat lamp, and its eyes were coated with sterile ointment to avoid corneal drying.

AAV virus with shRNA targeting megalin (rAAV–hsyn‐EGFP–5′miR‐30a–shRNA (Lrp2)–3′‐miR30a–WPRE–hGH polyA) or control virus (rAAV–hsyn‐EGFP–5′miR‐30a–shRNA (scramble)–3′‐miR30a–WPRE–hGH polyA) was coinjected with AAV–VDBP or control AAV–mCherry virus into the PrL of Cx3cr1‐Cre mice in the same manner to obtain AAV–VDBP + AAV–siMegalin mice, AAV–VDBP + AAV–EGFP mice, and AAV–mCherry + AAV–EGFP mice.

### CUMS Procedure

To establish a rodent model of depression, 5–6 weeks old male mice were randomly divided into CUMS and control groups. Mice in the CUMS group were maintained in individual cages and the CUMS treatment was performed according to published protocols^[^ ^77^ ^]^ with slight modifications. Briefly, mice were randomly treated with two types of mild stressors each day for 5 weeks. The stressors included: swimming in 45 °C water for 5 min, swimming in 4 °C water for 5 min, restraint stress for 2 h, cage shaking for 5 min, overnight illumination or stroboscopic illumination for 12 h, tail pinching for 1 min, tail suspension for 6 min, moist bedding for 12 h, rat bedding for 24 h, cage tilting for 12 h, an empty cage for 12 h, water deprivation for 12 h, and food deprivation for 12 h. CUMS mice that displayed more than a 20% change from baseline in the SPT were determined to be susceptible. By contrast, CUMS resilient mice were those that displayed less than a 20% reduction from baseline in the SPT.

According to the protocol published in STAR Protoc,^[^ ^78^ ^]^ sucrose preference tests were performed first and the mice with baseline sucrose preference below 70% were excluded from the study. Then, mice were randomly divided into control and CUMS groups. In Figure 1, further behavior tests, including OFT, TST, and FST showed no significant difference between the control and CUMS groups at baseline (Figure S9, Supporting Information). In the following experiments of other figures in the work, the sucrose preference tests were only used as baseline measurements to rule out abnormal mice before CUMS as the published protocol.^[^ ^78^ ^]^ This protocol could avoid the possibility of influence for repeated behavior tests on subsequent experiments.

### Assessment of Depressive‐Like Behavior—Open Field Test

The OFT was used to measure locomoting, spontaneous activity, and anxiety in mice. Briefly, the bottom of a plastic chamber (50 cm × 50 cm) was divided into 16 equal squares, and mice were individually placed in the central area. The behavior of the mouse was recorded for 5 min. The distance traveled, speed, and time spent in the central region were calculated using the ANY‐MAZE behavior monitoring system (Stoelting Co., Wood Dale, IL, USA).

### Tail Suspension Test

During the TST, mice were suspended by their tails with their heads kept 50 cm above the floor. Their behavior was recorded for 6 min. Immobility during the final 4 min was calculated at the immobility duration (%) using the ANY‐MAZE behavior monitoring system (Stoelting Co., Wood Dale, IL, USA).

### Forced Swimming Test

For the FST, mice were placed in a transparent cylinder (13.8 cm in diameter) filled with 23–25 °C water to a depth of 15 cm. Behavior was recorded using a video tracking system. Immobility was defined as no obvious movement except for that required to maintain the head above the water. The immobility duration during the final 4 min was calculated using the ANY‐MAZE behavior monitoring system (Stoelting Co., Wood Dale, IL, USA).

### Sucrose Preference Test

Anhedonia in mice was determined using the SPT. Control and stressed mice were acclimated to drinking from two bottles of 1% w/v sucrose solution for 48 h. The bottles were then replaced with one containing 1% w/v sucrose solution and one containing fresh water for another 48 h. For the test, mice were fasted for 24 h and then individually placed in a test cage containing one bottle filled with 1% w/v sucrose solution and one bottle filled with water for 2 h. To avoid bottle‐side preference, the position of the two bottles was changed after 1 h. Sucrose preference was calculated as the ratio of sucrose solution intake to the total intake of sucrose solution and water.

### Immunohistochemistry

After deep anesthesia with 1% pentobarbital and sequential perfusion with phosphate‐buffered saline (PBS) and 4% formaldehyde (PFA) for 5 min, the mouse brains were isolated and fixed overnight. The brains were dehydrated in gradient sucrose and embedded in Tissue‐Tek optimum cutting temperature (OCT) compound (Sakura) before being cut into 16 µm sections using a microtome (CM 1950, LEICA). For immunocytochemistry, cultured cells were seeded onto cover slips in a cell culture plate 1 day before plasmid transfection or drug treatment. The culture medium was discarded and the cells were fixed with 4% PFA. After being permeabilized with 0.2% Triton X‐100 and blocking with 0.5% bovine serum albumin (BSA), fixed brain tissues or cultured cells were incubated overnight at 4 °C with the following primary antibody: mouse anti‐VDBP (sc‐365441, Santa Cruz); rabbit anti‐ionized calcium binding adaptor molecule 1 (Iba‐1) (019‐19741, WAKO); rabbit anti‐neuronal nuclei (NeuN) (26975‐1‐AP, Proteintech); rabbit anti‐ glial fibrillary acidic protein (GFAP) (16825‐1‐AP, Proteintech); rabbit anti‐MAP2 (ab32454, Abcam); mouse anti‐megalin/LRP2 (sc‐515772, Santa Cruz); goat anti‐glutamate decarboxylase 67 (GAD67) (AF2086, R&D Systems); or goat anti‐CaM kinase II alpha Antibody (NBP1‐51945, Novus). Next, the sections were rinsed and incubated with fluorescent secondary antibodies for 2 h at room temperature (RT). Finally, the cell nuclei were stained with 4',6‐diamidino‐2‐phenylindole (DAPI) and mounted with an antifade mounting medium before analysis using an Olympus FV3000 confocal microscope. In addition, images were analyzed in Fiji/Image J and Image backgrounds were threshold but kept the same for control, CUMS susceptible, and CUMS resilient groups. Mean fluorescent intensity was calculated as the average fluorescent signal per pixel. For colocalization analysis of VDBP and cell type markers, the Coloc2 Plugin in Fiji was used to calculate the Pearson correlation coefficient after threshold adjustment via the Costes method. The colocalization values were normalized to the control group. *n* = 4 mice were used in each condition. One‐way analysis of variance (ANOVA) followed by Bonferroni post hoc tests was used.

### Golgi Staining

Golgi staining was performed using the FD Rapid Golgi Stain kit according to the manufacturer's instructions (FD Neuro Technologies). Briefly, freshly dissected mouse brains were immersed in equal volumes of solution A and B for 2 weeks in the dark at RT. Next, the brains were incubated in solution C for 4 days in the dark at RT before being sectioned using a vibratome (Leica, CM1950) at a thickness of 100 µm under −20 °C. Brain sections were then stained with a mixture of solution D, solution E, and double‐distilled water (ddH_2_O) (1:1:2). Finally, Golgi‐stained neurons from the PrL tissue samples were imaged by microscopy and analyzed using NIH ImageJ software.

### Fluorescence‐Activated Cell Sorting (FACS)

After anesthesia with 1% pentobarbital and perfusion with PBS, the mouse brains were dissected and digested in Hank's balanced salt solution (HBSS) buffer with papain (40 U mL^−1^) and collagenase I (1000 U mL^−1^) for 20 min using a Single Cell Suspension Dissociator (DSC‐400, RWD). Brain tissue lysates were filtered using a 70 µm cell strainer and resuspended in DPBS with glucose (1:1000, 20% glucose solution) and Debris Removal Solution (Miltenyi Biotec, 130‐109‐398) to remove cell debris. The brain cells were then incubated in Dulbecco's phosphate‐buffered saline (DPBS) with 0.5% bovine serum albumin and FcR Blocking Reagent (BD, 553 141) for 10 min to avoid nonspecific binding. Next, the brain cells were incubated at RT for 1 h with the following fluorescent labeled primary antibodies: fluorescein isothiocyanate anti‐mouse/human cluster of differentiation 11b (CD11b) (101205, BioLegend), PerCp‐CyTM5.5 anti‐mouse CD45 (561869, BD Pharmingen); Allophycocyanin (APC) anti‐Mouse neural cell adhesion molecule 1 (NCAM‐1)/CD56 allophycocyanin (FAB7820A‐100, R&D); Phycoerythrin (PE) anti‐mouse astrocyte cell surface antigen‐2 (ACSA‐2) (130‐116‐244, Miltenyi Biotec), or Brilliant Violet 605 anti‐mouse CD31 (102427, BioLegend). After rinsing 3 times with DPBS, the different brain cell types were sorted by BD FACSAria.

### Miniature Two‐Photon In Vivo Calcium Imaging and Data Analysis

Miniature two‐photon in vivo calcium imaging was performed according to previously published methods.^[^ ^79^ ^]^ Each mouse was anesthetized with isoflurane (1–1.5%) and placed on a stereotaxic frame with its body temperature maintained using heating pads. After the local application of xylocaine, a small square cranial window (0.5 × 0.5 mm^2^) was made above the PrL region (PrL: +2.8 AP, −0.4 ML). Next, 500 nL of mixed virus (rAAV–VGAT1–Cre–WPRE–hGH‐pA and rAAV–Ef1a–DIO–NES–GCaMP6f–WPRE–hGH‐pA) (2 × 10^13^ gc µL^−1^, 0.5–0.8 µL) was slowly injected into the PrL region for 20 min. Three weeks later, the mice were anesthetized and a homemade holder was attached to the skull. A small square cranial window (2.5 × 2.5 mm^2^) was made over the PrL and a small glass coverslip (3.5 × 2.5 mm^2^) (CG00C2, no. 0 BK‐7, 85–115 µm, Thorlabs) was placed on it. After recovery for 1 week, each mouse was trained using a dummy headpiece for 30 min daily for 3 days. A miniature two‐photon microscope (FHIRM‐TPM V2.0, field of view: 420 × 420 µm^2^; resolution: ≈1.13 µm; working distance: 1 mm) headpiece with a fiber and cable was mounted over the Gradient‐index (GRIN) lens. Imaging data were acquired using GINKGO‐MTPM imaging software (Transcend Vivoscope Biotech Co., Ltd., China) at a frame rate of 10 Hz (512 × 512 pixels) with a femtosecond fiber laser (≈35 mW at the objective, TVS‐FL‐01, Transcend Vivoscope Biotech Co., Ltd., China). GCaMP6f was excited at 920 nm.

Video preprocessing, signal processing, statistical analysis, and visualization were all conducted using MATLAB (MathWorks). For preprocessing of two‐photon calcium fluorescence imaging videos, jitter caused by mouse movement was initially corrected using a block rigid registration algorithm (NoRMCorre).^[^ ^80^ ^]^ Subsequently, neuronal soma identification was performed using a tubular detection algorithm^[^ ^81^ ^]^ and an in‐house adaptive threshold segmentation algorithm, followed by manual verification. Furthermore, neuronal calcium signals were extracted using the commonly used annular ring subtraction algorithm,^[^ ^82^ ^]^ accounting for baseline Δ*F*/*F* variations between different mice or within the same mouse across pre‐ and postintervention recordings. To eliminate variability introduced by this factor during comparisons, the mean signal value before each experiment's tail suspension was used as the baseline for normalizing the overall signal from that session (including signals before and during tail suspension). Unless otherwise specified, data were presented as mean ± standard error, with shaded areas in line graphs and error bars in statistical plots representing standard errors of the data. Statistical significance was determined using nonpaired *t*‐tests, denoted as *p < 0.05, **p < 0.01, ***p < 0.001, ns for no significance (*p* > 0.05).

### Single‐Nucleus Sequencing

Cell capture and complementary DNA (cDNA) synthesis were performed using the Single Cell 3′Library and Gel Bead Kit V3.1 (10x Genomics, 1000121) and Chromium Single Cell G Chip Kit (10x Genomics, 1000120). The nucleus suspension (300–600 mL^−1^) was loaded onto a chromium single‐cell controller (10x Genomics) to generate single‐cell gel beads in the emulsion, following the manufacturer's protocol. The captured nucleus was then lysed and the released RNA was barcoded through reverse transcription in individual Gel Beads‐in‐emulsion (GEMs). Reverse transcription was performed using an S1000TM Touch Thermal Cycler (Bio‐Rad) at 53 °C for 45 min, followed by 85 °C for 5 min, and holding at 4 °C. The cDNA was generated and then amplified. Quality was assessed using an Agilent 4200 system.

Single‐nucleus RNA‐seq libraries were constructed using the Single Cell 3′ Library and Gel Bead Kit V3.1. The libraries were sequenced using an Illumina Novaseq6000 sequencer with a sequencing depth of at least 100 000 reads per cell with a pair‐end 150 bp strategy.

### Single‐Nucleus Data Processing

Alignment, filtering, barcode counting, and unique molecular identifier (UMI) counting were performed using the CellRanger count module to generate feature‐barcode matrix and determine clusters. Dimensionality reduction was performed using principal component analysis (PCA) and the first ten principal components were used to generate clusters by the *K*‐means algorithm and graph‐based algorithm.

Cells with a gene number < 200, a gene number ranking in the top 1%, or a mitochondrial gene ratio > 25% were regarded as abnormal and filtered out. Dimensionality reduction was performed using PCA and visualization was performed using t‐distributed stochastic neighbor embedding (TSNE) and uniform manifold approximation and projection (UMAP).

GO enrichment, KEGG enrichment, and Reactome enrichment of cluster markers were performed using KOBAS software with Benjamini–Hochberg adjustment for multiple comparisons, using the top 20 marker genes of the cluster. The results were visualized using the R package.

Gene set enrichment analysis (GSEA) was performed using GSEA software version 2.2.2.4, which used predefined gene sets from the Molecular Signatures Database (MSigDB v6.2). All genes detected in all cells of one sample were used. Gene expression data were calculated using the mean UMI count of genes in one cluster and the remaining clusters. The minimum and maximum criteria for the selection of gene sets from the collection were 0 and 500 genes, respectively.

Single‐cell trajectories were built using Monocle (R package) to introduce pseudotime. Genes were filtered by the following criteria: expressed in >10 cells; an average expression value > 0.1; and a *Q* value < 0.01 in the differential analysis.

### Statistical Analysis

Data were presented as the mean ± standard error of the mean (SEM), unless otherwise specified. Statistical analyses were performed using GraphPad Prism 7 (GraphPad Software, Inc., USA). Student's *t*‐test was used for statistical comparisons between two groups. For comparisons between three or more groups, one‐way or two‐way ANOVA followed by Bonferroni post hoc tests was used. The level of statistical significance was *p* < 0.05, unless specified otherwise. No data were excluded from the analyses. All investigators were blinded to group allocation during data collection and analysis.

## Conflict of Interest

The authors declare no conflict of interest.

## Author Contributions

Y.K., X.Z., L.L., and T.Z. contributed equally to this work. Y.K. and Z.Z. conceived the study, supervised the experiments, and prepared a draft of the paper and revision. X.Z., L.L., and T.Z. jointly undertook and completed most experiments. Z.H. performed the experiment assessing whole‐brain expression of VDBP (Figure 1). M.L., Y.H., and L.Y. assisted constructed and characterized the gene knockout mice at the Shenzhen lab. A.Z. and Y.S. assisted in constructing and evaluating the model animals at the Nanjing lab. J.J. and G.Z. assisted with the preliminary in vitro and in vivo experiments. A.Z. assisted in the chart production and visualization under the guidance of Y.K. and Z.Z.

## Supporting information



Supporting Information

## Data Availability

All data supporting the findings of this study are available within the paper and its Supplementary Information. The snRNA‐seq data generated in this study has been deposited in Sequence Read Archive (SRA) (PRJNA1086271). Any information required to reanalyze the data reported in this paper is available from the lead contact upon request.
