# Supplementary material for: Microglia‐Derived Vitamin D Binding Protein Mediates Synaptic Damage and Induces Depression by Binding to the Neuronal Receptor Megalin
Source: Adv Sci (Weinh). 2024 Dec 23;12(6):2410273. doi: 10.1002/advs.202410273 (PMC11809382; doi:10.1002/advs.202410273)
Supplement: Supplementary file 1 — Supporting Information [file ADVS-12-2410273-s001.docx]

Supporting Information

**Microglia-derived Vitamin D Binding Protein Mediates Synaptic Damage and Induces Depression by Binding to the Neuronal Receptor Megalin**

*Yan Kong*, Xian Zhang, Ling Li, Te Zhao, Zihan Huang, Aini Zhang, Yun Sun, Jiao Jiao, Gaojia Zhang, Mengyu Liu, Yijun Han, Linfeng Yang, Zhijun Zhang**

**Supplementary Materials and Methods**

**Animals**

All mice were maintained at the specific-pathogen-free (SPF) animal facility of Southeast University/Shenzhen Institute of Advanced Technology, Chinese Academy of Sciences with a 12 h/12 h light/dark cycle, temperature of 23–25 ℃, and free access to food and water for more than 5 days. All experiments were conducted according to protocols approved both by the Institutional Animal Care and Use Committee of Southeast University (2020100106) and Shenzhen Institute of Advanced Technology. Male mice were used for this study to exclude potential effects of the female estrous cycle. Our sample sizes are similar to those reported in previous publications (PMID: 31451801; PMID: 31851918; PMID: 31948733). The animals in the behavioral tests were randomly assigned by the function Rand in the spreadsheet software Excel.

All mice of C57BL/6J background were purchased from GemPharmatech (Nanjing, China). Cx3cr1-Cre^ERT2^ mice were obtained from Jackson Laboratory (Bar Harbor, ME, USA).

**Cell culture**

Mouse microglia BV2 cells were purchased from the China Center for Type Culture Collection (GDC0311, CCTCC, Wuhan, China). BV-2 is a type of microglial cell derived from C57/BL6 murine. The BV2 cells are immortalized by v-raf/v-myc carrying J2 retrovirus. BV2 expresses nuclear v-myc and the cytoplasmic v-raf oncogene products as well as the env gp70 antigen at the surface level. BV2 microglia cell line retains microglia morphological and functional characteristics. BV2 cells were cultured with DMEM (Gibco) supplemented with 10% FBS (Gibco) and 100 U/mL penicillin/streptomycin in an incubator (Thermo Scientific) with 5% CO_2_ at 37℃.

**Primary neuron culture**

Primary neurons were dissected from embryonic mice (E16.5). Briefly, timed-pregnant female mice were sacrificed, the pups were extracted, and the pups’ brains were dissected. After removing the meninges, the cortical tissues were isolated and digested with 0.25% trypsin-EDTA (GIBCO). Primary neurons were plated on poly-D-lysine coated dishes and cultured with neurobasal medium supplemented with B27 (GIBCO).

**Primary microglia and astrocyte culture**

Primary astrocytes and microglia were isolated from newborn mouse pups (postnatal days 8–10). Brains were dissected and digested in trypsin-DNase solution (2.5% trypsin and 10 mg/mL DNaseI). After filtration using a 40 μm strainer and centrifugation, the cell pellets were suspended in the microglia culture medium (DMEM, 10% FBS, 10 mM non-essential amino acids, 40U insulin) and seeded on flasks. The medium was changed every 3 days. After 10 days, the flasks were shaken at 260 rpm for 12 h. The supernatant was then collected and transferred to new dishes. After incubation for 1 h, the medium was removed and microglia cells were seeded on the dishes. The remaining astrocytes on the original flasks were digested and transferred to new dishes in the astrocyte medium (DMEM/F12, 10% FBS) for further experiments.

**Plasmid, siRNA, and shRNA**

The plasmid pcDNA3.1 (Thermo Fisher Scientific) was used to construct the mouse VDBP (Gene ID: 14473; NCBI Reference Sequence: NM_008096.2) overexpression plasmid. siRNAs targeting VDBP were designed and synthesized by GenePharma (Shanghai, China). The RNA oligo sequences are listed in Table S2. The shRNA targeting mouse megalin (low-density lipoprotein receptor-related protein 2, LRP2) was based on a previously published validated siRNA sequence (sense GCUAUUGUAUUAGAUCCUUtt and antisense AAGGAUCUAAUACAAUAGCtc) ^[58]^.

**VDBP siRNA transfection**

VDBP siRNA and control RNA oligos (GenPahrma, Shanghai, China) were transfected into BV2 cells and primary microglia using Lipofectamine 2000 (Thermo Fisher, USA) according to the manufacturer’s instructions. Briefly, BV2 cells or primary microglia were seeded in 6-well plates at a density of 5×10^3^ cells/mL one day before transfection. Next, 5 μL Lipofectamine 2000 was added to Opti-MEM medium without serum and incubated for 5 min. VDBP siRNA or control RNA oligos were diluted in the same medium to a final concentration of 400 nM. The medium containing Lipofectamine 2000 and RNA oligos was then mixed and incubated for 20 min before being added to BV2 cells or primary microglia. After 48 h, the transfected cells were harvested and the expression of VDBP at the mRNA and protein levels was texted.

**Transmission electron microscopy**

After treatment, adherent neurons were depleted using trypsin and centrifuged. The cell pellet was fixed with precooled 2.5% glutaraldehyde solution in PBS at 4 °C for 2 h, followed by staining with 1% osmium tetroxide for 30 min. The cells were then dehydrated using a gradient of 30%, 50%, 70%, 80%, 95%, and 100% ethanol. The cells were embedded and ultrathin sections were stained with saturated uranyl acetate and lead citrate before observation under an electron microscope (HT7800, HITACHI, Japan).

**Proximity ligation assay (PLA)**

Primary mouse neurons on coverslips were fixed with 4% paraformaldehyde and permeabilized with 0.2% Triton X-100 before incubation with the blocking solution in a preheated humidity chamber. Neurons were further incubated with primary rabbit anti-VDBP (16922-1-AP, Proteintech) at 1:500 and mouse anti-megalin/ LRP2 (sc-515772, Santa Cruz) at 1:400, followed by addition of the Duolink In Situ PLA anti-rabbit PLUS probe (DUO9002, Sigma-Olink) and the anti-mouse MINUS probe (DUO92004, Sigma-Olink). The PLA signal was detected using the Duolink in situ PLA detection kit (DUO92008, Sigma-Olink). Nuclei were labeled using DAPI. Positive PLA signals were visualized as red dots. Neurons were also immunostained with MAP2 antibody. Negative control data showing no PLA signal without the VDBP primary antibody are shown in Extended data Fig.5b.

**Cell viability test**

The CCK-8 kit (Beyotime, Shanghai, China) was used to determine the viability of primary neurons. Mouse cortical neurons were seeded at a density of 5×10^3^ on 96-well plates. After treatment with conditional medium from DX-treated BV2 cells or primary microglia, 10 μL CCK-8 reagent was added to each well and incubated at 37 °C with 5%CO_2_ for 1 h. The cells were then read at 450 nm using a plate reader (Bio-Rad, Hercules, CA, USA). Finally, cell viability was determined according to the kit manufacturer’s instructions.

**TUNEL assay**

TUNEL assay was performed using the Click-iT™ Plus TUNEL Assay Kit according to the manufacturer’s instructions. Briefly, brain slices or cultured primary neurons were fixed with 4% paraformaldehyde and permeabilized in 0.25% Triton™ X-100. After incubation with the TdT Reaction mixture and the Click-iT™ Plus TUNEL reaction cocktail, the samples were stained with DAPI to visualize the nucleus. Finally, the samples were mounted and visualized using an Olympus FV3000 confocal microscope.

**Analysis of dendritic morphology**

Neuronal dendritic morphology was analyzed by immunostaining with microtubule-associated protein 2 (MAP2). To analyze the dendritic structure of neurons, images were semiautomatically traced and the lengths and branching points of dendrites were measured using Fiji software (Win64). Sholl analysis for dendritic complexity was carried out by counting the number of dendrites crossing a series of concentric circles at 7.5 μm intervals from the soma.

**Quantitative real-time PCR (qPCR)**

Total RNA was extracted from culture cells or animal brain tissues using TRIzol reagent (15596026, Invitrogen) according to the manufacturer’s instructions. Reverse transcription was performed using the HiScript III 1st Strand cDNA Synthesis Kit (+gDNA wiper) (R312-01, Vazyme) followed by real-time PCR using ChamQ SYBR qPCR Master Mix (Q341-02, Vazyme) and a ABI 7300 Real-Time PCR System. The 2-ΔΔCt method was used to calculate relative gene expression with β-actin as an internal control. The primer sequences used for qPCR are listed in Table S1.

**Western blotting**

Cultured cells and brain tissue samples were homogenized in RIPA lysis buffer. After centrifugation at 1,2000 x *g* for 10 min, the supernatants were boiled, separated by sodium dodecyl-sulfate polyacrylamide gel electrophoresis (SDS-PAGE), and transferred to PVDF membranes (Immobilon-P, Millipore). The membranes were blocked with 5% nonfat dry milk before incubation overnight at 4 ℃ with primary antibodies, including VDBP (16922-1-AP, Proteintech), β-actin (81115-1-RR, Proteintech), PSD95 (ab18258, Abcam), Synapsin Ⅰ (ab64581, Abcam), SNAP25 (ab109105, Abcam), VGAT (4471-1-AP, Proteintech), VGLUT (ab227805, Abcam), Phospho-Src (Tyr419) (AF3162, Affinity), Phospho-ERK1/2 (Thr202/Tyr204) (AF1015, Affinity), Phospho-AKT1/2/3 (Ser473) (AF0016, Affinity), Phospho-CREB (Ser133) (AF3189, Affinity), Bax (AF0120, Affinity), Bcl-2 (AF6139, Affinity), and GAPDH (AF7021, Affinity). After further incubation with goat-anti-mouse or goat-anti-rabbit secondary antibodies and reaction with SuperSignal™ West Pico PLUS Chemiluminescent Substrate (Thermo Scientific), the bands were visualized using a Tanon 3500 Gel Imaging System. The intensities of the protein bands were normalized to β-actin and calculated from at least three independent experiments.

***In vitro* brain slice electrophysiology**

**Brain slice preparation**

Mice were deeply anesthetized by intraperitoneal injection of urethane (25%, 1 mL/100 g) and then decapitated. The brains were rapidly extracted from the skull using the least amount of pressure to minimize damage to brain tissue. Coronal slices (280 µm) containing the mPFC were made using a Vibratome VT1200S (Leica, Germany). The mPFC slices were then processed in ice-cold sucrose a cerebrospinal fluid (aCSF) containing (in mM) 185 sucrose, 2.5 KCl, 1.2 NaH_2_PO_4_, 25 NaHCO_3_, 25 D-glucose, 0.5 CaCl_2_, and 10 MgSO_4_. The temperature of the slicing chamber was maintained at 0−4 ℃ throughout the slicing process. The prepared slices were recovered in normal aCSF (in mM: 125 NaCl, 2.5 KCl, 1.25 NaH_2_PO_4_, 25 NaHCO_3_, 10 D-Glucose, 2.0 CaCl_2_, and 1.5 MgCl_2_) at 32℃ for 30 min, then incubated at RT for a minimum of 1 h. All aCSF was saturated with carbogen (95%O_2_/5%CO_2_).

**Brain slice electrophysiology setup**

Electrophysiological recordings were obtained under visual control using an upright microscope (BX50-WI, Olympus, Japan) equipped with 10×, 5×, and 40× ocular objectives. An infrared phase contrast CCD camera (Retiga ELECTRO, Qimaging, Canada) in the microscope made it possible to visualize neurons within brain slices. The brain slices were transferred to a “submerged” recording chamber continuously perfused with aCSF using a peristaltic pump (BT100-2J, LongerPump, China). Throughout the procedure, brain slices were maintained on a nylon net in a fixed position and covered by short silver wire loads. The recording chamber and microscope were installed on an anti-vibration table (PM-H-10-08-L, Ruixuguangdian, China) and surrounded by a Faraday cage. Outside the Faraday cage, aCSF was continuously saturated with carbogen (95%O_2_/5%CO_2_) in a thermostat water bath (HH-501, Xinbao Equipment. China).

Recordings were acquired using an Axopatch 700B amplifier and Digidata 1440A (Molecular Devices, USA) with pClamp10.7 software. Recording pipettes were pulled from thin wall borosilicate glass with filament (BF150-110-10, Sutter Instrument, USA). The pipettes were produced with electronic electrode-extracting equipment (P97, Sutter Instrument, USA). The recording electrode was controlled by a motorized micromanipulator (MP225, Sutter Instrument, USA), and the stimulating electrode was controlled by a mechanical micromanipulator (CFT-8301B, Jiangsuruiqi, China). Square-wave current pulses were delivered through a stimulus isolator (Isoflex, AMPI, Israel).

**Whole-cell patch clamp recordings**

After incubation for 1 h in normal aCSF at RT, the slices were placed in a recording chamber. Whole-cell recording was performed for L5 pyramidal neurons located in the PrL region, which were identified based on their position and morphology.

For mEPSCs, glass pipettes (input resistance 2−5 MΩ) were filled with an intracellular solution containing (in mM):140 K-Gluconate, 2 MgCl_2_, 10 HEPES, 8 KCl, 2 Na2-ATP, and 0.2 Na_2_-GTP (pH 7.3). To isolate mEPSCs, 1 µM tetrodotoxin (TTX) was added to the bath solution (normal aCSF: 125 NaCl, 2.5 KCl, 1.25 NaH_2_PO_4_, 25 NaHCO_3_, 10 D-Glucose, 2.0 CaCl_2_, and 1.5 MgCl_2_) together with the GABAA receptor blocker bicuculline methiodide (10 µM). Recordings were performed with a voltage clamp at a holding potential of −70 mV.

For mIPSCs, glass pipettes (input resistance 2−5 MΩ) were filled with an intracellular solution containing (in mM): 140 CsCl, 0.1 CaCl_2_, 2 MgCl_2_, 1.1 EGTA, 2 Na2-ATP, and 10 HEPES (pH 7.2−7.3). To isolate mIPSCs, 1 µM tetrodotoxin (TTX), 20 μM CNQX, and 20 μM DAP5 were added to the bath solution (normal aCSF: 125 NaCl, 2.5 KCl, 1.25 NaH_2_PO_4_, 25 NaHCO_3_, 10 D-Glucose, 2.0 CaCl_2_, and 1.5 MgCl_2_) together with the GABAA receptor blocker bicuculline methiodide (10 µM). Recordings were performed with a voltage clamp at a holding potential of −70 mV.

For sEPSCs, glass pipettes (input resistance 2−5 MΩ) were filled with an intracellular solution containing (in mM): 140 K-Gluconate, 2 MgCl_2_, 10 HEPES, 8 KCl, 2 Na_2_-ATP, 0.2 Na2-GTP, and 2 QX-314 (pH 7.3). Neurons were maintained at -70 mV and GABAA receptors were blocked using 10 µM bicuculline methiodide. For sIPSC recordings, the holding potential was -70 mV. 10 µM CNQX and 20 µM DAP5 were added to the bath solution. The pipettes (input resistance 2−5 MΩ) were filled with an intracellular solution containing (in mM) 140 CsCl, 0.1 CaCl_2_, 2 MgCl_2_, 10 HEPES, 1.1 EGTA, 2 Na_2_ATP, and 2 QX-314 (pH 7.3).

After the currents stabilized for approximately 2–3 min, a fixed duration of traces (5 min) was analyzed for frequency and amplitude distributions. Whole-cell currents were digitized at 20 kHz and filtered at 2 kHz. For all experiments, the series resistance was usually < 25 MΩ and fluctuated within 30% of the initial values. Resistance was monitored before and after recording to ensure constancy. Acquisition and analysis of current signals were performed using a MultiClamp 700B amplifier, 1440A digitizer, and pClamp 10.7 software (Axon Instruments).

**Experimental design**

1. **Whole-brain analysis of VDBP expression in wild-type, CUMS-susceptible, and CUMS-resilient mice**

C56BL6/J mice were stressed with CUMS and whole brain expression of VDBP was measured by immunohistochemistry of control, susceptible and resilient mice. Upregulation of VDBP gene expression was also confirmed in the postmortem prefrontal cortex of MDD patients by analyzing GEO datasets. Then nerve cell type specificity of VDBP upregulation was clarified at mRNA and protein level.

1. **VDBP was upregulated in DX-stressed MG and led to neuron damage *in vitro***

Primary mouse MG and BV2 cells were stressed with dexamethasone (DX), after which VDBP expression was measured at mRNA, protein, and secretion levels. Neuron damages caused by DX-stressed or VDBP-overexpressed MG were detected. Conversely, siRNA targeting VDBP was used to test whether it could reverse the neurotoxic effects of conditioned medium from DX stressed-MG.

1. **Effects of specific overexpression of MG-derived VDBP in the PrL region on depressive-like behaviors and susceptibility to CUMS in mice**

After being overexpressed in MG of PrL via AAV infection, the effects of VDBP on depressive-like behaviors and susceptibility to CUMS were tested. The results were validated by direct injection of VDBP protein into mouse PrL. Furthermore, VDBP overexpression on CUMS induced neuron damages were tested by synaptic proteins, Golgi staining, and electrophysiologic techniques.

1. **Resilience to CUMS-induced depressive-like behavior in mice with MG VDBP-conditioned knockout mice**

CRISPR/Cas9 was used to construct VDBP^fl/fl^ mice which were further crossed to obtain Cx3cr1-Cre^ERT2^; VDBP^fl/fl^ mice and injected with tamoxifen to obtain MG-specific KO-VDBP mice. The resilience of KO-VDBP mice to CUMS was measured. Furthermore, VDBP KO on rescued neuron damages was tested by synaptic proteins, Golgi staining, and electrophysiologic techniques.

1. **MG-derived VDBP binds to the neuronal receptor megalin and regulates SRC downstream signaling pathways, leading to neuronal synaptic damage and depressive-like behaviors.**

The interaction of VDBP with megalin on neurons was validated by GST-pull down, PLA, and RAP blocking in uptake assay. The effects of conditioned medium from DX-treated MG on the SRC pathway and cell death of neurons were blocked by RAP. Accordingly, megalin was knocked down by shRNA in PrL neurons of VDBP overexpressed mice and the amelioration of depressive-like behaviors, neuron damages, and electrophysiological functions were measured.

1. **Specificity of MG-derived VDBP action on neuronal subtypes related to depression**

Single-nucleus sequencing was performed on PrL of VDBP overexpressed mice. Bioinformatic analysis was performed to identify GABAergic neurons as the major target. The findings were further validated by electrophysiological assay, miniaturized two-photon fluorescence microscope, and immunoconfocal experiments.

**Supplementary figure S1**


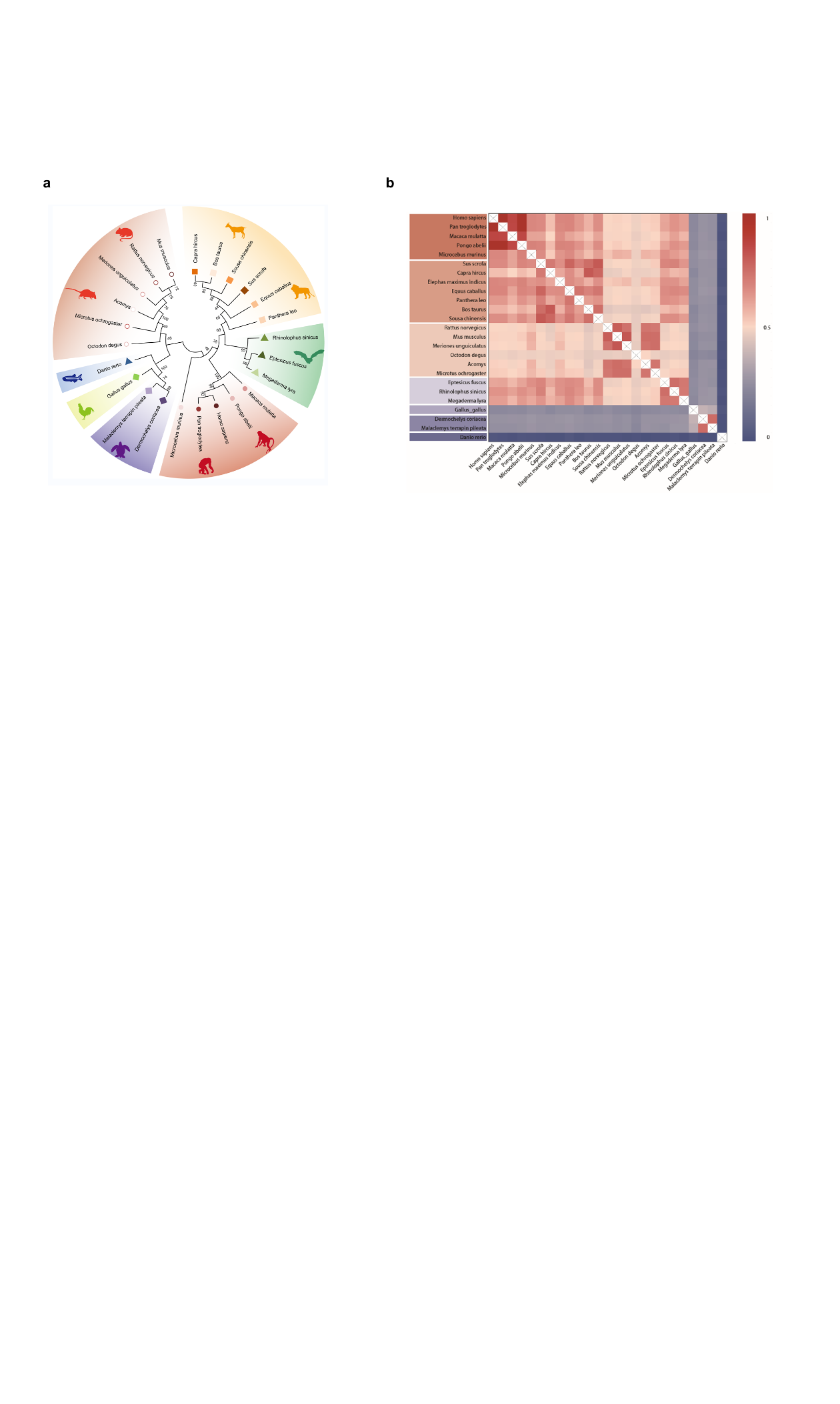
**Supplementary figure S1**. **Evolutionary phylogeny analysis of VDBP. a** The evolutionary history was inferred using the neighbor-joining method and the Jones-Taylor-Thornton (JTT) model. The target protein sequence was compared in the NCBI database using protein BLAST to obtain the protein sequence with high homology with VDBP (GC). Next, the downloaded protein sequence file was imported into MEGA 11 for alignment using Muscle. A discrete gamma distribution was used to model evolutionary rate differences among sites [8 threads (+G, parameter = 0.9112)]. **b** Estimates of evolutionary divergence between VDBP sequences (GC) in different species. The number of base substitutions per site between sequences is shown. Standard error estimate (s) are shown above the diagonal and were obtained by a bootstrap procedure (1000 replicates). The rate variation among sites was modeled with a gamma distribution (shape parameter = 1). The analysis indicated that VDBP is conserved in biological evolution.

**Supplementary figure S2**

**
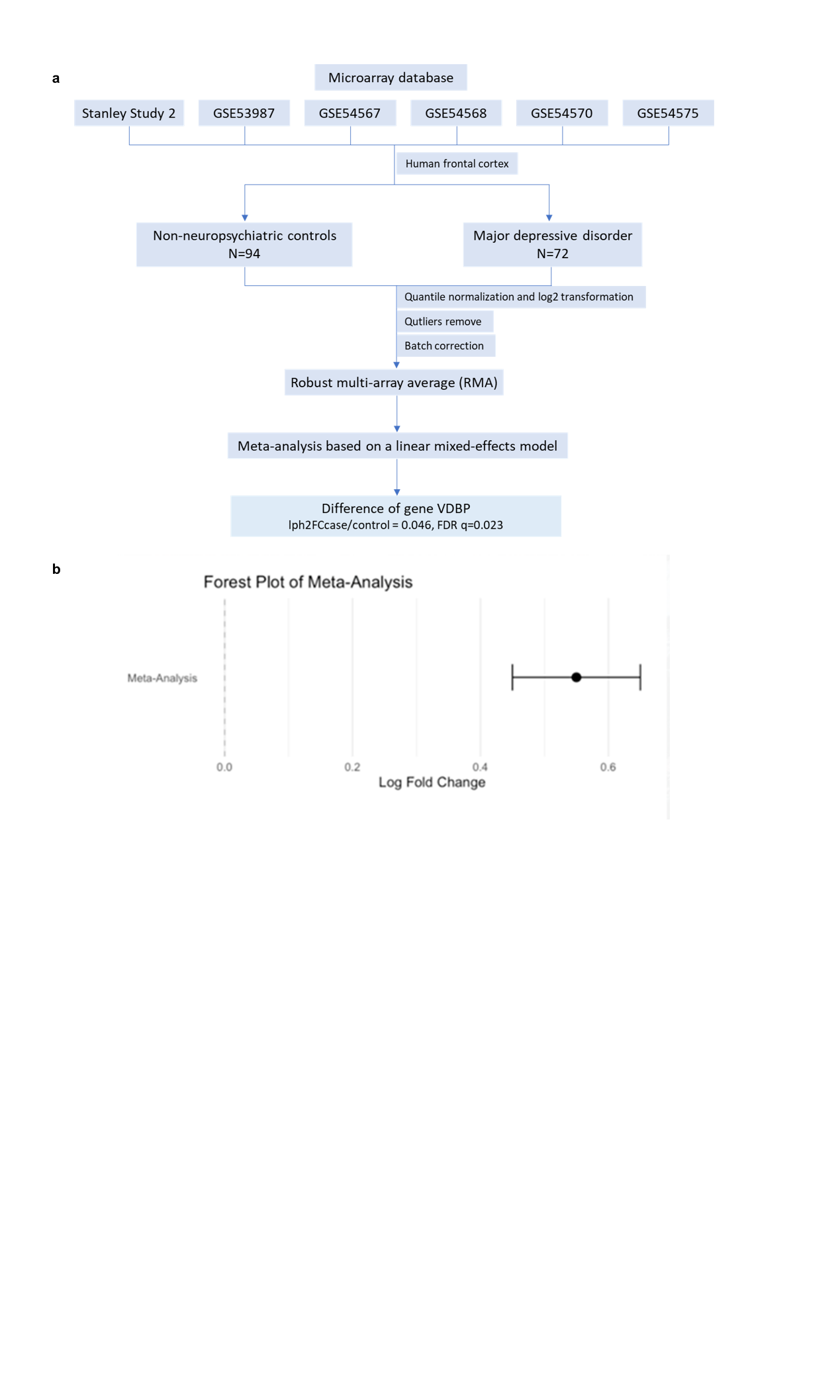
Supplementary figure S2. Meta-analysis of microarray datasets showed increased VDBP mRNA expression in the frontal cortex of postmortem MDD patients. a** Flowchart of the meta-analysis of VDBP expression in postmortem human frontal cortex samples from non-neuropsychiatric controls (n = 94) and MDD patients (n = 72). Microarray datasets of the Stanley study 2, GSE53987, GSE54568, GSE54570, and GSE54575. **b** A forest plot of the meta-analysis showed a significant difference in VDBP expression in MDD patients and the controls. logFC (Case/Control) = 0.046, FDR q = 0.023. The result showed the increased expression of the VDBP gene in PFC in MDD patients compared to the controls, supporting the role of increased brain-derived VDBP [in](file:///C:\Program%20Files%20(x86)\Youdao\Dict\7.2.0.0703\resultui\dict\?keyword=in) depression.

**Supplementary figure S3**


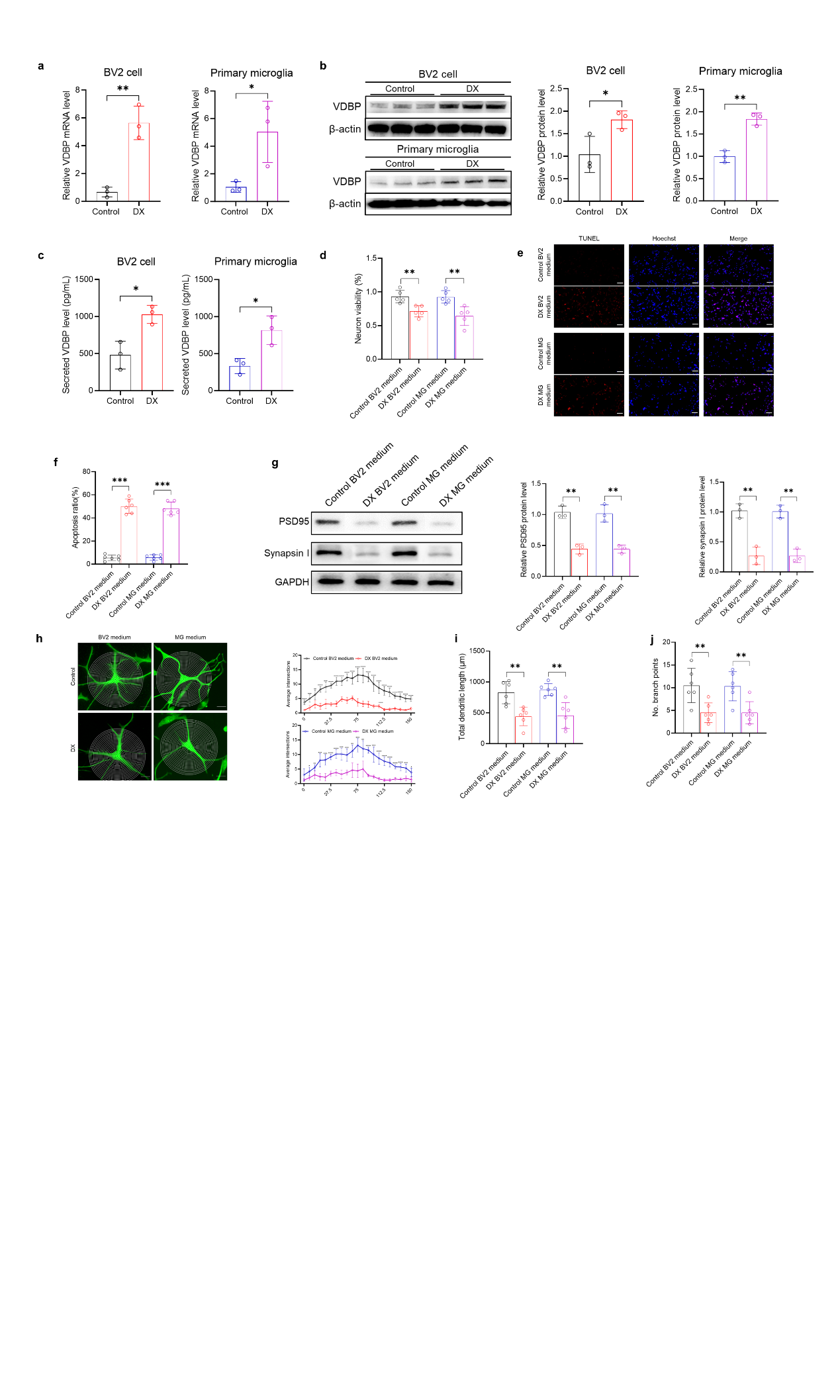
**Supplementary figure S3. Microglia derived-VDBP is increased by dexamethasone-induced stress *in vitro* and leads to impairments of neurons and synapses. a** RT-qPCR showed that VDBP mRNA was increased in DX-treated BV2 cells and primary mouse cortex microglia (MG). n = 3 biological replicates. **b** Western blotting with quantification showed VDBP protein levels were increased in DX-treated BV2 cells and MG. n = 3 biological replicates. **c** ELISA validated higher secreted VDBP protein levels in the medium of DX-treated BV2 cells and MG. n = 3 biological replicates. **d** CCK-8 assay showed the viability of primary cultured mouse cortical neurons exposed to conditioned medium from DX-treated BV2 cells and MG was decreased. Control BV2 medium: conditioned medium from normal BV2 cells. DX BV2 medium: conditioned medium from DX-treated BV2 cells. Control MG medium: conditioned medium from normal MG. DX MG medium: conditioned medium from DX-treated MG. TUNEL assay (**e)** and quantification (**f)** showed increased apoptotic neurons treated with different conditioned media from BV2 and MG. Nuclei of neurons stained with Hoechst 33342 are indicated in blue, while apoptotic neurons stained with TUNEL are indicated in red. Scale bar = 10 µm. n = 6 biological replicates. **g** Western blotting with quantification showed decreased synaptic protein PSD95 and synapsin Ⅰ of primary mouse cortical neurons treated with different conditioned BV2 and MG media. n = 3 biological replicates. **h-j** Primary mouse cortical neurons treated with different conditioned BV2 and MG media and immunostained with anti-MAP2 antibody. Decreased dendrite complexity **(h),** average total dendritic length **(i),** and branch number **(j)** of primary cultured cortical neurons (n = 6 cells per group) were observed. Scale bar = 30 µm. The presented data are the mean ± standard error of the mean (SEM). **p* < 0.05, ***p* < 0.01, and ****p* < 0.001.

**Supplementary figure S4**


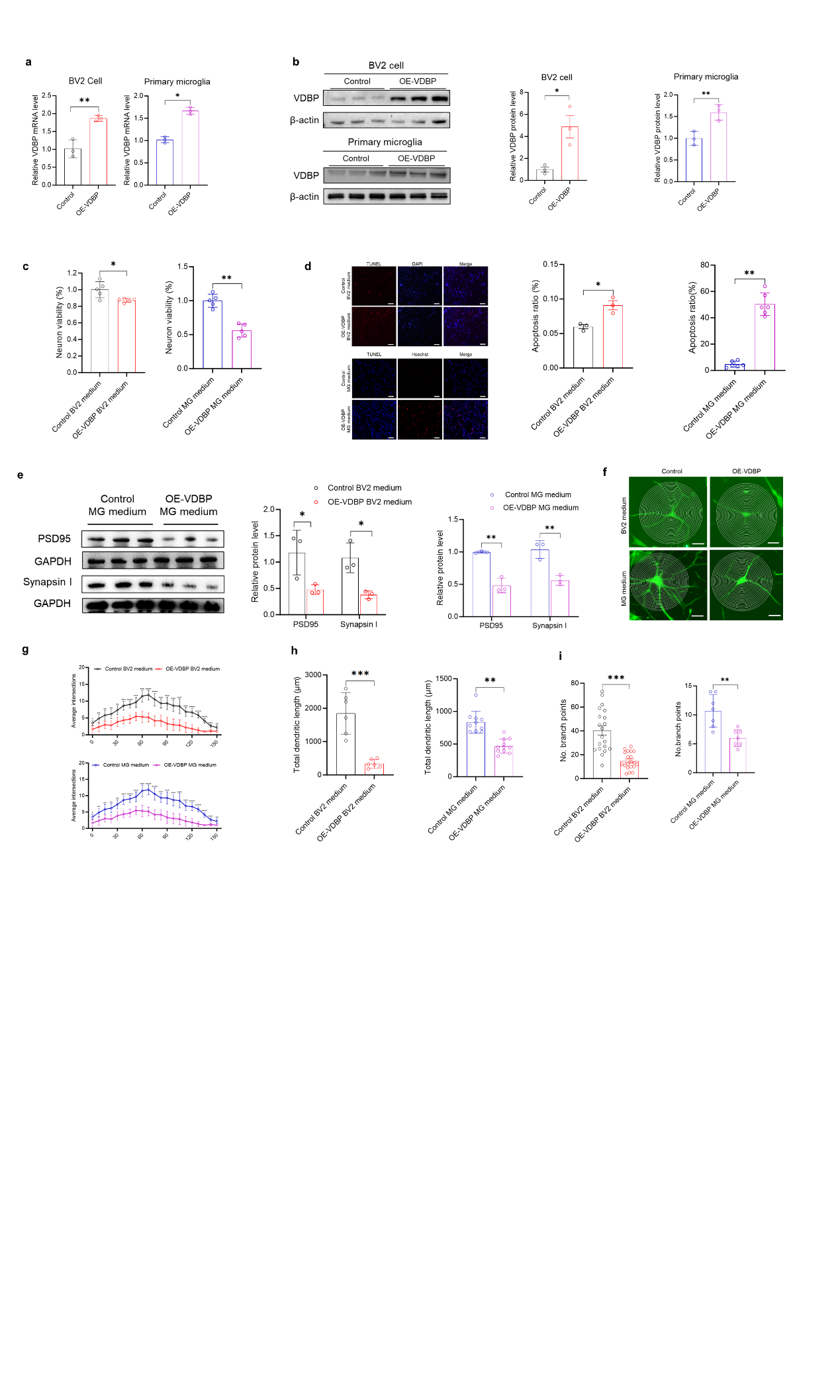
 **Supplementary figure S4. VDBP overexpression in MG leads to impairments of neurons and synapses.** **a** RT-qPCR of VDBP mRNA level in BV2 and MG transfected with the VDBP overexpression plasmid. Control: empty-vector transfected cells. OE-VDBP: VDBP overexpression plasmid transfected cells. n = 3 biological replicates. **b** Western blotting with quantification to validate the level of VDBP protein overexpression in BV2 and MG. n = 3 biological replicates. **c** CCK-8 assay of primary mouse cortical neurons exposed to conditioned media of empty vector-transfected (control), VDBP overexpression plasmid transfected (OE-VDBP) BV2 cells (BV2 medium), or MG (MG medium). n = 5 biological replicates. **d** TUNEL assay and quantification of apoptotic primary mouse cortical neurons exposed to conditioned media of empty vector-transfected (control), VDBP overexpression plasmid transfected (OE-VDBP) BV2 cells (BV2 medium), or MG (MG medium). Nuclei of neurons stained with DAPI are indicated in blue, while apoptotic neurons stained with TUNEL are indicated in red. Scale bar = 10 µm. Right: quantification of TUNEL assay (n = 3 biological replicates for conditioned medium from BV2 cells, n = 6 replicates for conditioned medium from MG). **e** Western blotting with quantification of synaptic protein levels of primary mouse cortical neurons treated with conditioned medium from empty vector-transfected (control) or VDBP overexpression plasmid transfected (OE-VDBP) BV2 cells (BV2 medium), or MG (MG medium). n = 3 biological replicates. **f** Primary mouse neurons were treated with conditioned media from the empty vector (control) or VDBP overexpression vector-transfected (OE-VDBP) BV2 cells (BV2 medium) or MG (MG medium) and immunostained with anti-MAP2 antibody. Representative confocal projection images of dendritic complexity are shown. Scale bar = 30 µm. Sholl analysis **(g)** (n = 8 cells per group) and quantification of average total dendritic length (n = 6 for BV2 medium, n=10 for MG medium) **(h)** and branch number ((n = 20 for BV2 medium, n=7 for MG medium)) **(i)** of primary cultured cortical neurons. The presented data are the mean ± standard error of the mean (SEM). **p* < 0.05, ***p* < 0.01, and ****p* < 0.001.

**Supplementary figure S5**

**
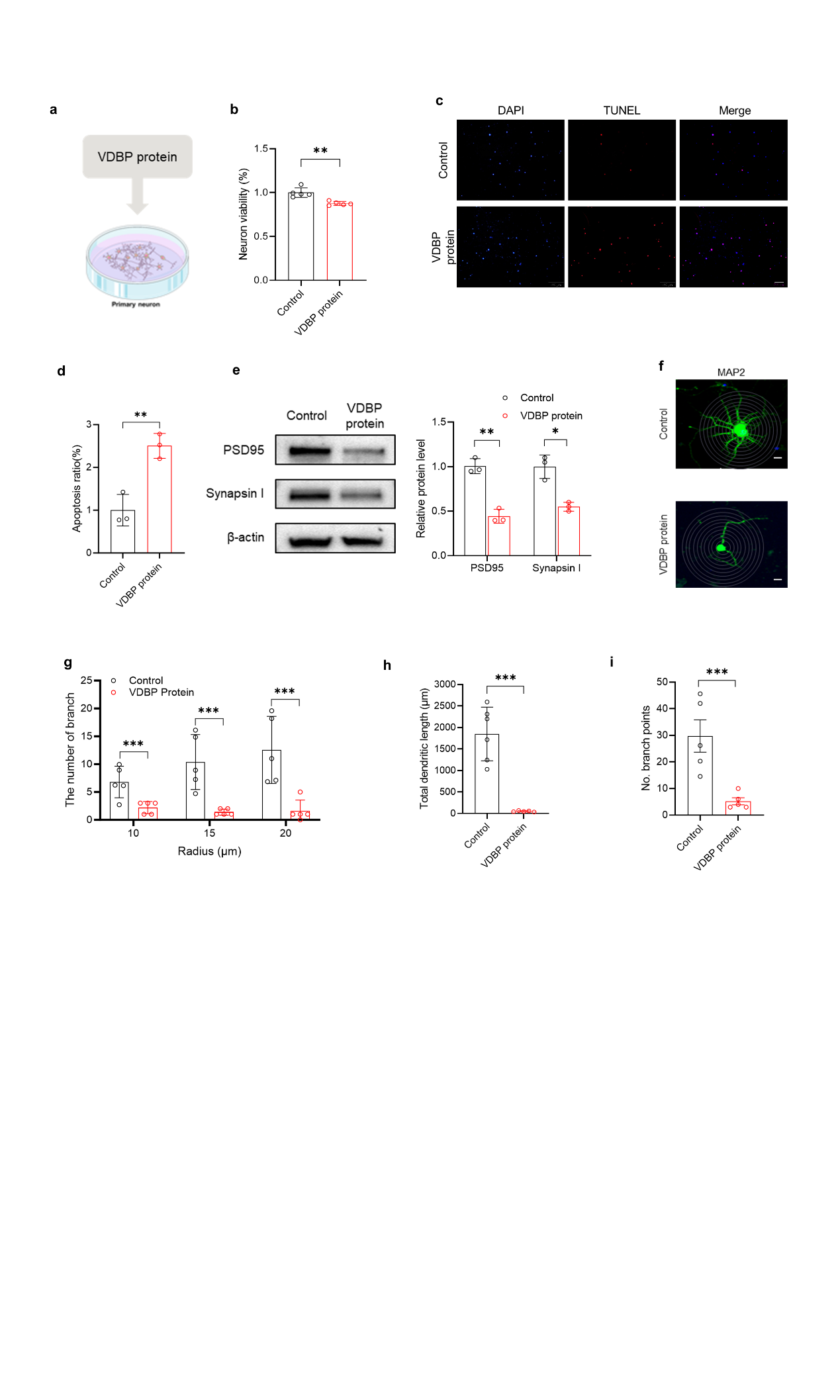
Supplementary figure S5. High doses of VDBP protein induce neuronal and synaptic damage**. **a** Schematic diagram showing the workflow of primary mouse cortical neurons treated with recombinant VDBP protein. **b** CCK-8 assay of primary cultured mouse cortical neurons exposed to VDBP protein. n= 5 biological repeats. **c, d** TUNEL assay of primary cultured mouse cortical neurons exposed to VDBP protein. n= 3 biological repeats. **e** Western blotting with quantification of synaptic protein levels of primary mouse cortical neurons treated with VDBP protein. n = 3 biological replicates. **f-i** Primary mouse cortical neurons were treated with VDBP protein and immunostained with anti-MAP2 antibody. Sholl analysis **(g)** and quantification of average total dendritic length **(h)** and branch number **(i)** of primary cultured cortical neurons (n = 6 cells per group). Data were analyzed using the two-tailed unpaired t-test and expressed as the mean ± SEM. **p* < 0.05, ***p* < 0.01, ****p* < 0.001.

**Supplementary figure S6**


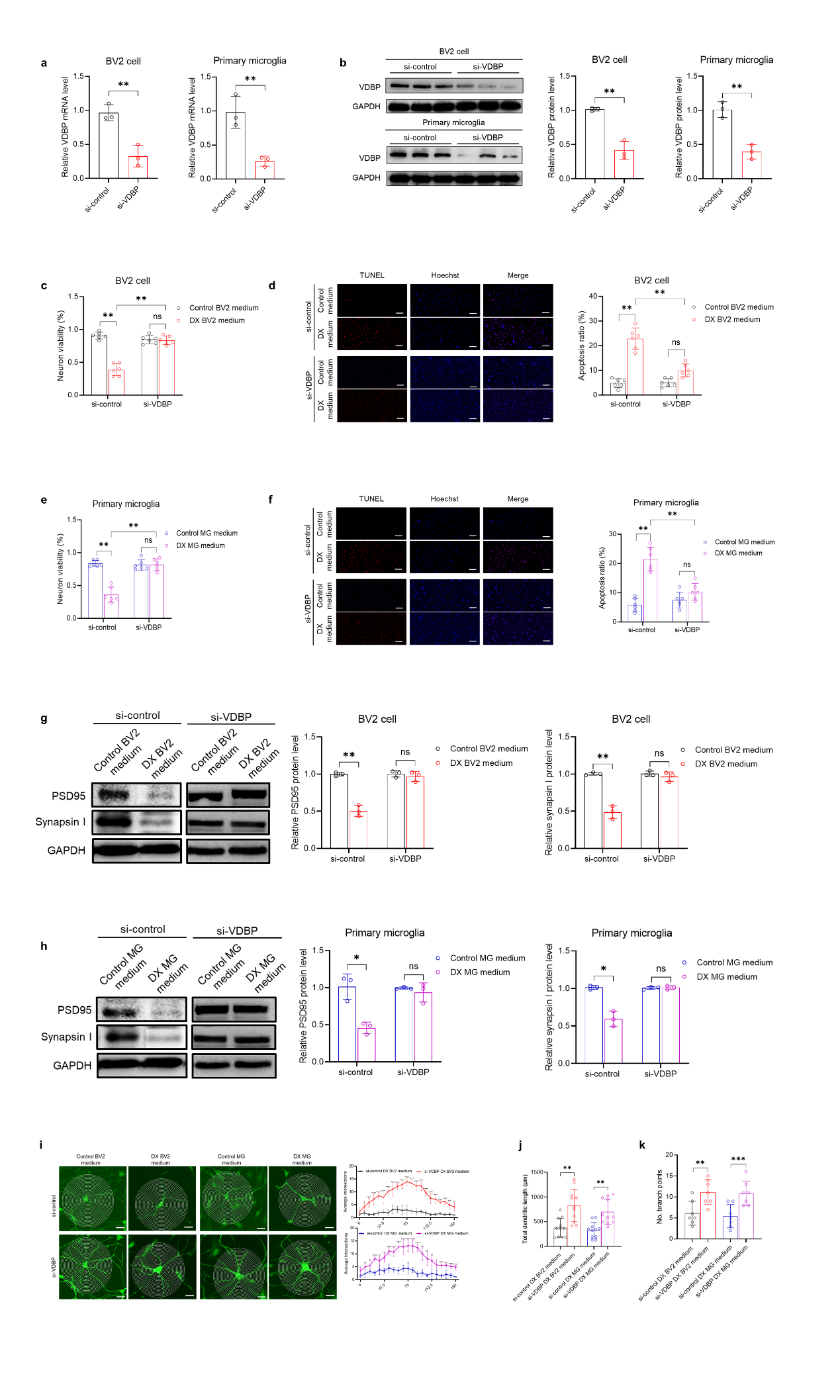
**Supplementary figure S6. Knockdown of VDBP in BV2 or primary microglia reverses impaired neurons and synapses induced by dexamethasone-stressed microglia conditioned medium**

**. a** RT-qPCR of VDBP mRNA level in siRNA transfected BV2 and MG cells. si-control: random RNA oligos transfected cells. si-VDBP: siRNA targeting VDBP transfected cells. n = 3 biological replicates. **b** Western blotting validation of the protein level of VDBP in siRNA transfected BV2 and MG cells. n =3 biological replicates. **c** CCK-8 assay of primary mouse cortical neurons exposed to conditioned medium of BV2 cells. Control BV2 medium: conditioned medium from normal BV2 cells. DX BV2 medium: conditioned medium from DX-treated BV2 cells. n =6 biological replicates. **d** TUNEL assay of apoptotic primary mouse cortical neurons exposed to conditioned medium from BV2 cells transfected with siRNA and treated with DX. n = 6 biological replicates**. e** CCK-8 assay of primary mouse cortical neurons exposed to conditioned medium of MG. Control MG medium: conditioned medium from normal MG. DX BV2 medium: conditioned medium from DX-treated MG. n = 6 biological replicates**. f** TUNEL assay of apoptotic primary mouse cortical neurons exposed to conditioned medium from MG transfected with siRNA and treated with DX. n = 6 biological replicates**.** Scale bar = 10 µm. **g** Western blotting with quantification of synaptic protein levels of primary mouse cortical neurons exposed to conditioned medium from BV2 cells transfected with VDBP siRNA and treated with DX. n = 3 biological replicates per group. **h** Western blotting with quantification of synaptic protein levels of primary mouse cortical neurons exposed to conditioned medium from MG cells transfected with VDBP siRNA and treated with DX. n = 3 biological replicates per group. **i-k** Primary mouse neurons exposed to conditioned medium from BV2 and MG were transfected with siRNA and treated with DX. Representative confocal projection images of dendritic complexity are shown. Scale bar = 20 mm. Sholl analysis **(i)** (n = 8) and quantification of average total dendritic length **(j)** (n = 11) and branch number **(k)** (n = 7) of primary cultured cortical neurons. The presented data are the mean ± SEM**.** **p* < 0.05, ***p* < 0.01, ****p* < 0.001.

**Supplementary figure S7**


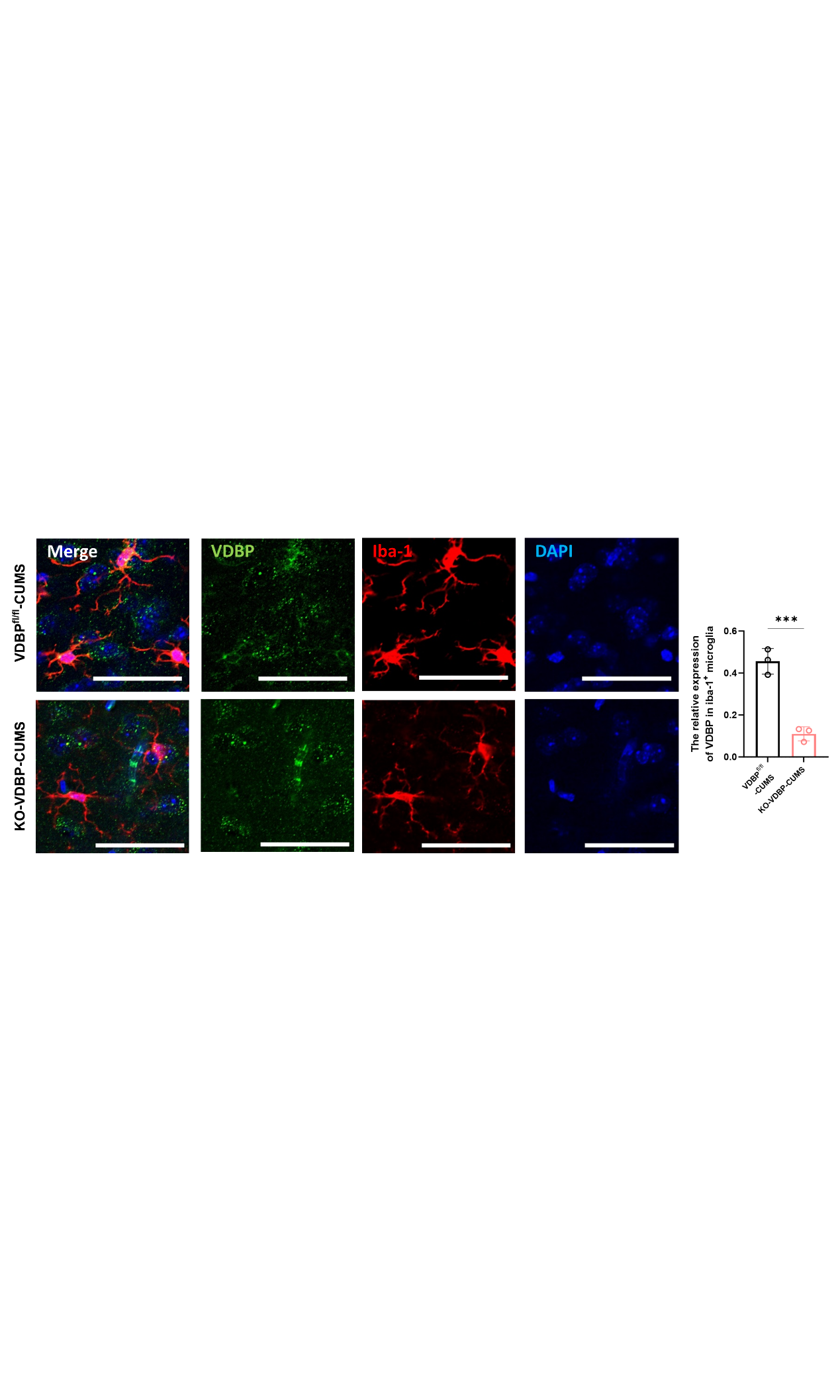
**Supplementary figure S7. Immunofluorescence and statistics of VDBP in microglia of the PrL from VDBP^fl/fl^-CUMS and KO-VDBP-CUMS mice.** Blue: DAPI. Green: VDBP. Red: Iba1as microglia marker. Scale bar = 50 μm. n = 3 mice. ****p* < 0.001.

**Supplementary figure S8**


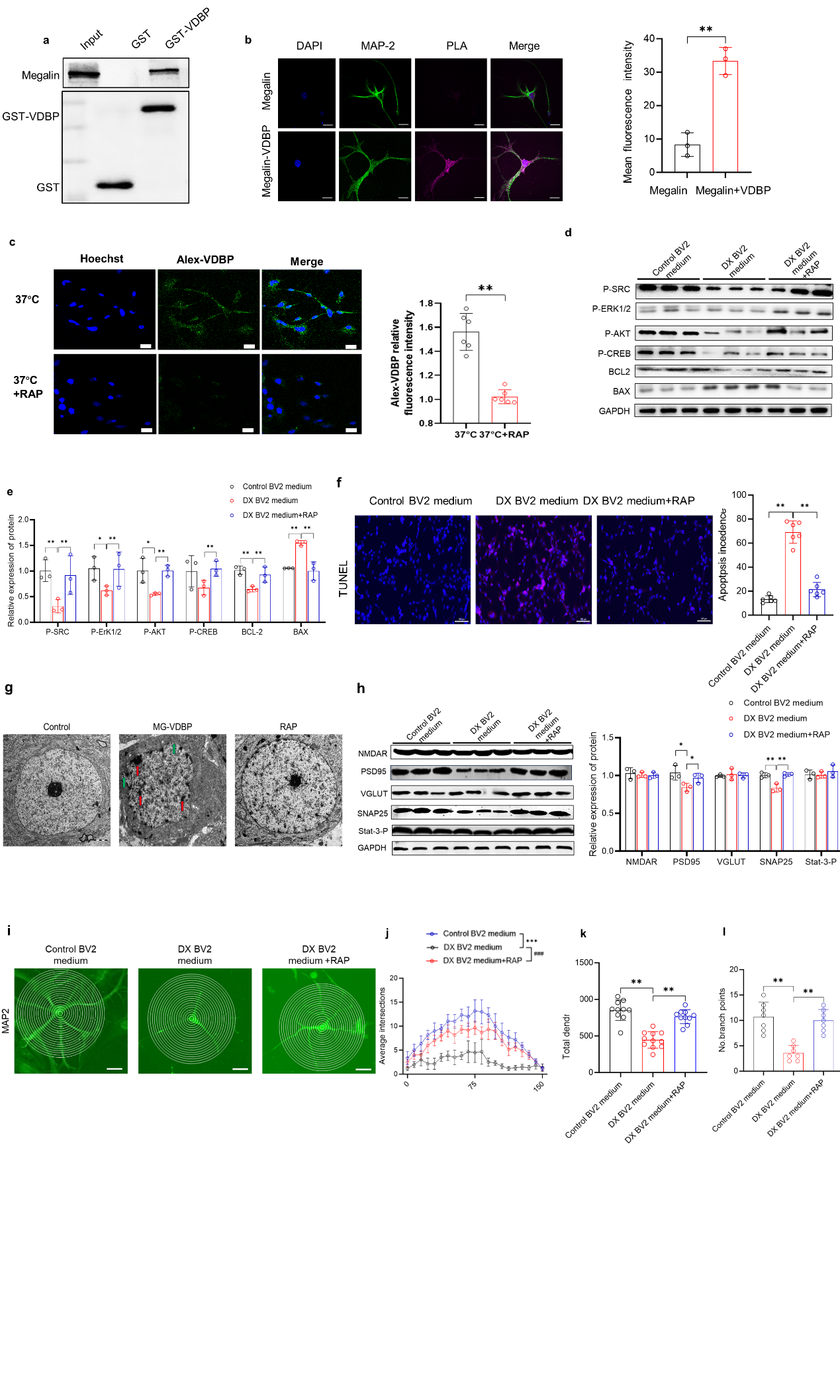
**Supplementary figure S8. MG-derived VDBP binds to megalin and regulates SRC downstream pathways, inducing neuron and synaptic impairment.**

**a** Pull-down of neuron lysates by GST-labeled VDBP and Western blot of GST and megalin. **b** PLA results showed endogenous direct interaction of VDBP and megalin in primary cultured mouse neurons. Green: MAP2 as neuron marker. Red: positive PLA signals. n = 3 biological replicates. Scale bar, 30 μm. **c** Fluorescent-labeled VDBP uptake by cultured primary mouse neurons was blocked by the megalin inhibitor RAP. Quantification of VDBP uptake by primary mouse neurons. n = 6 biological replicates. Scale bar, 20 μm. Western blotting **(d)** and quantification **(e)** of megalin downstream signaling molecules in neurons treated with DX stressed BV2 conditioned medium with or without RAP. n = 3 biological replicates. **f** TUNEL assay with quantification showed that RAP abolished the proapoptotic effects of conditioned medium from DX-treated BV2 cells on primary mouse neurons. n = 6 biological replicates. **g** Transmission electron microscope assay results indicate that conditioned medium from VDBP overexpression BV2 cells induced apoptosis of primary mouse neurons. Chromatin was compacted/fragmented in apoptotic nuclei. RAP ameliorated the proapoptotic effects of MG-derived VDBP on primary mouse neurons. **h** Western blotting and quantification of synaptic proteins in neurons treated with DX stressed BV2 conditioned medium with or without RAP. n = 3 biological replicates. **i-l** Primary mouse cortical neurons were treated with DX-stressed BV2 conditioned medium, either with or without RAP, and then immunostained with an anti-MAP2 antibody. Sholl analysis **(j)** and quantification of average total dendritic length **(k)** and branch number **(l)** of primary cultured cortical neurons (n = 6–10 cells per group). Student’s t-test was used for statistical comparisons between the two groups. For comparisons amongst groups, one-way analysis of variance (ANOVA) followed by Bonferroni post hoc tests was used.. **p* < 0.05, ***p* < 0.01, ****p* < 0.001.

**Supplementary figure S9**


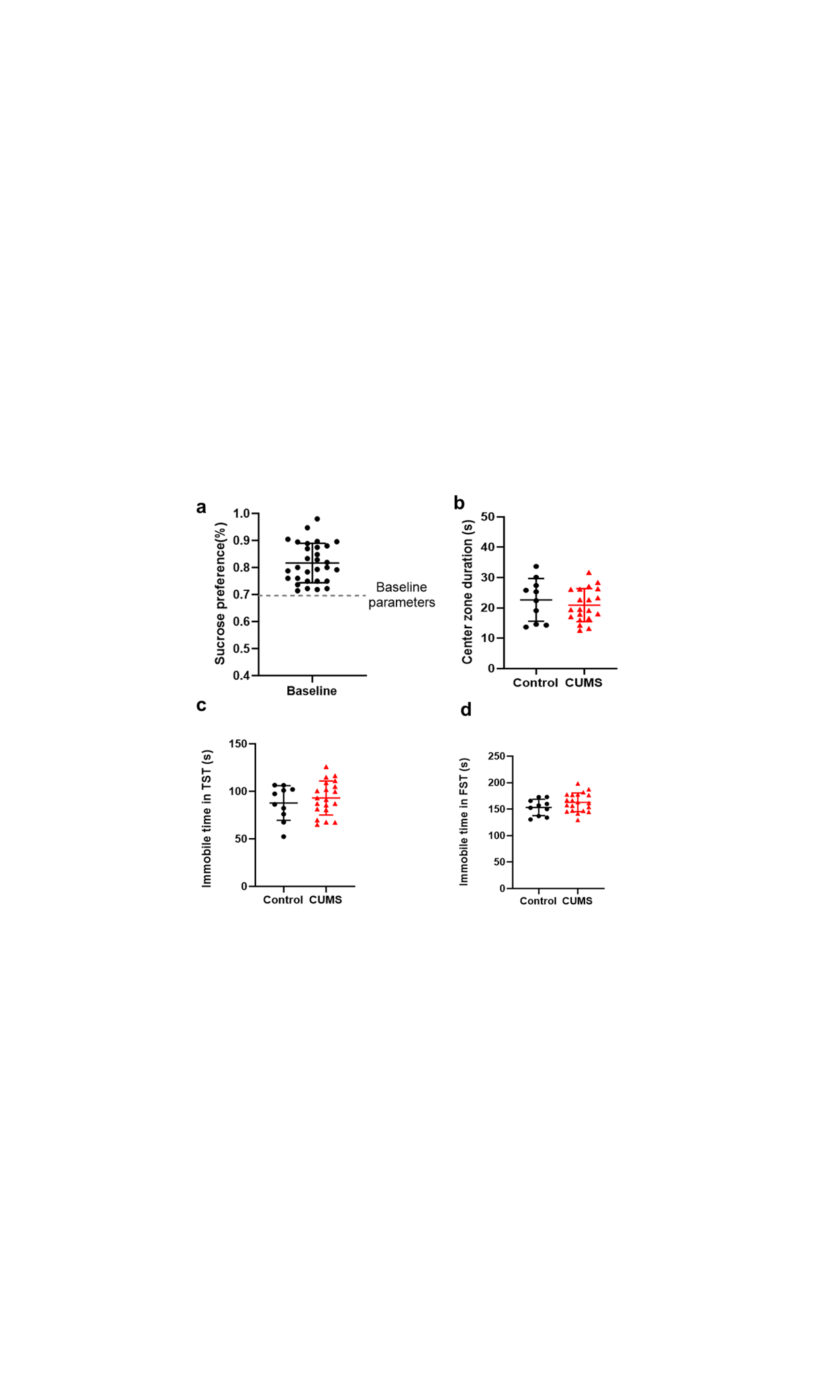
**Supplementary figure S9. Baseline behavioral tests data before CUMS in Figure 1.**

**a** Sucrose preference tests and excluded mice with a baseline sucrose preference below 70% from our study. **b-d** The mice were randomly divided into control and CUMS groups. Additional behavioral tests including open field **(b)**, tail suspension **(c)**, and forced swim tests **(d)** revealed no significant differences between the control and CUMS groups before stress (n = 10 mice in control, n=20 mice in CUMS). Student’s t-test was used for statistical comparisons between the two groups.

**Supplementary Table S1. Primer information**

| Gene | Sense primer | Antisense primer |
| --- | --- | --- |
| mVDBP | CTGACCCCACCTGCTACGA | GTGAACCGGAAAGGGAGCAT |
| mMegalin | CCCTTGCAAGCAGGTCTGTA | GCATGGTGATGGGGAGTTCA |
| Actin | ACTGTCGAGTCGCGTCCA | TCATCCATGGCGAACTGGTG |
| 5’loxP | CAGCAGAGCCTGGAGCAGATTACAG | CAACATGACCTGTGGTAAAGGCACT |
| 3’loxP | TGGCATCATGGAGATGGTGGAAACA | CTGAACTTGCAGCAATCCCCTGC |

**Supplementary Table S2. siRNA oligos**

| siRNA | Sense primer | Antisense primer |
| --- | --- | --- |
| Negative control | UUC UCC GAA CGU GUC ACG UTT | ACGUGACACGUUCGGAGAATT |
| VDBP siRNA | GGCUGAGCCAUCUCAUAAATT | UUUAUGAGAUGGCUCAGCCTT |

**Supplementary Table S3. Key Resources Table**

| REAGENT OR RESOURCE | SOURCE | IDENTIFIER |
| --- | --- | --- |
| Antibodies |  |  |
| Rabbit anti-VDBP | Proteintech | Cat#16922-1-AP |
| Mouse anti-VDBP | Santa Cruz | Cat#sc-365441 |
| Rabbit anti-Iba-1 | Wako | Cat#019-19741 |
| Rabbit anti-NeuN | Proteintech | Cat#26975-1-AP |
| Rabbit anti-GFAP | Proteintech | Cat#16825-1-AP |
| Rabbit anti-PSD95 | abcam | Cat#ab18258 |
| Rabbit anti-Synapsin 1 | abcam | Cat#ab64581 |
| Rabbit anti-Snap25 | abcam | Cat#ab109105 |
| Rabbit anti-Vglut1 | abcam | Cat#ab227805 |
| Rabbit anti-Vgat | Proteintech | Cat#14471-1-AP |
| Mouse anti-β-Actin | Proteintech | Cat#81115-1-RR |
| Rabbit anti-map2 | abcam | Cat#ab32454 |
| Mouse anti-megalin/LRP2 | Santa Cruz | Cat#sc-515772 |
| Goat anti-rabbit | Proteintech | Cat#SA00001-2 |
| Goat anti-mouse | Proteintech | Cat#SA00001-1 |
| FITC anti-mouse CD11b | Biolegend | Cat#101205 |
| Brilliant Violet 605™ anti-mouse CD31 | Biolegend | Cat#102427 |
| APC anti-mouse NCAM-1/CD56 | RD | Cat#FAB7820A-100UG |
| PE anti-mouse ACSA-2 | Miltenyi | Cat#130-116-244 |
| PerCP-Cy5.5 anti-mouse CD45 | BD Pharmingen | Cat#561869 |
| Goat anti-mouse (Alexa Fluor 488) | Proteintech | Cat#SA00013-1 |
| Goat anti-rabbit (Alexa Fluor 488) | Proteintech | Cat#SA00013-2 |
| Goat anti-mouse (Alexa Fluor 594) | Proteintech | Cat#SA00013-3 |
| Goat anti-rabbit (Alexa Fluor 594) | Proteintech | Cat#SA00013-4 |
| Bacterial and virus strains |  |  |
| rAAV-CMV-DIO-Gc-2A-mCherry-WPRE-hGH polyA | BrainVTA | N/A |
| rAAV-CMV-DIO-mCherry-WPRE-hGH polyA | BrainVTA | PT-1203 |
| rAAV-hsyn-EGFP-5'miR-30a-shRNA(Lrp2)-3'-miR30a-WPRE-hGH polyA | BrainVTA | N/A |
| rAAV-hsyn-EGFP-5'miR-30a-shRNA(scramble)-3'-miR30a-WPRE-hGH polyA | BrainVTA | PT-0933 |
| rAAV-Ef1a-DIO-NES-GCaMp6f-WPRE-hGH pA; | BrainVTA | PT-0106 |
| rAAV-VGAT1-CRE-WPRE-hGH pA | BrainVTA | PT-0346 |
| Chemicals, peptides, and recombinant proteins | | |
| Dexamethasone | MedChemExpress | Cat# HY-14686 |
| LPS | Sigma | Cat#L4516 |
| Tamoxifen | Sigma | Cat#T5648 |
| Recombinant Mouse Vitamin D-binding protein | Cusabio | Cat#CSB-YP009306MO |
| ELISA Kit for Vitamin D Binding Protein (DBP) | Cloud-Clone Corp. | Cat#SEB810Mu |
| Cell Counting Kit-8 | Dojindo | Cat#CK04 |
| Golgi-CoxOptimStain Kit | Hitobiotec | Cat#HTKNS1125-2 |
| Click-it plus Tunel 647 | Invitrogen | Cat#C10619 |
| RNAscope Probe - Mm-Gc-C1 | ACD | Cat#1168521-C1 |
| RNAscope Multiplex Fluorescent Reagent Kit v2 | ACD | Cat#323100 |
| Opal 570 Reagent | ACD | Cat#ASOP570 |
| RNAscope H_2_O_2_ and Protease Reagents | ACD | Cat#322381 |
| Experimental models: organisms/strains | | |
| mouse:C57BL/6 | GemPharmatech, Nanjing | N/A |
| mouse:VDBP^loxp/loxp^ | Biocytogen, Beijing | N/A |
| mouse:Cx3cr1-cre^ERT2^ | Jackson laboratory | Stock No: 021160 |
| Software and algorithms |  |  |
| GraphPad Prism 8 | GraphPad software | https://www.graphpad-prism.cn/ |
| MATLAB_R2020b | MathWorks | https://ww2.mathworks.cn/products/matlab.html |
| Fiji | Fiji contributors | https://imagej.net/ |
| ANY-maze | Synaptosoft | https://www.any-maze.com |
| Rstudio | Rstudio | https://rstudio.com/products/rstudio |

**Supplementary Table S4. DEGs in excitatory neurons and inhibitory neurons**

| gene | p_val | avg_log2FC | p_val_adj | Neuon type |
| --- | --- | --- | --- | --- |
| Mid1 | 3.58E-83 | -0.953922796645785 | 8.6E-79 | In |
| Kcnc2 | 4.3E-70 | -0.923365581376667 | 1.03E-65 | In |
| Kcnmb2 | 3.65E-70 | -0.797054295944274 | 8.75E-66 | In |
| Etl4 | 2.2E-55 | -0.724974027995529 | 5.27E-51 | In |
| Elavl2 | 3.09E-63 | -0.715711818308359 | 7.42E-59 | In |
| Grik1 | 4.28E-57 | -0.699819027226992 | 1.03E-52 | In |
| Npas3 | 1.01E-44 | -0.686961254471361 | 2.43E-40 | In |
| Cntnap5c | 1.12E-29 | -0.675121829054678 | 2.68E-25 | In |
| Zfp536 | 3.36E-47 | -0.666499396525199 | 8.07E-43 | In |
| Btbd11 | 6.67E-49 | -0.658197079607949 | 1.6E-44 | In |
| Dner | 2.2E-49 | -0.631751610992162 | 5.27E-45 | In |
| Maf | 7.28E-55 | -0.629185137802786 | 1.75E-50 | In |
| Sox6 | 1.2E-36 | -0.621996658693977 | 2.89E-32 | In |
| Oxr1 | 1.06E-47 | -0.617623221982334 | 2.54E-43 | In |
| Serpini1 | 1.85E-32 | -0.615933033296267 | 4.43E-28 | In |
| Nxph1 | 1.2E-44 | -0.613067899937028 | 2.88E-40 | In |
| Zfp804b | 7.68E-27 | -0.605335936058357 | 1.84E-22 | In |
| Col19a1 | 3.26E-34 | -0.59310151398531 | 7.83E-30 | In |
| Cntnap4 | 8.6E-31 | -0.592171457594935 | 2.07E-26 | In |
| Ptprm | 8.69E-39 | -0.587399098330466 | 2.09E-34 | In |
| Mef2c | 1.61E-39 | -0.575758615762072 | 3.87E-35 | In |
| Snhg11 | 6.91E-79 | -0.572802181199529 | 1.66E-74 | In |
| Galntl6 | 4.14E-43 | -0.571021575904546 | 9.93E-39 | In |
| Erbb4 | 2.14E-28 | -0.566329282191032 | 5.14E-24 | In |
| Kcnc1 | 4.57E-35 | -0.558970518437864 | 1.1E-30 | In |
| Vwc2 | 4.83E-34 | -0.558734990847711 | 1.16E-29 | In |
| Rnf144b | 3.26E-30 | -0.55731562883703 | 7.83E-26 | In |
| Cux2 | 1.8E-41 | -0.546766378654539 | 4.33E-37 | In |
| Dlgap1 | 1.33E-56 | -0.534826865170564 | 3.18E-52 | In |
| 6330411D24Rik | 2.08E-15 | -0.527718447932959 | 5E-11 | In |
| Grin3a | 2.46E-11 | -0.525522182808086 | 5.9E-07 | In |
| Grip1 | 6.58E-53 | -0.525498495629651 | 1.58E-48 | In |
| Kcnip1 | 1.36E-37 | -0.517661707831626 | 3.26E-33 | In |
| 5330434G04Rik | 1.05E-47 | -0.517379560428473 | 2.52E-43 | In |
| Fth1 | 7.97E-29 | -0.516068099821574 | 1.91E-24 | In |
| Dlc1 | 9.18E-43 | -0.508768163315847 | 2.2E-38 | In |
| Cemip | 5.28E-25 | -0.508127412255592 | 1.27E-20 | In |
| Mkx | 2.37E-34 | -0.505002439187543 | 5.69E-30 | In |
| Gabbr2 | 1.46E-40 | -0.502993766749362 | 3.51E-36 | In |
| Gpr176 | 9.77E-18 | -0.499625597234678 | 2.35E-13 | In |
| Trhde | 7.56E-17 | -0.494768200720802 | 1.81E-12 | In |
| Tcf4 | 3.75E-40 | -0.489962852944532 | 9E-36 | In |
| Adcy8 | 1.82E-33 | -0.489012446903498 | 4.36E-29 | In |
| Chrm3 | 5.9E-24 | -0.48899810110039 | 1.42E-19 | In |
| Igf1 | 5.33E-18 | -0.486995715911626 | 1.28E-13 | In |
| Hcn1 | 2.53E-38 | -0.484249073151255 | 6.06E-34 | In |
| Rab3b | 5.08E-22 | -0.483407347954568 | 1.22E-17 | In |
| Ncald | 1.85E-37 | -0.481981988527281 | 4.45E-33 | In |
| Adra1a | 2.64E-25 | -0.481917714103684 | 6.34E-21 | In |
| Mical2 | 2.31E-32 | -0.480487715099897 | 5.55E-28 | In |
| Astn2 | 1.12E-31 | -0.476670689286636 | 2.68E-27 | In |
| Tmem132c | 4.33E-23 | -0.475325477324765 | 1.04E-18 | In |
| A830082K12Rik | 4.51E-35 | -0.471917455400644 | 1.08E-30 | In |
| Slit2 | 5.35E-12 | -0.470545390674075 | 1.28E-07 | In |
| 9630014M24Rik | 1.69E-35 | -0.470298840233166 | 4.05E-31 | In |
| Prickle1 | 4.95E-25 | -0.465902991264898 | 1.19E-20 | In |
| Zmat4 | 5.98E-19 | -0.465533454851604 | 1.44E-14 | In |
| Frmd5 | 9.2E-41 | -0.465408754755084 | 2.21E-36 | In |
| Zbtb16 | 8.14E-36 | -0.465211729005678 | 1.95E-31 | In |
| Chl1 | 3.25E-21 | -0.462495323417054 | 7.81E-17 | In |
| Synpr | 2.17E-08 | -0.461490349235735 | 0.000522 | In |
| Tmem178 | 3.8E-32 | -0.459910206827609 | 9.11E-28 | In |
| Kcnj3 | 1.53E-29 | -0.45929558070779 | 3.68E-25 | In |
| Xkr4 | 5.69E-46 | -0.456339964238546 | 1.37E-41 | In |
| Sh3rf3 | 1.75E-27 | -0.453787162822865 | 4.19E-23 | In |
| Meg3 | 4.76E-65 | -0.452522743678199 | 1.14E-60 | In |
| Apoe | 3.04E-33 | -0.452253842958507 | 7.29E-29 | In |
| Ptchd4 | 5.44E-31 | -0.450115385102932 | 1.31E-26 | In |
| Edil3 | 6.91E-27 | -0.449289334877892 | 1.66E-22 | In |
| Alk | 6.18E-31 | -0.448592113047057 | 1.48E-26 | In |
| Nrg3os | 2.14E-32 | -0.44414521283871 | 5.15E-28 | In |
| Sst | 1.31E-09 | -0.443532917922823 | 3.14E-05 | In |
| Tmem44 | 3.99E-22 | -0.442596706753165 | 9.57E-18 | In |
| Ndst3 | 8.82E-14 | -0.441085689418973 | 2.12E-09 | In |
| Gm20642 | 1.67E-29 | -0.439716964054346 | 4.01E-25 | In |
| Kit | 6.71E-10 | -0.438495666058954 | 1.61E-05 | In |
| Cntnap5a | 2.38E-19 | -0.437556914923948 | 5.72E-15 | In |
| Rasgrf2 | 1E-29 | -0.436768953932531 | 2.41E-25 | In |
| Gab2 | 4.48E-33 | -0.434340082529427 | 1.08E-28 | In |
| 4930517O19Rik | 1.99E-19 | -0.4342232677895 | 4.78E-15 | In |
| Man1c1 | 2.27E-22 | -0.427245359386514 | 5.45E-18 | In |
| Apba1 | 3.38E-28 | -0.426305100337347 | 8.12E-24 | In |
| Nek7 | 4.43E-20 | -0.42618164482092 | 1.06E-15 | In |
| Satb1 | 8.67E-25 | -0.424870886442031 | 2.08E-20 | In |
| Slc24a3 | 7.24E-25 | -0.421413790996996 | 1.74E-20 | In |
| Limch1 | 3.75E-30 | -0.421335715917281 | 9E-26 | In |
| Ankrd24 | 6.18E-28 | -0.420277811078888 | 1.48E-23 | In |
| Gm1992 | 1.98E-21 | -0.4178967595141 | 4.74E-17 | In |
| Sobp | 5.7E-23 | -0.417570050943443 | 1.37E-18 | In |
| mt-Co1 | 1.81E-44 | -0.415524464211362 | 4.35E-40 | In |
| Srrm4 | 9.38E-22 | -0.412148613628337 | 2.25E-17 | In |
| Shank1 | 1.88E-31 | -0.409812223331096 | 4.52E-27 | In |
| Adamts17 | 9.23E-19 | -0.408311659600543 | 2.22E-14 | In |
| Stxbp6 | 9.34E-14 | -0.407673961165956 | 2.24E-09 | In |
| Stambpl1 | 3.77E-19 | -0.40530805709096 | 9.05E-15 | In |
| Vsnl1 | 7.8E-29 | -0.403189163141273 | 1.87E-24 | In |
| A830018L16Rik | 2.47E-12 | -0.403187860200285 | 5.92E-08 | In |
| Gm35188 | 8.1E-19 | -0.401843787239064 | 1.94E-14 | In |
| Nap1l5 | 6.45E-23 | -0.400372678626288 | 1.55E-18 | In |
| Sdk1 | 6.05E-13 | -0.398256888180081 | 1.45E-08 | In |
| Sntg1 | 1.98E-30 | -0.398178580845361 | 4.75E-26 | In |
| Tmeff2 | 8.04E-10 | -0.394954729692826 | 1.93E-05 | In |
| Garnl3 | 5.73E-21 | -0.394368430823157 | 1.37E-16 | In |
| Arhgap5 | 1.91E-22 | -0.393464165137875 | 4.57E-18 | In |
| Sox5 | 7.58E-20 | -0.392850941658193 | 1.82E-15 | In |
| Snhg4 | 7.65E-21 | -0.392230022386694 | 1.84E-16 | In |
| Hunk | 1.23E-20 | -0.391617024206612 | 2.95E-16 | In |
| Tmem132d | 9.03E-15 | -0.389217196752591 | 2.17E-10 | In |
| Tshz3 | 3.93E-23 | -0.387695162400437 | 9.44E-19 | In |
| Sox5os4 | 6.13E-18 | -0.387440586793129 | 1.47E-13 | In |
| mt-Nd2 | 1.53E-24 | -0.387097211437394 | 3.68E-20 | In |
| Cntnap2 | 2.14E-23 | -0.381923582942098 | 5.13E-19 | In |
| Afap1 | 3.03E-24 | -0.381665064897151 | 7.27E-20 | In |
| Ube3a | 5.54E-34 | -0.381396739039197 | 1.33E-29 | In |
| Ankrd55 | 7.51E-26 | -0.381049000040587 | 1.8E-21 | In |
| Thrb | 3.44E-26 | -0.379846720159724 | 8.26E-22 | In |
| Tafa2 | 7.19E-13 | -0.379229108613194 | 1.73E-08 | In |
| Mpped1 | 1.23E-19 | -0.379219458711453 | 2.95E-15 | In |
| Sparcl1 | 1.35E-21 | -0.379046679074051 | 3.25E-17 | In |
| Luzp2 | 1.12E-15 | -0.372700210669531 | 2.68E-11 | In |
| Cpeb3 | 3.04E-21 | -0.372458220539791 | 7.29E-17 | In |
| Klhl32 | 1.53E-27 | -0.371452326934127 | 3.67E-23 | In |
| Cox8a | 2.27E-22 | -0.370791272727404 | 5.44E-18 | In |
| Nrg3 | 4.75E-34 | -0.370760437451782 | 1.14E-29 | In |
| Hmgn3 | 2.29E-16 | -0.370479193888693 | 5.51E-12 | In |
| mt-Atp6 | 7.72E-54 | -0.370421218273475 | 1.85E-49 | In |
| Ccdc136 | 2.14E-25 | -0.369885305822729 | 5.14E-21 | In |
| Fgf1 | 2.2E-19 | -0.367944599807669 | 5.28E-15 | In |
| Grk3 | 6.71E-24 | -0.365865332918349 | 1.61E-19 | In |
| Pdzrn3 | 3.21E-14 | -0.36586058244011 | 7.7E-10 | In |
| Gm28175 | 1.82E-09 | -0.365327243105031 | 4.36E-05 | In |
| Fmn1 | 2.67E-18 | -0.364086770575907 | 6.4E-14 | In |
| Sulf2 | 7.88E-21 | -0.363120573522621 | 1.89E-16 | In |
| Syn2 | 3.65E-20 | -0.36283167608751 | 8.77E-16 | In |
| Clstn2 | 1.64E-13 | -0.36251124219898 | 3.93E-09 | In |
| Rock2 | 1.79E-18 | -0.360616560261334 | 4.31E-14 | In |
| Magi1 | 5.5E-25 | -0.359483046547872 | 1.32E-20 | In |
| Gm13629 | 5.89E-14 | -0.355941538106842 | 1.41E-09 | In |
| Runx2 | 2.16E-14 | -0.353366862252144 | 5.18E-10 | In |
| Adgra1 | 4.35E-21 | -0.353028546120523 | 1.04E-16 | In |
| Mcc | 1.53E-19 | -0.352279158333053 | 3.68E-15 | In |
| Aff2 | 5.67E-21 | -0.351582364181692 | 1.36E-16 | In |
| Pvt1 | 1.62E-17 | -0.350795043138991 | 3.88E-13 | In |
| Lsamp | 1.89E-26 | -0.350317007765808 | 4.54E-22 | In |
| Frmd4a | 5.34E-28 | -0.350204774765465 | 1.28E-23 | In |
| Slc44a5 | 1.44E-09 | -0.349748298741524 | 3.45E-05 | In |
| mt-Nd1 | 4.67E-25 | -0.349340095091676 | 1.12E-20 | In |
| Tenm1 | 3.42E-19 | -0.349016113000211 | 8.21E-15 | In |
| Iqgap2 | 5.58E-09 | -0.348799432655433 | 0.000134 | In |
| Clstn3 | 3.71E-20 | -0.348480606882116 | 8.9E-16 | In |
| 4933413L06Rik | 8.72E-15 | -0.347932255462337 | 2.09E-10 | In |
| Ablim1 | 5.67E-25 | -0.347930126520856 | 1.36E-20 | In |
| Eya4 | 3.59E-12 | -0.34736649475171 | 8.62E-08 | In |
| Osbpl3 | 7.22E-21 | -0.34722981790608 | 1.73E-16 | In |
| Gm16083 | 2.43E-15 | -0.346284143422762 | 5.84E-11 | In |
| Ank1 | 6.22E-21 | -0.346142297179791 | 1.49E-16 | In |
| Manba | 2.43E-22 | -0.346097213408758 | 5.83E-18 | In |
| Gm48321 | 2.04E-10 | -0.345348799800234 | 4.89E-06 | In |
| Pam | 2.7E-24 | -0.34444840320749 | 6.48E-20 | In |
| Tm6sf1 | 2.02E-18 | -0.34298587901514 | 4.85E-14 | In |
| Zcchc18 | 9.64E-15 | -0.341353197802778 | 2.31E-10 | In |
| Car10 | 1.1E-13 | -0.340702555857637 | 2.65E-09 | In |
| A530058N18Rik | 1.77E-19 | -0.338457773209998 | 4.24E-15 | In |
| Fgf12 | 1.09E-22 | -0.334837827085341 | 2.62E-18 | In |
| Myo1e | 1.2E-09 | -0.334834496693621 | 2.89E-05 | In |
| Cacna1a | 3.04E-24 | -0.333947821837215 | 7.3E-20 | In |
| Nrip3 | 4.08E-15 | -0.333369954826043 | 9.78E-11 | In |
| Haghl | 3.81E-15 | -0.332439370225098 | 9.15E-11 | In |
| Pde5a | 2.71E-16 | -0.332166561447886 | 6.5E-12 | In |
| Utrn | 1.32E-12 | -0.330878418189025 | 3.17E-08 | In |
| Rgs6 | 1.35E-06 | -0.33002432012209 | 0.032491 | In |
| Rtl4 | 1.13E-07 | -0.32843794181434 | 0.00272 | In |
| Kcns3 | 4.48E-19 | -0.328209723398963 | 1.08E-14 | In |
| Srcin1 | 1.22E-19 | -0.328079042911201 | 2.93E-15 | In |
| Rph3a | 1.88E-11 | -0.327858208248486 | 4.51E-07 | In |
| Frmpd3 | 1.03E-22 | -0.327039277957233 | 2.47E-18 | In |
| Agbl4 | 2.31E-23 | -0.326277318889385 | 5.53E-19 | In |
| Rab27b | 6.54E-16 | -0.325541992626686 | 1.57E-11 | In |
| Oprd1 | 4.3E-14 | -0.325203130532577 | 1.03E-09 | In |
| Fnip2 | 1.18E-15 | -0.320758131052394 | 2.83E-11 | In |
| Trpc6 | 1.71E-06 | -0.320258143320194 | 0.041076 | In |
| Wwox | 5.85E-17 | -0.319800275328217 | 1.4E-12 | In |
| Cntn6 | 8.58E-07 | -0.31664088272415 | 0.020598 | In |
| Dnajc6 | 2.79E-18 | -0.316179111428823 | 6.69E-14 | In |
| Plxdc2 | 3.28E-09 | -0.315891688962185 | 7.87E-05 | In |
| Lypd6b | 1.41E-12 | -0.315185288070525 | 3.38E-08 | In |
| Peg3 | 1.86E-18 | -0.314755356514141 | 4.47E-14 | In |
| Shisa6 | 1.62E-08 | -0.313423541191463 | 0.000389 | In |
| Camk2n1 | 1.8E-17 | -0.311930819195784 | 4.31E-13 | In |
| Scn9a | 9.67E-09 | -0.30828862549638 | 0.000232 | In |
| Ubash3b | 1.18E-20 | -0.308145322063726 | 2.83E-16 | In |
| A330076H08Rik | 4.32E-22 | -0.307848568603923 | 1.04E-17 | In |
| Mast4 | 7.83E-18 | -0.307416196684559 | 1.88E-13 | In |
| St5 | 3.27E-16 | -0.307103696568308 | 7.84E-12 | In |
| Akain1 | 2.36E-18 | -0.305730546551398 | 5.67E-14 | In |
| Kif21a | 5.68E-13 | -0.304793305152076 | 1.36E-08 | In |
| Gm48091 | 4.23E-14 | -0.303965297957102 | 1.02E-09 | In |
| St3gal6 | 2.63E-14 | -0.302621102001585 | 6.32E-10 | In |
| Opcml | 4.7E-09 | -0.301903010214444 | 0.000113 | In |
| Slc25a3 | 7.17E-13 | -0.301811342011229 | 1.72E-08 | In |
| Coro6 | 3.22E-17 | -0.301649005286633 | 7.72E-13 | In |
| Gabra1 | 1.54E-12 | -0.301391089756592 | 3.71E-08 | In |
| Atrnl1 | 6.09E-13 | -0.299386203176515 | 1.46E-08 | In |
| Rab6a | 9.94E-15 | -0.299156181128785 | 2.38E-10 | In |
| Rapgef4 | 1.67E-16 | -0.299120399828269 | 4.02E-12 | In |
| Pitpnm3 | 8.45E-16 | -0.29897355024993 | 2.03E-11 | In |
| Bckdhb | 1.14E-14 | -0.296922042277089 | 2.74E-10 | In |
| mt-Nd3 | 8.16E-13 | -0.296687614666382 | 1.96E-08 | In |
| Cerk | 2.82E-14 | -0.296604012573404 | 6.76E-10 | In |
| Iqsec3 | 2.27E-15 | -0.296384224087943 | 5.44E-11 | In |
| Col26a1 | 1.19E-09 | -0.293609683904 | 2.86E-05 | In |
| Gria4 | 7.95E-19 | -0.293181939239249 | 1.91E-14 | In |
| mt-Nd4 | 5.41E-28 | -0.291899519047314 | 1.3E-23 | In |
| Amph | 1.42E-15 | -0.290519852903062 | 3.41E-11 | In |
| Mir124a-1hg | 8.04E-17 | -0.290059230292002 | 1.93E-12 | In |
| Slc35f4 | 5.99E-16 | -0.289415064664169 | 1.44E-11 | In |
| Rapgef4os1 | 3.09E-10 | -0.289164742448731 | 7.43E-06 | In |
| Ltbp1 | 9.31E-09 | -0.288564156154142 | 0.000224 | In |
| Bend6 | 9.81E-16 | -0.288179740887906 | 2.36E-11 | In |
| Slc7a1 | 2.3E-14 | -0.288020548221285 | 5.53E-10 | In |
| Gnas | 2.61E-20 | -0.287361769295728 | 6.28E-16 | In |
| Rbms3 | 3.49E-15 | -0.287207053885228 | 8.38E-11 | In |
| Ptprg | 2.01E-15 | -0.286628578453348 | 4.82E-11 | In |
| Gm12709 | 1.08E-09 | -0.286466180853696 | 2.59E-05 | In |
| Tmem132cos | 9.37E-15 | -0.286260341966225 | 2.25E-10 | In |
| Ptprr | 1.35E-09 | -0.285677627471745 | 3.25E-05 | In |
| Moxd1 | 3.52E-10 | -0.28471816208796 | 8.46E-06 | In |
| Rab3c | 5.72E-11 | -0.28465569000029 | 1.37E-06 | In |
| Atp6v0b | 3.05E-17 | -0.284441913951763 | 7.31E-13 | In |
| Iqsec1 | 7.2E-13 | -0.283859548679143 | 1.73E-08 | In |
| Grip1os2 | 2.77E-15 | -0.283593826114345 | 6.65E-11 | In |
| Nhs | 8.36E-17 | -0.283338909063903 | 2.01E-12 | In |
| Ankrd44 | 2.07E-11 | -0.282839882786307 | 4.96E-07 | In |
| Lrrc8b | 5.13E-11 | -0.282657297107538 | 1.23E-06 | In |
| Evi5 | 2E-11 | -0.281740712538493 | 4.8E-07 | In |
| Impact | 1E-17 | -0.280901921890999 | 2.4E-13 | In |
| Epha10 | 2.88E-13 | -0.280697887366259 | 6.92E-09 | In |
| Mast2 | 1.08E-19 | -0.279999481732624 | 2.58E-15 | In |
| Stac2 | 2.4E-18 | -0.279412937691105 | 5.77E-14 | In |
| Cdh13 | 1.81E-07 | -0.278796007434621 | 0.004354 | In |
| B230216N24Rik | 4.95E-08 | -0.277762932985105 | 0.001189 | In |
| Myrip | 2.85E-14 | -0.277638213344895 | 6.85E-10 | In |
| Frmpd1 | 1.57E-14 | -0.277573540202238 | 3.78E-10 | In |
| Kif26b | 1.05E-07 | -0.275918513945451 | 0.00253 | In |
| Camk1 | 8.68E-14 | -0.275493600143388 | 2.08E-09 | In |
| Tmem65 | 7.87E-16 | -0.275288031414107 | 1.89E-11 | In |
| Dock9 | 2.99E-14 | -0.274593227285847 | 7.18E-10 | In |
| Tcp11l1 | 2.11E-13 | -0.274124158594391 | 5.07E-09 | In |
| Rims4 | 9.19E-12 | -0.273980151892926 | 2.21E-07 | In |
| 1700025G04Rik | 2.57E-13 | -0.272723481673286 | 6.16E-09 | In |
| Pknox2 | 6.79E-15 | -0.272329695787344 | 1.63E-10 | In |
| Sh3rf1 | 6.94E-13 | -0.271959981485123 | 1.67E-08 | In |
| 2210408F21Rik | 7.32E-15 | -0.271769875991349 | 1.76E-10 | In |
| Luzp1 | 1.34E-09 | -0.271574504892877 | 3.22E-05 | In |
| Usp13 | 4.32E-12 | -0.271261326395122 | 1.04E-07 | In |
| Daam1 | 1.32E-14 | -0.270337511072239 | 3.16E-10 | In |
| Gm21798 | 1.96E-14 | -0.270324356131001 | 4.7E-10 | In |
| Nt5dc3 | 1.25E-12 | -0.26986208984707 | 3E-08 | In |
| Lypd6 | 4.6E-12 | -0.269491800459917 | 1.1E-07 | In |
| Spock1 | 3E-10 | -0.269022356229754 | 7.21E-06 | In |
| Rpl41 | 5.73E-12 | -0.268730289618768 | 1.37E-07 | In |
| Zeb2 | 2.32E-29 | -0.268499132625174 | 5.57E-25 | In |
| 9630002D21Rik | 7.06E-09 | -0.267118027514859 | 0.00017 | In |
| Shisa4 | 5.84E-14 | -0.266826476408043 | 1.4E-09 | In |
| Cdh4 | 3.75E-07 | -0.266568879023658 | 0.008993 | In |
| Cadps | 1.09E-22 | -0.266537729307533 | 2.62E-18 | In |
| Fam13b | 1.9E-12 | -0.265588588597235 | 4.56E-08 | In |
| Kcnt2 | 1.68E-06 | -0.265420077189435 | 0.040284 | In |
| Dlgap2 | 2.6E-15 | -0.265389505005472 | 6.25E-11 | In |
| Cpeb1 | 2.02E-12 | -0.264930422235204 | 4.85E-08 | In |
| Gm1604a | 1.32E-06 | -0.263738477685795 | 0.03178 | In |
| mt-Nd5 | 1.2E-17 | -0.263622271647656 | 2.88E-13 | In |
| Gm15398 | 4.52E-10 | -0.263038729344619 | 1.09E-05 | In |
| Arnt2 | 1.35E-14 | -0.262958138219619 | 3.24E-10 | In |
| Tmem108 | 2.26E-09 | -0.262895027631992 | 5.42E-05 | In |
| Kcnh7 | 3.57E-10 | -0.262531405547712 | 8.56E-06 | In |
| Wasf3 | 3.45E-12 | -0.26224024057463 | 8.29E-08 | In |
| 3110021N24Rik | 4.81E-11 | -0.261909881329893 | 1.15E-06 | In |
| Sars | 6.53E-12 | -0.261593461402655 | 1.57E-07 | In |
| Mtss2 | 7.9E-15 | -0.26102624796795 | 1.9E-10 | In |
| Arhgef11 | 3.22E-18 | -0.26062995260527 | 7.73E-14 | In |
| Cit | 1.88E-09 | -0.260205800117632 | 4.51E-05 | In |
| Ano4 | 1.78E-08 | -0.259117855755543 | 0.000427 | In |
| Asic2 | 1.85E-13 | -0.25796371730732 | 4.45E-09 | In |
| BC005561 | 7.38E-11 | -0.257810783323023 | 1.77E-06 | In |
| Arhgap15 | 1.02E-14 | -0.257426635287567 | 2.45E-10 | In |
| Gm31218 | 1.96E-11 | -0.257330274618483 | 4.69E-07 | In |
| Slc23a2 | 1.77E-11 | -0.256695776654436 | 4.25E-07 | In |
| Grip2 | 2.91E-16 | -0.256379924497489 | 6.98E-12 | In |
| Ggt7 | 1.56E-12 | -0.256300580594337 | 3.74E-08 | In |
| Eif4g3 | 7.39E-19 | -0.25583706545768 | 1.77E-14 | In |
| Cacng7 | 6.84E-11 | -0.255595482462628 | 1.64E-06 | In |
| Il1rap | 2.32E-10 | -0.255586007695465 | 5.56E-06 | In |
| 4930509J09Rik | 2.72E-07 | -0.253927730497533 | 0.00653 | In |
| Rims1 | 1.11E-12 | -0.253709251789612 | 2.66E-08 | In |
| Far2 | 5.25E-09 | -0.253653315696652 | 0.000126 | In |
| Gabrb2 | 9.18E-08 | -0.253298167258982 | 0.002204 | In |
| Lhx6 | 1.25E-16 | -0.253108900787896 | 2.99E-12 | In |
| Rmdn1 | 5.76E-13 | -0.253011736751712 | 1.38E-08 | In |
| Syngr3 | 3.34E-10 | -0.251989379546409 | 8.01E-06 | In |
| Slc38a2 | 2.57E-11 | -0.251447077183974 | 6.16E-07 | In |
| Dip2a | 1.39E-12 | -0.250984611101958 | 3.34E-08 | In |
| Snhg20 | 9.31E-13 | -0.250972833359713 | 2.23E-08 | In |
| Abhd18 | 6.03E-12 | -0.250713448754319 | 1.45E-07 | In |
| Meis2 | 7.6E-110 | 2.795978 | 1.8E-105 | In |
| Pde7b | 8.66E-56 | 2.430569 | 2.08E-51 | In |
| Pbx3 | 1.03E-78 | 2.291113 | 2.48E-74 | In |
| Dgkb | 5.12E-67 | 2.058874 | 1.23E-62 | In |
| Rarb | 6.53E-41 | 2.052341 | 1.57E-36 | In |
| Foxp2 | 5.21E-76 | 1.947211 | 1.25E-71 | In |
| Ryr3 | 6.1E-61 | 1.927407 | 1.46E-56 | In |
| Phactr1 | 8.53E-49 | 1.922308 | 2.05E-44 | In |
| Gm10754 | 2.66E-62 | 1.779639 | 6.38E-58 | In |
| Foxp1 | 2.85E-44 | 1.6089 | 6.85E-40 | In |
| Rgs9 | 1.3E-51 | 1.494147 | 3.12E-47 | In |
| Dach1 | 6.21E-59 | 1.404842 | 1.49E-54 | In |
| Zbtb20 | 1.49E-51 | 1.388881 | 3.57E-47 | In |
| Cacnb2 | 8.21E-37 | 1.35306 | 1.97E-32 | In |
| Camk4 | 7.65E-39 | 1.352719 | 1.84E-34 | In |
| 2610307P16Rik | 3.13E-16 | 1.326348 | 7.52E-12 | In |
| Celf2 | 6.61E-73 | 1.31842 | 1.59E-68 | In |
| Pde4b | 1.07E-35 | 1.309619 | 2.57E-31 | In |
| Nrg1 | 4.98E-25 | 1.30516 | 1.19E-20 | In |
| Arpp21 | 2.3E-53 | 1.199809 | 5.52E-49 | In |
| Dcc | 3.42E-42 | 1.13489 | 8.21E-38 | In |
| Gm28905 | 8.93E-43 | 1.118278 | 2.14E-38 | In |
| Slit3 | 2.88E-23 | 1.116774 | 6.92E-19 | In |
| Pdzd2 | 3.86E-30 | 1.096477 | 9.27E-26 | In |
| Galnt13 | 1.04E-45 | 1.093839 | 2.51E-41 | In |
| 6530403H02Rik | 3.47E-50 | 1.075903 | 8.33E-46 | In |
| Rgs20 | 2.43E-39 | 1.070119 | 5.83E-35 | In |
| Tiam2 | 3.67E-31 | 1.064377 | 8.81E-27 | In |
| Zfhx3 | 7.56E-43 | 1.03693 | 1.81E-38 | In |
| Adcy5 | 5.38E-40 | 1.007641 | 1.29E-35 | In |
| Mpped2 | 1.67E-40 | 1.007285 | 4.02E-36 | In |
| Baiap2 | 1.38E-54 | 1.003038 | 3.31E-50 | In |
| Slc4a4 | 1.15E-32 | 1.002739 | 2.77E-28 | In |
| Nnat | 4.73E-22 | 0.99824 | 1.13E-17 | In |
| Caln1 | 7.64E-30 | 0.981099 | 1.83E-25 | In |
| Pde1c | 1.33E-32 | 0.97835 | 3.19E-28 | In |
| Rasgef1b | 5.42E-45 | 0.965549 | 1.3E-40 | In |
| Zswim6 | 5.42E-31 | 0.943081 | 1.3E-26 | In |
| Arhgap10 | 5.42E-41 | 0.939094 | 1.3E-36 | In |
| Pbx1 | 4.42E-23 | 0.930426 | 1.06E-18 | In |
| Elmo1 | 2.01E-51 | 0.928251 | 4.83E-47 | In |
| Cacna1e | 5.44E-42 | 0.92522 | 1.31E-37 | In |
| Ephb1 | 8E-50 | 0.922575 | 1.92E-45 | In |
| Sgcz | 8.49E-18 | 0.877537 | 2.04E-13 | In |
| Strn | 8.3E-26 | 0.863058 | 1.99E-21 | In |
| Atp2b1 | 3.46E-34 | 0.860843 | 8.3E-30 | In |
| Npas2 | 1.49E-32 | 0.858948 | 3.57E-28 | In |
| Gm49906 | 1.02E-49 | 0.856924 | 2.45E-45 | In |
| Pcsk2 | 3.03E-29 | 0.847128 | 7.27E-25 | In |
| Cdh8 | 1.51E-24 | 0.836856 | 3.61E-20 | In |
| Maml3 | 1.62E-20 | 0.831267 | 3.89E-16 | In |
| 2010300C02Rik | 4.62E-48 | 0.814065 | 1.11E-43 | In |
| Zeb1 | 1.86E-38 | 0.795997 | 4.47E-34 | In |
| Ppp1r9a | 1.7E-35 | 0.795252 | 4.08E-31 | In |
| Ppp3ca | 2.26E-38 | 0.794205 | 5.43E-34 | In |
| Cacna2d3 | 1.73E-18 | 0.793292 | 4.15E-14 | In |
| Lrrc7 | 1.12E-59 | 0.78761 | 2.7E-55 | In |
| Srgap1 | 1.11E-26 | 0.786585 | 2.67E-22 | In |
| Gnal | 1.15E-17 | 0.774224 | 2.76E-13 | In |
| Ano3 | 3.68E-26 | 0.772884 | 8.84E-22 | In |
| Pard3 | 1.33E-27 | 0.770527 | 3.2E-23 | In |
| Adamts3 | 1.68E-39 | 0.765949 | 4.03E-35 | In |
| Gprin3 | 2.59E-32 | 0.750723 | 6.23E-28 | In |
| Gng7 | 5.37E-23 | 0.74982 | 1.29E-18 | In |
| Gpm6b | 9.8E-30 | 0.741942 | 2.35E-25 | In |
| Hs6st2 | 4.22E-33 | 0.741555 | 1.01E-28 | In |
| Trpm3 | 7.74E-11 | 0.73988 | 1.86E-06 | In |
| Zdhhc14 | 2.03E-20 | 0.738886 | 4.87E-16 | In |
| Gpr158 | 4.48E-32 | 0.737394 | 1.07E-27 | In |
| Sh3rf2 | 7.9E-49 | 0.736246 | 1.9E-44 | In |
| Drd2 | 1.33E-29 | 0.73424 | 3.19E-25 | In |
| Rnf150 | 2.58E-30 | 0.731488 | 6.2E-26 | In |
| Kcnd2 | 9.08E-18 | 0.727664 | 2.18E-13 | In |
| Lmo7 | 2.33E-35 | 0.72483 | 5.58E-31 | In |
| Unc13c | 2.66E-27 | 0.72237 | 6.38E-23 | In |
| Kcnn3 | 4.27E-48 | 0.70864 | 1.03E-43 | In |
| Penk | 5.26E-28 | 0.70682 | 1.26E-23 | In |
| Syt6 | 1.93E-40 | 0.695538 | 4.62E-36 | In |
| Fras1 | 2.36E-32 | 0.69514 | 5.67E-28 | In |
| Gm44593 | 1.43E-38 | 0.691287 | 3.42E-34 | In |
| Epha4 | 4.51E-40 | 0.683359 | 1.08E-35 | In |
| Sdk2 | 3.3E-10 | 0.6811 | 7.91E-06 | In |
| Garem1 | 4.43E-24 | 0.678155 | 1.06E-19 | In |
| Stox2 | 4.03E-25 | 0.676896 | 9.67E-21 | In |
| Igfbpl1 | 1.21E-22 | 0.674417 | 2.9E-18 | In |
| Mapk4 | 4.49E-26 | 0.668767 | 1.08E-21 | In |
| St6galnac3 | 3.97E-18 | 0.665161 | 9.52E-14 | In |
| Foxo1 | 1.17E-43 | 0.662543 | 2.8E-39 | In |
| Gm2164 | 9.95E-46 | 0.661022 | 2.39E-41 | In |
| Grm3 | 2.5E-14 | 0.660869 | 6E-10 | In |
| Ntng1 | 3.66E-20 | 0.654086 | 8.78E-16 | In |
| Kcnq5 | 4.13E-17 | 0.648662 | 9.91E-13 | In |
| Serpine2 | 1.26E-26 | 0.648372 | 3.02E-22 | In |
| Dlg1 | 2.65E-24 | 0.647566 | 6.35E-20 | In |
| Osbpl8 | 4.14E-24 | 0.645468 | 9.94E-20 | In |
| Nlgn1 | 5.18E-29 | 0.64064 | 1.24E-24 | In |
| Hs6st3 | 2.35E-17 | 0.632726 | 5.64E-13 | In |
| Vcan | 8.56E-23 | 0.632229 | 2.05E-18 | In |
| Hs3st4 | 1.65E-25 | 0.629261 | 3.95E-21 | In |
| Lhfpl3 | 1.73E-25 | 0.628976 | 4.16E-21 | In |
| Cobl | 1.47E-26 | 0.623735 | 3.52E-22 | In |
| 9530059O14Rik | 7.34E-20 | 0.61907 | 1.76E-15 | In |
| Lzts1 | 7.81E-39 | 0.617675 | 1.87E-34 | In |
| Rps6ka5 | 7.98E-26 | 0.614803 | 1.92E-21 | In |
| Pde1b | 9.56E-38 | 0.613822 | 2.29E-33 | In |
| C79798 | 5.73E-33 | 0.605239 | 1.38E-28 | In |
| Lrrk2 | 3.2E-20 | 0.604945 | 7.67E-16 | In |
| Mrtfb | 4.2E-30 | 0.604402 | 1.01E-25 | In |
| Ptk2b | 5.46E-41 | 0.603278 | 1.31E-36 | In |
| Itpr1 | 5.26E-15 | 0.602863 | 1.26E-10 | In |
| Cnksr2 | 5.57E-25 | 0.598048 | 1.34E-20 | In |
| Cdk17 | 6.64E-26 | 0.596353 | 1.59E-21 | In |
| Slc35f3 | 3.91E-15 | 0.596131 | 9.39E-11 | In |
| Kctd1 | 3.9E-22 | 0.595584 | 9.35E-18 | In |
| Gm26871 | 1.61E-22 | 0.595563 | 3.86E-18 | In |
| Gabra2 | 2.84E-29 | 0.59403 | 6.82E-25 | In |
| Auts2 | 1.82E-38 | 0.593237 | 4.38E-34 | In |
| Nedd4l | 2.48E-26 | 0.58697 | 5.94E-22 | In |
| Dgki | 5.7E-20 | 0.579418 | 1.37E-15 | In |
| Sox4 | 3.54E-07 | 0.578388 | 0.008494 | In |
| Brinp1 | 7.35E-22 | 0.57647 | 1.76E-17 | In |
| Bcl11b | 4.08E-25 | 0.573874 | 9.8E-21 | In |
| Hs3st5 | 1.07E-17 | 0.567091 | 2.58E-13 | In |
| Ngef | 5.26E-36 | 0.565019 | 1.26E-31 | In |
| Grm7 | 4.54E-13 | 0.562075 | 1.09E-08 | In |
| Prkcb | 6.53E-17 | 0.556357 | 1.57E-12 | In |
| Kcnab1 | 1.12E-12 | 0.556256 | 2.69E-08 | In |
| Diaph2 | 1.45E-20 | 0.554937 | 3.49E-16 | In |
| Pde10a | 6.96E-08 | 0.553361 | 0.00167 | In |
| Peli2 | 4.39E-29 | 0.552508 | 1.05E-24 | In |
| Ebf1 | 8.54E-23 | 0.550695 | 2.05E-18 | In |
| Meis1 | 3.1E-20 | 0.547493 | 7.44E-16 | In |
| Actn2 | 6.01E-27 | 0.545678 | 1.44E-22 | In |
| Vat1l | 1.66E-17 | 0.536934 | 3.98E-13 | In |
| Slc24a4 | 9.42E-43 | 0.536062 | 2.26E-38 | In |
| Chd7 | 3.18E-12 | 0.531917 | 7.64E-08 | In |
| Tenm4 | 5.77E-25 | 0.531238 | 1.39E-20 | In |
| Sez6 | 3.79E-27 | 0.523598 | 9.1E-23 | In |
| Gm10848 | 1.14E-35 | 0.522653 | 2.73E-31 | In |
| Lmo3 | 2.95E-23 | 0.522044 | 7.08E-19 | In |
| Tbc1d8 | 5.75E-26 | 0.518035 | 1.38E-21 | In |
| Prkch | 1.57E-40 | 0.515624 | 3.77E-36 | In |
| Tead1 | 1.9E-11 | 0.513896 | 4.56E-07 | In |
| 5730522E02Rik | 9.38E-22 | 0.513707 | 2.25E-17 | In |
| Inf2 | 7.36E-34 | 0.512119 | 1.77E-29 | In |
| Pcp4 | 9.65E-35 | 0.508002 | 2.32E-30 | In |
| Sv2c | 1.7E-30 | 0.505925 | 4.09E-26 | In |
| Fry | 3.55E-18 | 0.503216 | 8.52E-14 | In |
| Cpne5 | 2.91E-22 | 0.500765 | 6.98E-18 | In |
| Ccdc88c | 1.35E-34 | 0.500612 | 3.25E-30 | In |
| Smpd3 | 5.56E-18 | 0.50056 | 1.33E-13 | In |
| Klhl2 | 1.48E-13 | 0.496192 | 3.56E-09 | In |
| Mhrt | 1.26E-40 | 0.495882 | 3.03E-36 | In |
| Tshz1 | 1.52E-10 | 0.494787 | 3.66E-06 | In |
| Gm13269 | 4.02E-27 | 0.494171 | 9.66E-23 | In |
| Nsg2 | 3.62E-20 | 0.493552 | 8.69E-16 | In |
| Ypel2 | 1.41E-16 | 0.492139 | 3.39E-12 | In |
| Chst11 | 1.07E-28 | 0.490467 | 2.57E-24 | In |
| Gm15810 | 1.59E-32 | 0.487895 | 3.82E-28 | In |
| Kalrn | 1.29E-29 | 0.487448 | 3.1E-25 | In |
| Mpp7 | 4.42E-20 | 0.486152 | 1.06E-15 | In |
| Mbnl2 | 5.45E-16 | 0.48542 | 1.31E-11 | In |
| Fat3 | 3.86E-18 | 0.479914 | 9.26E-14 | In |
| Stk32a | 2.73E-28 | 0.477555 | 6.56E-24 | In |
| Unc79 | 1.05E-28 | 0.477488 | 2.51E-24 | In |
| Anks1b | 4.94E-40 | 0.476929 | 1.19E-35 | In |
| Khdrbs2 | 6.26E-18 | 0.476411 | 1.5E-13 | In |
| Tgfa | 1E-41 | 0.476078 | 2.4E-37 | In |
| Spock3 | 1.22E-09 | 0.474424 | 2.92E-05 | In |
| Onecut2 | 4.42E-29 | 0.474361 | 1.06E-24 | In |
| Prkd1 | 2.78E-12 | 0.47335 | 6.67E-08 | In |
| Tenm3 | 3.57E-09 | 0.468551 | 8.57E-05 | In |
| Cacng4 | 1.08E-28 | 0.46828 | 2.59E-24 | In |
| Gm15155 | 3.92E-14 | 0.460706 | 9.4E-10 | In |
| Scn3a | 2.82E-24 | 0.46049 | 6.77E-20 | In |
| Ddx5 | 2.17E-20 | 0.457837 | 5.22E-16 | In |
| Snca | 1.19E-18 | 0.455916 | 2.84E-14 | In |
| G630016G05Rik | 6.98E-31 | 0.455184 | 1.67E-26 | In |
| Myt1l | 1.13E-32 | 0.452718 | 2.72E-28 | In |
| Sgk3 | 5.19E-20 | 0.452698 | 1.25E-15 | In |
| Ric8b | 7.62E-18 | 0.448294 | 1.83E-13 | In |
| Rtn1 | 4.52E-23 | 0.447046 | 1.09E-18 | In |
| Acvr1c | 5.75E-35 | 0.446607 | 1.38E-30 | In |
| Mctp1 | 1.27E-11 | 0.444907 | 3.06E-07 | In |
| Rfx3 | 6.52E-17 | 0.444643 | 1.56E-12 | In |
| Dock10 | 7.13E-14 | 0.444369 | 1.71E-09 | In |
| Cacna1c | 5.84E-12 | 0.442813 | 1.4E-07 | In |
| Elmod1 | 5.35E-15 | 0.44108 | 1.28E-10 | In |
| Zfp462 | 1.3E-14 | 0.439376 | 3.11E-10 | In |
| Aff3 | 4.82E-16 | 0.435761 | 1.16E-11 | In |
| Robo2 | 8.38E-07 | 0.434932 | 0.02012 | In |
| Tle4 | 1.05E-08 | 0.432451 | 0.000252 | In |
| Usp3 | 1.69E-17 | 0.432431 | 4.06E-13 | In |
| Arhgef9 | 2.16E-18 | 0.431379 | 5.18E-14 | In |
| Strip2 | 1.79E-34 | 0.430326 | 4.29E-30 | In |
| Csmd3 | 2.25E-21 | 0.429597 | 5.41E-17 | In |
| R3hdm1 | 5.16E-19 | 0.426658 | 1.24E-14 | In |
| B3galt1 | 2E-19 | 0.425391 | 4.8E-15 | In |
| Klhl1 | 1.04E-12 | 0.42252 | 2.49E-08 | In |
| Sertad2 | 1.19E-17 | 0.419128 | 2.85E-13 | In |
| Macrod2 | 4.7E-26 | 0.411916 | 1.13E-21 | In |
| Ablim2 | 2.16E-07 | 0.409584 | 0.005183 | In |
| Alcam | 1.34E-06 | 0.408976 | 0.032209 | In |
| Igf1r | 7.45E-08 | 0.408819 | 0.001788 | In |
| S100pbp | 6.93E-24 | 0.407552 | 1.66E-19 | In |
| Syn3 | 9.3E-12 | 0.407068 | 2.23E-07 | In |
| Akap9 | 2.49E-14 | 0.406518 | 5.97E-10 | In |
| Tenm2 | 1.05E-12 | 0.406505 | 2.53E-08 | In |
| Grid1 | 5.05E-16 | 0.400931 | 1.21E-11 | In |
| Camk2b | 5.1E-16 | 0.400647 | 1.22E-11 | In |
| Map2 | 3.93E-09 | 0.399784 | 9.44E-05 | In |
| Gsg1l | 7.72E-18 | 0.398691 | 1.85E-13 | In |
| Ptpn5 | 3.82E-14 | 0.398095 | 9.18E-10 | In |
| Grm5 | 2.39E-12 | 0.396411 | 5.73E-08 | In |
| Osbp2 | 1.19E-13 | 0.393707 | 2.85E-09 | In |
| Ppp2r2a | 7.82E-15 | 0.391638 | 1.88E-10 | In |
| Rbms1 | 7.45E-14 | 0.391394 | 1.79E-09 | In |
| Sncaip | 2.17E-08 | 0.389024 | 0.00052 | In |
| Sorcs2 | 3.83E-15 | 0.388785 | 9.18E-11 | In |
| A330015K06Rik | 1.32E-10 | 0.38765 | 3.18E-06 | In |
| Kcnk2 | 3.47E-10 | 0.387395 | 8.33E-06 | In |
| Adam23 | 1.81E-12 | 0.386824 | 4.35E-08 | In |
| Slc6a6 | 3.11E-15 | 0.386108 | 7.48E-11 | In |
| Runx1t1 | 1.93E-08 | 0.383737 | 0.000463 | In |
| Jarid2 | 1.04E-06 | 0.383342 | 0.02488 | In |
| Nptn | 4.59E-20 | 0.381613 | 1.1E-15 | In |
| Kat6b | 6.88E-09 | 0.379818 | 0.000165 | In |
| Rora | 3.76E-10 | 0.377192 | 9.03E-06 | In |
| Nexn | 4.19E-21 | 0.376026 | 1.01E-16 | In |
| Nell2 | 6.24E-10 | 0.375804 | 1.5E-05 | In |
| Ankrd6 | 2.24E-19 | 0.374901 | 5.37E-15 | In |
| Rasgrp1 | 2.64E-22 | 0.373165 | 6.33E-18 | In |
| Atf6 | 1.44E-14 | 0.370403 | 3.47E-10 | In |
| Cpne8 | 4.52E-14 | 0.370318 | 1.08E-09 | In |
| Kcnip3 | 1.73E-14 | 0.368894 | 4.15E-10 | In |
| Kcnip2 | 4.41E-16 | 0.368474 | 1.06E-11 | In |
| Arl15 | 1.18E-09 | 0.367644 | 2.84E-05 | In |
| Peli1 | 1.35E-09 | 0.366137 | 3.25E-05 | In |
| Zmiz1 | 3.99E-07 | 0.365705 | 0.009576 | In |
| Dlgap3 | 3.04E-12 | 0.364092 | 7.29E-08 | In |
| Klf7 | 1.63E-11 | 0.363826 | 3.92E-07 | In |
| Psd3 | 7.64E-12 | 0.362452 | 1.83E-07 | In |
| Plekha5 | 5.53E-18 | 0.361638 | 1.33E-13 | In |
| Zfp385b | 1.7E-11 | 0.361132 | 4.07E-07 | In |
| Gm45159 | 2.64E-13 | 0.360341 | 6.34E-09 | In |
| Cyld | 2.09E-10 | 0.358555 | 5.02E-06 | In |
| Prkar2b | 1.5E-12 | 0.358135 | 3.6E-08 | In |
| Jak2 | 1.5E-17 | 0.356473 | 3.61E-13 | In |
| Ano2 | 1.83E-30 | 0.355759 | 4.38E-26 | In |
| Epb41 | 2.58E-11 | 0.354744 | 6.19E-07 | In |
| Gabrb3 | 2.95E-20 | 0.354299 | 7.07E-16 | In |
| Sipa1l1 | 2.84E-19 | 0.353831 | 6.83E-15 | In |
| Rcan2 | 1.18E-08 | 0.353107 | 0.000284 | In |
| Gm15738 | 1.84E-13 | 0.351875 | 4.42E-09 | In |
| Cttnbp2 | 1.07E-14 | 0.351116 | 2.58E-10 | In |
| Zswim5 | 4.28E-13 | 0.3509 | 1.03E-08 | In |
| Ezh2 | 1.56E-09 | 0.350725 | 3.75E-05 | In |
| Grid2 | 1E-09 | 0.350498 | 2.4E-05 | In |
| Lin7a | 4.04E-11 | 0.350084 | 9.7E-07 | In |
| Zc3h12c | 3.7E-09 | 0.346521 | 8.89E-05 | In |
| Plcb1 | 4.58E-12 | 0.344993 | 1.1E-07 | In |
| Tnik | 2.26E-18 | 0.343604 | 5.43E-14 | In |
| Fgf14 | 4.22E-28 | 0.342907 | 1.01E-23 | In |
| Xrcc4 | 2.31E-12 | 0.34225 | 5.55E-08 | In |
| Dtnb | 7.58E-12 | 0.340902 | 1.82E-07 | In |
| Zfhx4 | 5.8E-29 | 0.340699 | 1.39E-24 | In |
| Kcna4 | 3.98E-23 | 0.339898 | 9.54E-19 | In |
| Bcr | 1.61E-13 | 0.339714 | 3.86E-09 | In |
| Zfp407 | 1.1E-10 | 0.337708 | 2.64E-06 | In |
| Dchs2 | 2.65E-12 | 0.335113 | 6.36E-08 | In |
| Gabra4 | 9.52E-10 | 0.332305 | 2.28E-05 | In |
| Car12 | 1.46E-30 | 0.332229 | 3.51E-26 | In |
| Gcnt2 | 4.6E-18 | 0.33122 | 1.1E-13 | In |
| Rps6ka3 | 5.54E-08 | 0.328686 | 0.001329 | In |
| Cacna2d1 | 6.34E-26 | 0.327508 | 1.52E-21 | In |
| Trerf1 | 1.32E-09 | 0.326752 | 3.16E-05 | In |
| Chsy3 | 4.06E-07 | 0.323786 | 0.009755 | In |
| Myt1 | 3.51E-08 | 0.321868 | 0.000842 | In |
| Erc2 | 1.89E-15 | 0.32096 | 4.52E-11 | In |
| Kdm3a | 3.37E-10 | 0.319883 | 8.1E-06 | In |
| Tmtc1 | 3.08E-10 | 0.319646 | 7.4E-06 | In |
| Plp1 | 1.03E-10 | 0.318859 | 2.48E-06 | In |
| Nova1 | 2.85E-10 | 0.314051 | 6.84E-06 | In |
| Celf1 | 1.41E-10 | 0.312747 | 3.38E-06 | In |
| Ank3 | 2.61E-19 | 0.312709 | 6.27E-15 | In |
| Fam184b | 7.54E-12 | 0.312433 | 1.81E-07 | In |
| Sorbs2 | 9.42E-12 | 0.312239 | 2.26E-07 | In |
| Ext1 | 7.05E-09 | 0.310789 | 0.000169 | In |
| Sv2b | 2.42E-11 | 0.309167 | 5.81E-07 | In |
| Focad | 2.82E-08 | 0.307407 | 0.000676 | In |
| Ptch1 | 4.02E-12 | 0.305034 | 9.65E-08 | In |
| Add2 | 8.34E-10 | 0.305019 | 2E-05 | In |
| Arhgap32 | 1.67E-10 | 0.302919 | 4.02E-06 | In |
| Ctnna2 | 3.51E-10 | 0.301977 | 8.43E-06 | In |
| Tmeff1 | 4.17E-09 | 0.301752 | 0.0001 | In |
| Lrrtm3 | 4.68E-10 | 0.301627 | 1.12E-05 | In |
| Gulp1 | 1.24E-17 | 0.300614 | 2.98E-13 | In |
| Rab40b | 3.79E-14 | 0.299793 | 9.1E-10 | In |
| Tusc3 | 1E-10 | 0.298639 | 2.41E-06 | In |
| Gdpd5 | 1.19E-20 | 0.298365 | 2.85E-16 | In |
| Arid1b | 2.74E-09 | 0.29803 | 6.58E-05 | In |
| Man1a | 7.69E-14 | 0.297432 | 1.85E-09 | In |
| Slco5a1 | 6.11E-13 | 0.297336 | 1.47E-08 | In |
| B3galt5 | 4.97E-27 | 0.29669 | 1.19E-22 | In |
| Rap1gap | 8.24E-07 | 0.295137 | 0.01978 | In |
| Spata13 | 1.41E-26 | 0.294278 | 3.39E-22 | In |
| 1700003D09Rik | 6.9E-29 | 0.293406 | 1.66E-24 | In |
| Zfp609 | 2.79E-08 | 0.291199 | 0.00067 | In |
| Cdk19 | 5.57E-08 | 0.289993 | 0.001338 | In |
| Rxrg | 7.66E-22 | 0.286914 | 1.84E-17 | In |
| Wasf1 | 2.26E-11 | 0.286017 | 5.42E-07 | In |
| Ncam2 | 2.23E-10 | 0.285753 | 5.34E-06 | In |
| Sorcs1 | 9.27E-18 | 0.284803 | 2.23E-13 | In |
| Rgs17 | 9.64E-09 | 0.284567 | 0.000231 | In |
| Phex | 6.43E-18 | 0.284288 | 1.54E-13 | In |
| Dcbld1 | 1.02E-11 | 0.283886 | 2.45E-07 | In |
| Rit2 | 9.2E-09 | 0.282337 | 0.000221 | In |
| Kcnb1 | 6.99E-11 | 0.281062 | 1.68E-06 | In |
| Nkain2 | 8.79E-12 | 0.28038 | 2.11E-07 | In |
| Zfp827 | 1.95E-09 | 0.279919 | 4.69E-05 | In |
| Cacng3 | 3.63E-08 | 0.2794 | 0.000871 | In |
| Negr1 | 6.61E-13 | 0.279068 | 1.59E-08 | In |
| Fam102b | 3.61E-15 | 0.278942 | 8.66E-11 | In |
| Fnbp1l | 6.56E-09 | 0.277774 | 0.000157 | In |
| Gria2 | 4.86E-18 | 0.27626 | 1.17E-13 | In |
| Gm47591 | 9.59E-14 | 0.276099 | 2.3E-09 | In |
| Gm38413 | 5.49E-27 | 0.27362 | 1.32E-22 | In |
| Mme | 9.33E-12 | 0.272858 | 2.24E-07 | In |
| Pcmtd1 | 1.93E-09 | 0.272803 | 4.63E-05 | In |
| Pced1b | 2.34E-14 | 0.272115 | 5.6E-10 | In |
| St8sia2 | 1.17E-08 | 0.27088 | 0.000282 | In |
| Mark2 | 5.82E-10 | 0.270686 | 1.4E-05 | In |
| 4933424G05Rik | 5.16E-14 | 0.269363 | 1.24E-09 | In |
| Kcnh8 | 3.4E-08 | 0.268332 | 0.000815 | In |
| Brd1 | 9.75E-14 | 0.267646 | 2.34E-09 | In |
| Grm1 | 1.47E-07 | 0.267589 | 0.003539 | In |
| Nrp1 | 4.68E-12 | 0.266918 | 1.12E-07 | In |
| Dapk1 | 4.26E-08 | 0.265724 | 0.001022 | In |
| Syndig1 | 7.25E-07 | 0.265351 | 0.017403 | In |
| Scn4b | 3.56E-25 | 0.26434 | 8.56E-21 | In |
| Gm50024 | 1.84E-29 | 0.264305 | 4.42E-25 | In |
| Gm10115 | 7.57E-20 | 0.263113 | 1.82E-15 | In |
| Msra | 6.79E-07 | 0.262015 | 0.016305 | In |
| Cadm2 | 3.46E-15 | 0.261719 | 8.3E-11 | In |
| Adcy9 | 2.87E-09 | 0.261611 | 6.88E-05 | In |
| Dclk3 | 4.73E-24 | 0.261071 | 1.14E-19 | In |
| Itgav | 3.31E-12 | 0.260934 | 7.94E-08 | In |
| B3gnt2 | 2.9E-18 | 0.260597 | 6.95E-14 | In |
| Htr2c | 4.94E-15 | 0.260091 | 1.18E-10 | In |
| Akap6 | 2.24E-13 | 0.259043 | 5.38E-09 | In |
| Nrxn3 | 4.86E-09 | 0.259029 | 0.000117 | In |
| Eml5 | 3.35E-08 | 0.257952 | 0.000803 | In |
| Ankrd28 | 2.95E-07 | 0.256535 | 0.007076 | In |
| Ror1 | 7.91E-13 | 0.256184 | 1.9E-08 | In |
| Zfp521 | 5.06E-11 | 0.255094 | 1.22E-06 | In |
| Ptprd | 1.72E-07 | 0.254778 | 0.00414 | In |
| Adora2a | 8.64E-21 | 0.254565 | 2.07E-16 | In |
| Rassf8 | 2.34E-12 | 0.2533 | 5.61E-08 | In |
| Cachd1 | 4.17E-07 | 0.253016 | 0.010012 | In |
| Fndc3b | 2.85E-08 | 0.251757 | 0.000685 | In |
| Mei4 | 1.75E-10 | 0.250259 | 4.2E-06 | In |
| mt-Atp6 | 0 | -1.13202652229904 | 0 | Ex |
| mt-Co1 | 0 | -1.09832109186864 | 0 | Ex |
| mt-Co2 | 0 | -1.05951863826756 | 0 | Ex |
| Mid1 | 0 | -1.04158358112216 | 0 | Ex |
| mt-Co3 | 0 | -1.03973937882363 | 0 | Ex |
| mt-Cytb | 0 | -0.993950798356582 | 0 | Ex |
| mt-Nd2 | 0 | -0.984010021969966 | 0 | Ex |
| mt-Nd4 | 0 | -0.96786736477242 | 0 | Ex |
| mt-Nd1 | 4E-212 | -0.818371775306181 | 9.6E-208 | Ex |
| Fth1 | 2.2E-118 | -0.781443044556077 | 5.2E-114 | Ex |
| Apoe | 8.2E-149 | -0.606606467608202 | 2E-144 | Ex |
| mt-Nd3 | 1.5E-112 | -0.563627147719699 | 3.6E-108 | Ex |
| Homer1 | 1.02E-41 | -0.556737645186935 | 2.45E-37 | Ex |
| mt-Nd5 | 6.7E-127 | -0.538595818174029 | 1.6E-122 | Ex |
| 1700016P03Rik | 2.94E-43 | -0.496806422426154 | 7.05E-39 | Ex |
| Mbp | 4.93E-27 | -0.492805187932223 | 1.18E-22 | Ex |
| Cst3 | 3.68E-94 | -0.463704777864047 | 8.83E-90 | Ex |
| mt-Nd4l | 6.33E-65 | -0.39895922990655 | 1.52E-60 | Ex |
| Cox8a | 3.63E-57 | -0.390698646175347 | 8.71E-53 | Ex |
| Ckb | 7.35E-66 | -0.374886135450459 | 1.76E-61 | Ex |
| Rps29 | 5.75E-53 | -0.324561788360452 | 1.38E-48 | Ex |
| Camk2n1 | 3.03E-47 | -0.320272292863985 | 7.28E-43 | Ex |
| Cox4i1 | 1.29E-33 | -0.31209253144468 | 3.09E-29 | Ex |
| Sik2 | 2.44E-23 | -0.303231406626933 | 5.86E-19 | Ex |
| Rps21 | 8.93E-40 | -0.294993101686371 | 2.14E-35 | Ex |
| Rplp1 | 2.32E-61 | -0.294727558404102 | 5.56E-57 | Ex |
| Mt1 | 2.35E-63 | -0.292028151859709 | 5.64E-59 | Ex |
| Pcsk1 | 8.51E-15 | -0.291175817027022 | 2.04E-10 | Ex |
| Pcsk1n | 4.49E-61 | -0.28858986061972 | 1.08E-56 | Ex |
| Rpsa | 5.46E-42 | -0.28609126587242 | 1.31E-37 | Ex |
| Calm1 | 2.73E-42 | -0.275288768384079 | 6.55E-38 | Ex |
| Rpl13 | 2.16E-41 | -0.264291839444429 | 5.19E-37 | Ex |
| Egr3 | 6.46E-28 | -0.250572381619213 | 1.55E-23 | Ex |
| 6530403H02Rik | 4.06E-12 | 0.206702 | 7.61E-08 | Ex |
| Mctp1 | 1.23E-14 | 0.230213 | 2.31E-10 | Ex |
| Brd1 | 4.97E-73 | 0.246132 | 9.3E-69 | Ex |

**Supplementary Table S5. GO and KEGG analysis of DEGs.**

| ID | Description | GeneRatio | BgRatio | pvalue | p_adjust |
| --- | --- | --- | --- | --- | --- |
| mmu04724 | Glutamatergic synapse | 24/252 | 113/8996 | 4.36E-15 | 9.81E-13 |
| mmu05032 | Morphine addiction | 20/252 | 91/8996 | 4.63E-13 | 5.21E-11 |
| mmu04921 | Oxytocin signaling pathway | 22/252 | 153/8996 | 2.24E-10 | 1.68E-08 |
| mmu04020 | Calcium signaling pathway | 27/252 | 240/8996 | 5.68E-10 | 3.19E-08 |
| mmu04723 | Retrograde endocannabinoid signaling | 20/252 | 148/8996 | 4.63E-09 | 2.09E-07 |
| mmu04742 | Taste transduction | 15/252 | 92/8996 | 3.23E-08 | 1.21E-06 |
| mmu04261 | Adrenergic signaling in cardiomyocytes | 19/252 | 152/8996 | 4.17E-08 | 1.34E-06 |
| mmu04713 | Circadian entrainment | 15/252 | 98/8996 | 7.76E-08 | 2.18E-06 |
| mmu04727 | GABAergic synapse | 14/252 | 89/8996 | 1.50E-07 | 3.74E-06 |
| mmu04720 | Long-term potentiation | 12/252 | 67/8996 | 2.74E-07 | 6.17E-06 |
| mmu04725 | Cholinergic synapse | 15/252 | 112/8996 | 4.69E-07 | 9.60E-06 |
| mmu04010 | MAPK signaling pathway | 25/252 | 294/8996 | 6.66E-07 | 1.25E-05 |
| mmu04728 | Dopaminergic synapse | 16/252 | 135/8996 | 1.05E-06 | 1.81E-05 |
| mmu05033 | Nicotine addiction | 9/252 | 40/8996 | 1.18E-06 | 1.89E-05 |
| mmu05414 | Dilated cardiomyopathy | 13/252 | 94/8996 | 1.92E-06 | 2.88E-05 |
| mmu04360 | Axon guidance | 18/252 | 181/8996 | 2.94E-06 | 4.14E-05 |
| mmu04024 | cAMP signaling pathway | 20/252 | 220/8996 | 3.33E-06 | 4.21E-05 |
| mmu04080 | Neuroactive ligand-receptor interaction | 28/252 | 386/8996 | 3.37E-06 | 4.21E-05 |
| mmu04911 | Insulin secretion | 12/252 | 86/8996 | 4.36E-06 | 5.17E-05 |
| mmu04925 | Aldosterone synthesis and secretion | 13/252 | 102/8996 | 4.88E-06 | 5.49E-05 |
| GO:0050808 | synapse organization | 76/617 | 494/28814 | 2.87E-42 | 1.21E-38 |
| GO:0016358 | dendrite development | 52/617 | 317/28814 | 1.71E-30 | 3.58E-27 |
| GO:0034765 | regulation of ion transmembrane transport | 59/617 | 498/28814 | 1.09E-26 | 1.52E-23 |
| GO:0042391 | regulation of membrane potential | 57/617 | 469/28814 | 2.21E-26 | 2.32E-23 |
| GO:0007626 | locomotory behavior | 42/617 | 265/28814 | 3.20E-24 | 2.69E-21 |
| GO:0035249 | synaptic transmission, glutamatergic | 28/617 | 117/28814 | 1.32E-21 | 9.23E-19 |
| GO:0048813 | dendrite morphogenesis | 32/617 | 187/28814 | 8.89E-20 | 5.33E-17 |
| GO:1904062 | regulation of cation transmembrane transport | 44/617 | 381/28814 | 1.02E-19 | 5.33E-17 |
| GO:0034329 | cell junction assembly | 47/617 | 441/28814 | 1.52E-19 | 6.65E-17 |
| GO:0032409 | regulation of transporter activity | 39/617 | 298/28814 | 1.58E-19 | 6.65E-17 |
| GO:0022898 | regulation of transmembrane transporter activity | 38/617 | 284/28814 | 2.15E-19 | 7.92E-17 |
| GO:0099173 | postsynapse organization | 33/617 | 207/28814 | 2.26E-19 | 7.92E-17 |
| GO:2001257 | regulation of cation channel activity | 31/617 | 181/28814 | 3.27E-19 | 1.06E-16 |
| GO:0050890 | cognition | 42/617 | 359/28814 | 4.37E-19 | 1.24E-16 |
| GO:0006836 | neurotransmitter transport | 35/617 | 242/28814 | 4.62E-19 | 1.24E-16 |
| GO:0032412 | regulation of ion transmembrane transporter activity | 37/617 | 274/28814 | 4.73E-19 | 1.24E-16 |
| GO:0050807 | regulation of synapse organization | 36/617 | 263/28814 | 9.11E-19 | 2.25E-16 |
| GO:0007416 | synapse assembly | 31/617 | 193/28814 | 2.26E-18 | 5.26E-16 |
| GO:0050803 | regulation of synapse structure or activity | 36/617 | 271/28814 | 2.47E-18 | 5.45E-16 |
| GO:0060996 | dendritic spine development | 26/617 | 130/28814 | 4.58E-18 | 9.62E-16 |
| GO:0097060 | synaptic membrane | 81/620 | 390/28739 | 2.63E-55 | 1.11E-52 |
| GO:0098984 | neuron to neuron synapse | 77/620 | 361/28739 | 1.76E-53 | 3.70E-51 |
| GO:0032279 | asymmetric synapse | 74/620 | 339/28739 | 3.64E-52 | 5.10E-50 |
| GO:0099572 | postsynaptic specialization | 74/620 | 352/28739 | 6.48E-51 | 6.82E-49 |
| GO:0014069 | postsynaptic density | 70/620 | 330/28739 | 1.89E-48 | 1.59E-46 |
| GO:0098978 | glutamatergic synapse | 69/620 | 323/28739 | 5.59E-48 | 3.93E-46 |
| GO:0045211 | postsynaptic membrane | 59/620 | 273/28739 | 2.14E-41 | 1.29E-39 |
| GO:0098793 | presynapse | 64/620 | 493/28739 | 5.07E-31 | 2.67E-29 |
| GO:0034702 | ion channel complex | 49/620 | 294/28739 | 5.69E-29 | 2.66E-27 |
| GO:0034703 | cation channel complex | 41/620 | 224/28739 | 4.70E-26 | 1.98E-24 |
| GO:0044309 | neuron spine | 39/620 | 203/28739 | 1.17E-25 | 4.48E-24 |
| GO:1902495 | transmembrane transporter complex | 49/620 | 372/28739 | 3.13E-24 | 1.10E-22 |
| GO:0043197 | dendritic spine | 37/620 | 198/28739 | 5.86E-24 | 1.90E-22 |
| GO:0099240 | intrinsic component of synaptic membrane | 30/620 | 123/28739 | 3.16E-23 | 9.51E-22 |
| GO:1990351 | transporter complex | 49/620 | 393/28739 | 3.65E-23 | 1.03E-21 |
| GO:0042734 | presynaptic membrane | 30/620 | 132/28739 | 2.92E-22 | 7.70E-21 |
| GO:0099699 | integral component of synaptic membrane | 28/620 | 113/28739 | 5.74E-22 | 1.42E-20 |
| GO:0099634 | postsynaptic specialization membrane | 23/620 | 85/28739 | 3.18E-19 | 7.43E-18 |
| GO:0098685 | Schaffer collateral - CA1 synapse | 22/620 | 83/28739 | 3.11E-18 | 6.89E-17 |
| GO:0099055 | integral component of postsynaptic membrane | 22/620 | 85/28739 | 5.46E-18 | 1.15E-16 |
| GO:0022836 | gated channel activity | 49/609 | 323/28275 | 4.35E-27 | 2.02E-24 |
| GO:0005216 | ion channel activity | 56/609 | 438/28275 | 6.06E-27 | 2.02E-24 |
| GO:0015267 | channel activity | 56/609 | 482/28275 | 7.70E-25 | 1.28E-22 |
| GO:0022803 | passive transmembrane transporter activity | 56/609 | 482/28275 | 7.70E-25 | 1.28E-22 |
| GO:0005261 | cation channel activity | 45/609 | 333/28275 | 7.72E-23 | 1.03E-20 |
| GO:0046873 | metal ion transmembrane transporter activity | 49/609 | 421/28275 | 6.91E-22 | 7.67E-20 |
| GO:0005244 | voltage-gated ion channel activity | 34/609 | 192/28275 | 2.34E-21 | 2.23E-19 |
| GO:0022832 | voltage-gated channel activity | 34/609 | 193/28275 | 2.79E-21 | 2.32E-19 |
| GO:0022843 | voltage-gated cation channel activity | 29/609 | 142/28275 | 3.36E-20 | 2.49E-18 |
| GO:0005516 | calmodulin binding | 30/609 | 192/28275 | 2.06E-17 | 1.37E-15 |
| GO:0030695 | GTPase regulator activity | 42/609 | 422/28275 | 1.97E-16 | 1.10E-14 |
| GO:0060589 | nucleoside-triphosphatase regulator activity | 42/609 | 422/28275 | 1.97E-16 | 1.10E-14 |
| GO:0044325 | transmembrane transporter binding | 24/609 | 147/28275 | 1.23E-14 | 6.30E-13 |
| GO:0003779 | actin binding | 39/609 | 444/28275 | 1.41E-13 | 6.68E-12 |
| GO:0005267 | potassium channel activity | 21/609 | 121/28275 | 1.61E-13 | 7.17E-12 |
| GO:0015079 | potassium ion transmembrane transporter activity | 23/609 | 157/28275 | 4.72E-13 | 1.97E-11 |
| GO:0016247 | channel regulator activity | 22/609 | 144/28275 | 6.36E-13 | 2.49E-11 |
| GO:0099106 | ion channel regulator activity | 21/609 | 136/28275 | 1.72E-12 | 6.38E-11 |
| GO:0098960 | postsynaptic neurotransmitter receptor activity | 15/609 | 69/28275 | 1.63E-11 | 5.71E-10 |
| GO:0005249 | voltage-gated potassium channel activity | 16/609 | 88/28275 | 6.09E-11 | 1.95E-09 |
